# Natural History of a Satellite DNA Family: From the Ancestral Genome Component to Species-Specific Sequences, Concerted and Non-Concerted Evolution

**DOI:** 10.3390/ijms20051201

**Published:** 2019-03-09

**Authors:** Alexander Belyayev, Jiřina Josefiová, Michaela Jandová, Ruslan Kalendar, Karol Krak, Bohumil Mandák

**Affiliations:** 1 The Czech Academy of Sciences, Institute of Botany, Zámek 1, 252 43 Průhonice, Czech Republic; jirina.josefiova@ibot.cas.cz (J.J.); michaela.jandova@ibot.cas.cz (M.J.); karol.krak@ibot.cas.cz (K.K.); bohumil.mandak@ibot.cas.cz (B.M.); 2 Department of Agricultural Sciences, University of Helsinki, P.O. Box 27 (Latokartanonkaari 5), 00014 Helsinki, Finland; ruslan.kalendar@helsinki.fi; 3 RSE “National Center for Biotechnology”, 13/5, Kurgalzhynskoye road, Astana 010000, Kazakhstan; 4 Faculty of Environmental Sciences, Czech University of Life Sciences Prague, Kamýcká 129, 165 00 Praha-Suchdol, Czech Republic

**Keywords:** satellite DNA, genome evolution, plants, next-generation sequencing, high order repeats

## Abstract

Satellite DNA (satDNA) is the most variable fraction of the eukaryotic genome. Related species share a common ancestral satDNA library and changing of any library component in a particular lineage results in interspecific differences. Although the general developmental trend is clear, our knowledge of the origin and dynamics of satDNAs is still fragmentary. Here, we explore whole genome shotgun Illumina reads using the RepeatExplorer (RE) pipeline to infer satDNA family life stories in the genomes of *Chenopodium* species. The seven diploids studied represent separate lineages and provide an example of a species complex typical for angiosperms. Application of the RE pipeline allowed by similarity searches a determination of the satDNA family with a basic monomer of ~40 bp and to trace its transformation from the reconstructed ancestral to the species-specific sequences. As a result, three types of satDNA family evolutionary development were distinguished: (i) concerted evolution with mutation and recombination events; (ii) concerted evolution with a trend toward increased complexity and length of the satellite monomer; and (iii) non-concerted evolution, with low levels of homogenization and multidirectional trends. The third type is an example of entire repeatome transformation, thus producing a novel set of satDNA families, and genomes showing non-concerted evolution are proposed as a significant source for genomic diversity.

## 1. Introduction

Genome evolution can be defined as the multifactorial process of variation of nuclear genome components over time. The process is heterogeneous, and different genomic fractions evolve at different rates. The most rapid changes were recorded for repeatomes, which form the basis of most eukaryotic genomes and consist of repeated and repeat-derived sequences [1,2]. As a subject of concerted evolution, the repeatomes of diverging species mostly change non-independently in a concerted way those results in a sequence similarity of repeating units greater within than among species [3]. Repetitive DNA complexes play an important role in evolutionary genome transformation, and determination of their origin, composition and dynamics is crucial for understanding genomic diversity [4].

The repeatome consists of several large classes, among which transposable elements (TEs) and satellite DNA (satDNA) predominate [5,6]. The latter consists of long, late-replicating, non-coding arrays of tandemly arranged monomers [5,7]. These sequences are often species or genus specific and are considered the most variable fraction of the eukaryotic genome, thus reflecting trajectories of short-term evolutionary change [8,9,10,11]. Recent studies suggest that satDNA, which is predominantly concentrated in the heterochromatic regions of chromosomes, is involved in various functions ranging from chromosome organization and pairing to cell metabolism and adjustment of gene functions [12,13,14,15,16]. Despite their particular importance for understanding genome functioning and restructuring during micro- and macroevolutionary processes and the growing awareness of their structure and functional significance, knowledge on the origin and dynamics of satDNA is fragmentary, especially in non-model species. 

It is generally accepted that an intraspecific monomer change in various satDNA families is permanent [17]. Related species share a common satDNA library that was present in the common ancestor. Differential amplification of satellites from this library and acquisition of mutations in diverse lineages results in interspecific differences in that fraction [18]. Spreading of a new variant processed by non-Mendelian molecular mechanisms is followed by the fixation of the new variant within a population by sexual reproduction [19,20,21]. Thus, intraspecific homogenization of the satDNA family and fixation of species-specific polymorphisms occur simultaneously [22], and the main trend of satDNA conversion can be considered as a transformation from the common ancestral to the species-specific tandem repeats. The process appears to be a significant part of speciation at the molecular level [4]. Recently, the possibility of unraveling details of this ubiquitous phenomenon by next-generation sequencing (NGS) technology appeared through comparative analysis of the entire species repeatome. Importantly, this method is applicable not only for model organisms but also for a wide range of wild species, which allows the construction of a generalized model.

**Figure 1:**
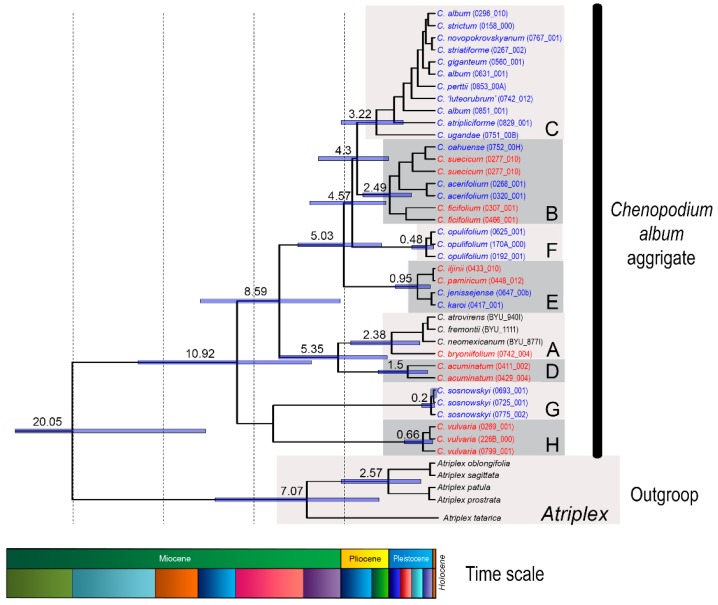
Phylogenetic tree calculated using Bayesian inference within the *C. album* aggregate estimated based on the concatenated dataset of three chloroplast DNA spacers (adapted from [27]). Major evolutionary lineages (A–H) are marked by grey rectangles. The numbers above branches correspond to the ages of the particular clades (in millions of years) as inferred by the analysis in BEAST2. Positions of explored diploid species are shown in red. Polyploid species are shown in blue. The schematic stratigraphic time scale (Miocene–Holocene) is shown at the bottom of the figure.

In the present study, we sought to explore NGS data using the RepeatExplorer (RE) pipeline [23] to infer satDNA evolutionary dynamics in the genomes of *Chenopodium* s. str. (also referred to as the *Chenopodium album* aggregate). Species of the *C. album* aggregate are distributed worldwide, with the highest species diversity in temperate areas [24]. The majority of these diploid-polyploid species are phenotypically exceptionally plastic [25], in some cases widely distributed and able to grow under a wide range of conditions [26]. We focused on diploid species (2*n* = 2*x* = 18) of the aggregate that represent separate lineages. Specifically,: (i) “clade A” are the species native to America and East Asia (the latter area being represented by *C. bryoniifolium* Bunge); (ii) “clade B” of the Eurasian temperate species *C. ficifolium* Sm. and the boreal species *C. suecicum* Murr.; (iii) “clade D” comprising the only East and Central Asian species, *C. acuminatum* Willd; (iv) “clade E” represented by the Central Asian *C. pamiricum* Iljin and *C. iljinii* Golosk.; (v) “clade H” comprising presumably European and southwest Asian species *C. vulvaria* L; and clades C, F and G consist of polyploid species. By the existence of basic diploid lineages, the origin of the majority of Eurasian polyploid species can be explained as hybridization among the diploid lineages that created subgenomic combinations of individual polyploid taxa (see [27] for details) (Figure 1). This group was selected based on the following two criteria: (i) analyzed species of the genus *Chenopodium* provide an example of a diploid/polyploid complex [26,27] that is very typical for angiosperms and, to a certain extent, can be regarded as a standard model for the divergent evolution of higher plants; and (ii) a basic repeat unit with pan-chromosomal distribution and also related to the satellite monomer of *Beta corolliflora* was previously found in the genome of a *Chenopodium* species [28,29]. This combination of favorable factors makes the study promising for describing satDNA family evolution in a typical group of flowering plants. Given the worldwide distribution of the *C. album* aggregate and its tens of millions of years of evolution [27], we hypothesize the presence of different types of satDNA family transformations in diverged lineages.

## 2. Results

### 2.1. Clustering Results and Identification of satDNA Clusters 

Application of the RE pipeline clustering tool for Illumina reads of seven diploid *Chenopodium* species (Table 1) (genome coverage 41.3–58.2%) resulted in the identification of clusters that represent different families of TEs, their derivatives and satDNAs. The latter was the main aim of our research. Several valuable outcomes from the present study are shown in Table 2. *C. vulvaria* and *C. acuminatum* possess the smallest genomes in the group, while those of *C. ficifolium* and *C. suecicum* are the largest. *C. vulvaria* exceeds all investigated species in the number of RE clusters and RE singlets, which emphasizes for its genome diversity.

The satellite monomer of ~40 bp was found during the RE analysis of satDNA in each genome of analyzed species. According to BLAST results, these monomers were related to each other and to the tribe-specific repetitive sequence (GenBank ID HM641822.1), found in *Chenopodium quinoa* by Kolano et al. [29], and to the satellite sequence with the GenBank ID AJ288880.1, which was found in *Beta corolliflora* by Gao et al. [28] (S1). It was thus assumed that in the genomes of the several *Chenopodium* diploids under study, the most abundant and the evolutionarily oldest component (*Chenopodium* and *Beta* diverged approximately in the Paleogene) is present. In the remainder of this paper, tandem arrays from the genomes of *Chenopodium* diploid species related to GenBank accession HM641822.1 will be termed the “CficCl-61-40 satDNA family”. This refers to the analysis of NGS data from the *C. ficifolium* genome, RE Cluster #61 (the single cluster in genome of *C. ficifolium* that contains the basic repeat unit), with a length of 40 bp. A further thorough analysis of the interspecies divergence of the sequences of this family was also conducted to identify the main phases of transformation over time.

### 2.2. Sequence Analysis in the CficCl-61-40 satDNA Family

**Figure 2:**
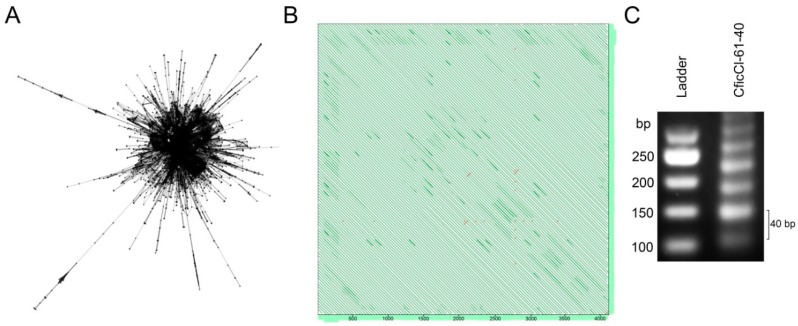
RepeatExplorer (RE) analysis of next-generation sequencing (NGS) data in *Chenopodium* diploids. (**A**) Cluster 61 of *C. ficifolium* demonstrate layouts that are typical for tandem repeats where nodes represent the sequence reads and edges between the nodes correspond to similarity hits; (**B**) Self-to-self comparisons of the contig 25 cluster 61 displayed as dot plots (genomic similarity search tool YASS program output) where parallel lines indicate tandem repeats (the distance between the diagonals equals the lengths of the motifs ~40 bp); (**C**) Agarose gel electrophoresis of PCR products obtained with primers designed from consensus monomer sequence of *C. ficifolium* (Cluster 61) showing typical ladder structure of tandem array.

Among the multitude of clusters produced by RE pipeline, a BLAST search determined a single cluster that belongs to the CficCl-61-40 satDNA family in the genomes of *C. acuminatum*, *C. bryoniifolium*, *C. ficifolium*, *C. iljinii*, *C. pamiricum*, and *C. suecicum* and seven clusters in genome of *C. vulvaria* (supplementary data 1, Figure 2). The highest percentages of the CficCl-61-40 satDNA family were observed in the *C. acuminatum* and *C. bryoniifolium* genomes (Table 2). Subsequent tandem repeat finder (TRF) analysis allows determination of consensus monomer(s) (supplementary data 1). The algorithm of TRF looks for tandem repeats that are often hidden in larger homologous regions or which may fall well below the level of significance required for other programs to report a match. The detection criteria are based on a stochastic model of tandem repeats specified by percent identity and frequency of insertions and deletions rather than some minimal alignment score and align repeat copies against a consensus sequence, revealing patterns of common mutations [30]. Nucleotide sequence divergence among monomers within satDNA arrays is usually quite low, generally, not exceeding a few percent, and for the purpose of sequence analysis, it is acceptable to manipulate with the satDNA consensus sequence [17]. For *C. ficifolium*, *C. pamiricum* and *C. suecicum* a single monomer of ~40 bp was detected. However, for *C. acuminatum*, *C. bryoniifolium*, *C. iljinii*, and *C. vulvaria*, several derivatives from CficCl-61-40 satDNA family monomers were found inside the single cluster. The following two levels of CficCl-61-40 satDNA family variability in the genomes of *C. album* aggregate diploid species were thus observed: (i) at the inter-cluster level, namely single or multiple RE clusters, and (ii) at the intra-cluster level, namely single monomer or a set of related monomers of different lengths detected by TRF.

### 2.3. Reconstruction of the Ancestral Monomer

**Figure 3:**
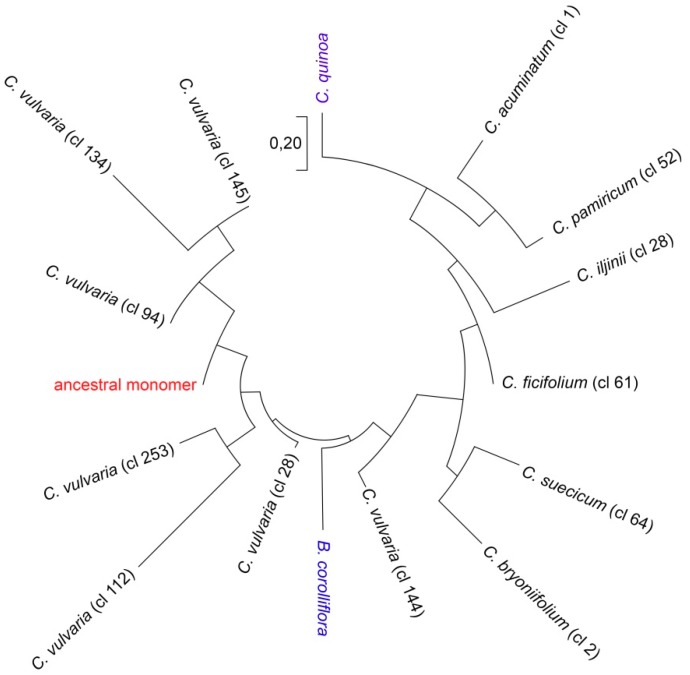
Phylogenetic relationships of the CficCl-61-40 satDNA family sequences. Phylogenetic tree based on the k-mer analysis.

BLAST-detected relatedness between satellite monomers of the CficCl-61-40 satDNA family allowed determination of the major part of the ancestral monomer. For this reconstruction, satellites of *C. bryoniifolium* (consensus monomer from one RE cluster) and *C. vulvaria* (consensus monomers from seven RE clusters) that show relatedness to both *C. quinoa* and *B. corolliflora* satellites were aligned. DNA fragments with 100% BLAST matches in combination formed the most conservative fragment of the basic monomer (supplementary data 2). This approach is quite similar to the method of ancient paralogs (LUCA) [31,32]. The sequence of 37 bp was as follows: TCAAACAAAGCTAATTGAATCAAATGAAAGTCAAATG. This sequence was used as a basis for the subsequent comparison of the monomer divergence in *Chenopodium* lineages. Analysis of basic satellite alterations revealed point mutations, indels, and shifts that were present with different frequencies in the genomes of the studied diploid *Chenopodium* species (supplementary data 1). K-mer based distance estimation revealed a phylogenetically reliable tree with the ancestral monomer as a base, *B. corolliflora* is located separately and rather close to the root, the analyzed diploids that form fairly natural groups with species of clades B and E located nearby, *C. bryoniifolium*, *C. acuminatum* aside, and polyploid *C. quinoa* at the maximum distance from the ancestral monomer (Figure 3).

Clade H (*C. vulvaria*) deserves separate attention. The RE pipeline divided the variety of CficCl-61-40 satDNA family sequences in the genome of *C. vulvaria* into seven clusters (supplementary data 1), indicating valuable heterogeneity. On one hand, all the basic monomers of the clusters contain BLAST-recognizable fragments of the ancestral monomer. On the other hand, the observed variability exceeds that for all clades taken together (Figure 3). An important question is whether all these clusters from the *C. vulvaria* genome belong to the same CficCl-61-40 satDNA family. RE output includes not only the row of clusters but also detailed cluster characteristics, including the cluster neighborhoods of connected components. The analysis showed that all clusters that we classified as belonging to the CficCl-61-40 satDNA family are related to each other and to the repetitive sequences with the GenBank IDs HM641822.1 and AJ288880.1. Additionally, these satDNA clusters possess a limited number of similarity hits with TEs clusters (mainly with Ty3-*gypsy* retrotransposons) which may indicate for splitting of satDNA arrays by the insertion of TEs.

### 2.4. High Order Repeat (HOR) Detection in the CficCl-61-40 satDNA Family and Determination of Its Physical Counterpart 

TRF analysis of the CficCl-61-40 satDNA family in seven diploid species of *Chenopodium* revealed different structures of the arrays. In *C. ficifolium*, *C. pamiricum*, and *C. suecicum*, uniform tandem arrays with basic satellite motifs of ~40 bp (87–96% matches between monomers and copy numbers of 79.2–153.4) were identified by TRF. In *C. acuminatum*, *C. bryoniifolium*, *C. iljinii* and *C. vulvaria*, derivatives from CficCl-61-40 satDNA family repeats ranging up to 332 bp and of different repeatability were found (supplementary data 1). It was proposed that in the latter species, HORs could be formed by concurrent amplification and homogenization of modified monomers.

Here, it is necessary to elucidate the TRF algorithm using an example of the detection of a 117 bp monomer in the genome of *C. acuminatum* (later used as a probe in fluorescent in situ hybridization (FISH) experiments). Analysis of the RE Cluster-1 sequence by TRF produced a table of monomers with the most frequent of 117 bp (consensus size) (supplementary data 1). However, when the consensus sequence was manually analyzed, it decomposed into three 39 bp long subrepeats. Nevertheless, it can be argued that the 117 bp fragment is the basic monomer and that the formation of a HOR unit is based on an ~40 bp monomer. The program finds likely patterns (monomers) and then refines them into a consensus sequence. Patterns are detected by a high percentage of matches at the candidate pattern length. For 39 bp not enough matches were found, but a very high number for 117 bp. This indicates that the unit of duplication was 117 bp and not 39 bp. Furthermore, the mismatches and indels are more consistent with a 117 bp monomer than with a 39 bp monomer (Gary Benson, personal communication). Following sequencing of physical counterparts of CacuCl-1-117 consensus sequence (see below) revealed that the physical components of the CacuCl-1-117 HOR unit did not coincide completely (as in consensus) but varied within the interval of 82% to 86% similarity, which confirmed the accuracy of the TRF algorithm. Additionally, it can be considered that the TRF analysis of all RE clusters belonging to the CficCl-61-40 satDNA family was performed with the same parameters, and in genomes of tree species, only homogeneous arrays were identified while the four other arrays were heterogeneous, which reflects the real structure of satDNA. 

A total of three to four different proposed HOR units were detected in the genomes of *C. acuminatum*, *C. bryoniifolium* and *C. iljinii*. However, approximately 23 such units were found in genome of *C. vulvaria* (supplementary data 1). The genome of *C. vulvaria* is thus again the most variable according to this parameter. 

**Figure 4:**
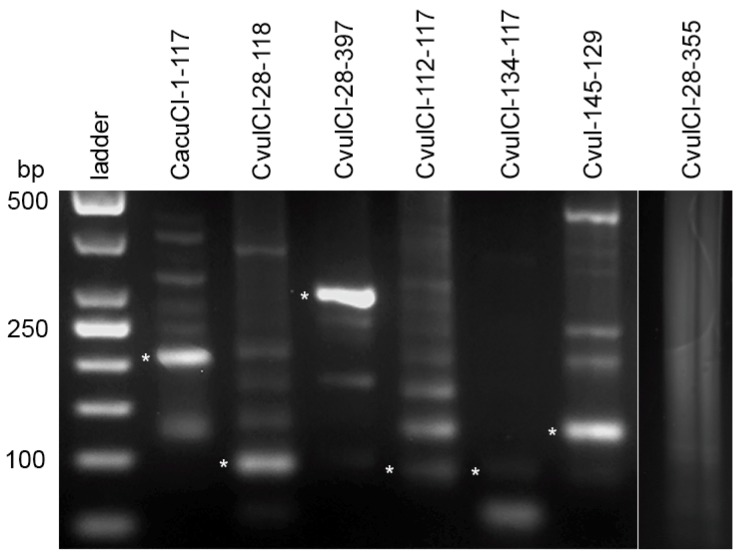
Agarose gel electrophoresis of PCR products obtained with primers designed from consensus monomer sequence of proposed high order repeat (HOR) units for determination of their physical counterparts. Cloned DNA fragments are shown by asterisks. The far-right line is an example of negative amplification of a computer-generated proposed HOR unit.

While there are multiple studies that demonstrate that RE is efficient in repeat identification using NGS, there are some limitations regarding sequence analysis of satellite repeats. The most important one was that generation of consensus sequences by assembling reads to contigs. While this works well for most dispersed repeats like TEs, this is problematic for satellites due to their tandem structure. Consequently, contigs vary in their coverage by reads and their sequences could be partially chimeric (producing sequence variant combinations that in fact do not exist in the genome). To confirm the existence in genomes the physical counterparts of computer-generated consensus monomers we analyzed the sequence variation of CficCl-61-40 and proposed HOR units CacuCl-1-117, CvulCl-28-118, CvulCl-28-397, CvulCl-112-117, CvulCl-134-117 and Cvul-145-129 by cloning. We then compared the obtained sequences with the consensus sequence from the TRF output (supplementary data 3, Figure 4). For all monomers, we obtained several clones that differed from each other as well as from the consensus sequence (supplementary data 4). The CficCl-61-40 monomer is rather uniform with a few point mutations and sequence similarity between clones. The consensus sequence ranged from 90.2% to 95%. For the four obtained clones of the CacuCl-1-117 monomer, two sequence types were found with generally higher similarity to the consensus sequences as well as to each other (similarity value ranges 89.8–91.5 and 90.7–99.2, respectively). This once again confirmed the correctness of the TRF algorithm.

More variability was detected for the proposed HOR units in the *C. vulvaria* genome, which once again highlights the complexity of the satDNA fraction in this species. Thus, among tree clones obtained for the CvulCl-28-118-proposed HOR unit, two sequence types were found with generally high similarity to each other than to the consensus monomer (similarity value ranges 88.2–98.3 and 76.4–79.1, respectively). For CvulCl-28-397-proposed HOR unit sequences amplified by primers (supplementary data 3) also shows more relatedness to each other than to consensus sequence (supplementary data 4). For the CvulCl-112-117- and CvulCl-134-117-proposed HOR units, two types were found among cloned sequences. One showed high relatedness to the consensus monomer (83.3%–90.7%) and the other clones were 100% related to each other and less to the consensus monomer and to the first variant (supplementary data 4). This most likely suggests that several related HOR units could be formed simultaneously. For Cvul-145-129-proposed HOR unit clones possess high similarity to the consensus sequences as well as to each other (82.9%–100.0%). Part of the cloned sequences was submitted to GenBank (accession numbers MH257681–MH257687). However, it should also be noted that we were not able to amplify part of the proposed HOR units generated by TRF analysis (for example CvulCl-28-355 and Cvul-134-148) (supplementary data 1, far right line on Figure 4). These sequences could be attributed most likely to computer-generated chimeric sequences (i.e., method error). However, for the majority of the proposed HOR units its physical counterparts were discovered in the genomes.

### 2.5. Comparison of CficCl-61-40 and Proposed HOR Unit CacuCl-1-117 Chromosomal Distribution

**Figure 5:**
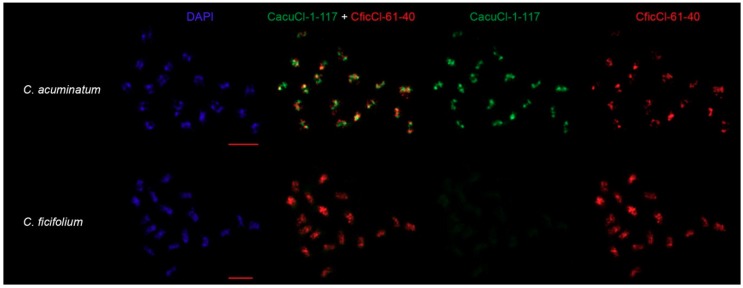
Chromosomal distribution CficCl-61-40 satDNA family sequences. CficCl-61-40 is labelled red; *C. acuminatum*-specific HOR unit CacuCl-1-117 is labelled green. Bar represent 5 μm.

For further confirmation of the existence of HOR units’ in the genomes of *Chenopodium* species an alternative method of FISH was used. Two distant clusters according to phylogenetic analysis, cluster 61 of *C. ficifolium* (CficCl-61-40) and cluster 1 of *C. acuminatum* (CacuCl-1-117) from the RE output, were selected as sources for in situ probes for comparative molecular cytogenetic analysis (Figure 5, supplementary data 1 and 5). FISH experiments were performed to verify if (i) *C. acuminatum*-specific tandem repeats that were proposed to be HOR units (CacuCl-1-117) are species-specific and do not hybridize to chromosomes of the other six species and (ii) if the chromosomal positions of the *C. acuminatum*-specific tandem repeat (CacuCl-1-117) are similar to or different from the positions of the tribe-specific repeat (CficCl-61-40) on the chromosomes of *C. acuminatum*. It should be noted, however, that accurate FISH-based karyotyping and chromosome mapping of CficCl-61-40 satDNA family tandem repeats is challenging in *Chenopodium* due to the small chromosome sizes and to the large number of clusters (Table 2, Figure 5). 

FISH experiments confirmed species specificity and sometimes separate chromosomal positions of newly formed HOR units. Probe CficCl-61-40 hybridized to the chromosomes of all analyzed species except *C. iljinii* (similarity, copy number, or both of the particular FISH probe in *C. iljinii* genome is likely much less in comparison with other species), which demonstrates the presence of a tribe-specific satellite, while CacuCl-1-117 hybridized only to chromosomes of *C. acuminatum* with no signal on the chromosomes of the other six species (Figure 5, supplementary data 5, the minor green signal in *C. pamiricum*, *C. suecicum* and *C. vulvaria* in supplementary data 5 is epifluorescence). In addition, the simultaneous hybridization of CacuCl-1-117 and CficCl-61-40 on the chromosomes of *C. acuminatum* shows that in many cases these tandem arrays form separate clusters that create a species-specific chromosomal pattern (Figure 5).

## 3. Discussion

Application of the RE pipeline for analysis of whole genome shotgun Illumina reads from the genomes of seven diploid *Chenopodium* species from divergent lineages revealed that the investigated CficCl-61-40 satDNA family is the most abundant and oldest component of the *Chenopodium* genome, given that related sequences were found in both *Chenopodium* and *Beta* species. Regarding these two genera, it is essential to note that the genome of *Beta* should be recognized as more static, at least because it contains many fewer species (approximately 7–8 species in total, [33]) in comparison with *Chenopodium* (approximately 150 species [24]). Alignment of the satellite monomers allowed identification of the ancestral DNA fragment of 37 bp that showed 100% identity between *B. corolliflora* from one side and *C. bryoniifolium* and *C. vulvaria* from the other (supplementary data 2). The latter two are species that split off early and possess a modified sequence that is still recognizable by BLAST as a ~40 bp variant of the ancestral monomer. The identified DNA fragment served as a benchmark for our subsequent analyses, in which we intended to characterize intra-unit evolutionary transformations in the diverse *Chenopodium* lineages.

Remarkably, the evolutionary history of the *C. album* aggregate revealed by cpDNA spacers and two low-copy genes [27] correlates fairly well with significant paleoclimatic events. Thus, the early differentiation coincides with the beginning of the Miocene Climatic Optimum in the Burdigalian Age (approximately 20 Mya) (Figure 1). Clade H (*C. vulvaria*) separated upon transition between the Serravallian and Tortonian Ages, ~11 Mya. However, the main lineages were formed in the Pliocene, when due to a cooler and dry, seasonal climate, grasslands spread on all continents, and savannahs and deserts appeared in Asia and Africa. Subsequent speciation within the lineages and the appearance of the majority of polyploids occurred in the Quaternary Period, when the glacial and interglacial epochs succeeded each other. During this time, since there were no places on Earth with identical climate history and since the species of aggregate were spread widely, the CficCl-61-40 satDNA arrays evolved divergently. Excluding clade H, which split off early and is now very different, k-mer-based distance estimation of basic monomer show the most significant differences in genomes of species from clades A and D. It is most likely that both lineages separated early from the ancestral group and evolved independently. This is consistent with the present species distribution ranges and with molecular phylogenetic data [26,27]. However, the pace of evolution of these clades was probably different and is most likely connected with the climatic history of the species distribution areas. In clades B and E, the species are much more similar in the CficCl-61-40 satDNA family structure (Figure 2).

The concept of “molecular drive” [19] postulates that mutations can gradually spread throughout a satDNA family by several of ubiquitous mechanisms of DNA turnover (homogenization) and become fixed in a population. SatDNA families can show a rapid rate of inter-specific evolutionary changes concerning DNA sequence and high levels of conservation between species separated for long evolutionary times [22,34,35]. Although these trends are also true for the CficCl-61-40 satDNA family when monomers are homogenized on the species level in the genomes of different *Chenopodium* lineages, each of them has its own mode and tempo. Although the genome of *C. vulvaria* presents an exception, it seems that concerted evolution does not operate there. This example of non-concerted evolution will be discussed below. 

In addition to mutations in basic satellite monomers, a distinct trend toward increased complexity and length of the monomer (HOR unit formation) was recorded in the species of Clades A, D, E and H of the *C. album* aggregate. HORs occur by concurrent amplification and homogenization of different monomers in the original satDNA when a complex monomer is first formed, after which it merges into a more complex HOR unit [17]. The origin of such structures has been described for the alpha satellite of primates [36], for the satellite families in bovids [37,38] and for the plant species *Vicia grandiflora* [39]. A detailed analysis of the CficCl-61-40 satDNA family tandem arrays in the genomes of *C. acuminatum*, *C. bryoniifolium*, *C. iljinii* and *C. vulvaria* along with the basic ~40 bp monomer revealed related but longer monomers of up to 332 bp, suggesting the generation of new species-specific HOR units. Cloning of PCR-amplified DNA fragments in most cases confirmed the accuracy of the monomer/array compilation produced by the RE pipeline, and the physical counterparts were mostly in agreement with the consensus sequences. However, the exact satDNA array structure of the species could be determined by complete genome sequencing, assembly and annotation [40].

FISH experiments further prove the genesis of species-specific HOR units and their separate locations on the chromosomes. CficCl-61-40 arrays were thus found in all species. On the other hand, related CacuCl-1-117 arrays were found exclusively in *C. acuminatum*, where they form multiple, sometimes separate chromosome clusters, thus creating a species-specific chromosomal pattern (Figure 5). Formation of HOR units based on two or more monomers has been reported in primates and bovids (for a review, see [17]). We observed a similar process but based on the single tribe-specific monomer when unequal changes in the initial sequence in diverging satDNA sets led to monomer alterations with the subsequent merging of the modified monomers in a complex HOR unit. A similar process (i.e., HOR formation based on one initial repeated unit in *Vicia* sp.) was reported by Macas et al. [39]. Presumably, the process of HOR formation on the basis of a single monomer can take more time (in our research, it appears predominantly in ancient species) than that involving two or several monomers, although it apparently contributes to satDNA divergence.

We might next ask whether the formation of HORs is common for plant satDNA evolution. As another example of supposed HOR formation in plants, we can provide a complex structure of the *Hieracium* species centromeric tandem array [41]. Analysis of both RE clusters and the sequenced physical counterparts revealed a complex structure with 21 repetitive elements identified by TRF (ranging from 21 bp to 348 bp) and with two abandoned motifs of 21 and 23 bp. Eventually, we can also observe the stages of HOR formation based on the two short monomers in centromeric regions. It is essential to note that although chromosome segregation machinery is highly conserved across all eukaryotes, centromeric DNA evolves rapidly, and discovered tandem repeats are absent in related *Pilosella* species. Incompatibilities between rapidly evolving centromeric components may be responsible for both the organization of centromeric regions and the reproductive isolation of emerging species [42].

The above examples and the fact that the presented species refer to different large clades of flowering plants suggests that the HOR formation process may not only occur in the *Chenopodium, Hieracium,* and *Vicia* genomes but that this mechanism is also ubiquitous for at least angiosperms and could underlie satDNA divergence in related plant species, as it does in animal genomes. It should also be noted that HOR formation is presumably a species-specific event; in clade B (*C. ficifolium* and *C. suecicum*), neither species showed any sign of HORs. In contrast, in clade E, CficCl-61-40 satDNA family arrays of *C. pamiricum* are uniform, while HORs were detected in the *C. iljinii* genome. However, it is still not clear what triggers the HOR formation in a particular genome [17].

In generalizing the life history of the CficCl-61-40 satDNA family stretching from the ancestral basic repeat unit to species-specific sequences, it is worth noting that the family consists of an extensive group of related, divergent repeats. It is a dominant and old component of *Chenopodium* species genomes and can be characterized by a high complexity of evolution. Independently amplified in each genome, it ultimately acquires lineage-specific profiles due to differential stochastic amplifications, contractions or both. Additionally, in several lineages, a clear trend toward increased complexity and satellite monomer length was observed. Long tandem arrays are characterized by HOR units whose organization and nucleotide sequence are specific for a particular species. Analysis of the sequence organization of these diverged subsets provides a framework for considering mechanisms of sequence diversity generation and for understanding the evolutionary processes of satDNA family homogenization and polymorphism [37]. Homogenization of satellite repeats driven by molecular mechanisms of nonreciprocal sequence transfer occurs simultaneously, which makes satDNA evolve mostly in a concerted manner [3]. Nevertheless, as mentioned above, the small genome of *C. vulvaria* (2C value 0.945 pg) is an exception to this rule. The observed variability indicates a low level of CficCl-61-40 satDNA family homogenization, with multidirectional trends in the *C. vulvaria* genome (non-concerted evolution). Although the data are unusual, our unpublished results on the NGS-based qualitative analysis of TEs in genomes of the same *Chenopodium* diploid species (where we observed that *C. vulvaria* possesses a unique pool of different and diverse retrotransposons [43]) make it possible to hypothesize a link between the TE dynamics and abnormalities in the homogenization of satDNA families, given that satDNA could be a target for TE insertions [44] and evolve further to species-specific tandem repeats [45]. Suppression of concerted evolution resembles those described for termites by Luchetti et al. [46]. This was proposed to be evoked by the limited number of reproducers, especially considering that *C. vulvaria* is an ancient species, restricted to nutrient-rich bare soil largely of anthropogenic impact and not tolerant of competition [47]. Specific habitats may presumably cause abnormal repeatome composition that, in turn, may support the models assuming that genotypes from marginal populations are evolutionarily significant [48,49,50,51]. Despite the causes, discovered suppression of homogenization itself may result in alteration of satDNA libraries, ultimately leading to spontaneous transformation of the entire repeatome, thus producing a novel set of satDNA families for the next round of the conversion cycle, and genomes undergoing non-concerted evolution can be proposed as a significant source of genomic diversity.

## 4. Material and Methods

### 4.1. Plant Material, DNA Extraction, Library Preparation and Illumina Sequencing

For both preparation of the DNA libraries and cytogenetic experiments, plants of the diploid species *C. acuminatum*, *C. bryoniifolium*, *C. ficifolium*, *C. iljinii*, *C. pamiricum*, *C. suecicum* and *C. vulvaria*, which represent the main lineages of the *C. album* aggregate as described in Mandák et al. [27], were used (Table 1). For our research, we sampled genotypes that, according to our previous data, have average parameters for the lineage [27]. All plants were cultivated at the experimental garden of the Institute of Botany, Czech Academy of Sciences, Průhonice, Czech Republic (49.9917° N, 14.5667° E, ca. 320 m above sea level). Leaves were collected, and DNA was extracted using the DNeasy Plant Mini Kit (Qiagen, Venlo, The Netherlands) according to the manufacturer’s instructions. For in situ hybridization experiments, root tips of young, fine roots were collected and fixed as described in Mandák et al. [26] and stored until use. For all analyzed accessions, the ploidy level was verified by flow cytometry as described in Vít et al. [52]. 

One individual per species was used for library preparation and NGS. One microgram of extracted DNA was sheared to fragments of approximately 500 to 600 bp using a Bioruptor Pico sonication device (Diagenode, Liège, Belgium). The NEBNext adaptors for Illumina were ligated to the resulting fragments using the NEBNext Ultra DNA Library Prep Kit for Illumina (New England BioLabs, Ipswich, MA, USA), following the manufacturer’s instructions. The QIAquick PCR Purification Kit (Qiagen) was used to clean the samples from unbound adapters and to concentrate the samples to a total volume of 30 µL. Afterwards, the samples were loaded onto a 1% agarose gel in low EDTA/TAE buffer. Fragments with sizes ranging from 500 to 750 bp were excised and purified using the Zymoclean Gel DNA Recovery Kit (Zymo Research, Irvine, CA, USA) and eluted into 20 µL of ddH2O Concentration was estimated with a Qubit fluorometer using the Qubit HS Assay kit (Thermo Scientific, Waltham, MA, USA). The individual libraries (corresponding to individual species) were enriched and indexed by unique barcodes using PCR with NEBNext Q5 HotStart HiFi PCR Master Mix and NEBNext Multiplex Oligos for Illumina (New England BioLabs) according to the manufacturer’s instructions. The enriched libraries were purified twice using AMPure magnetic beads (Beckman Coulter, Pasadena, CA, USA). The bead:library ratio was 0.7:1 in the first purification and 1:1 in the second purification. The libraries were verified on 1% agarose gels after each purification step. Concentration was measured using the Qubit HS Assay kit (Thermo Scientific) after the final purification step. Libraries of all seven species were pooled and sequenced on an Illumina MiSeq system at Macrogen Inc., to obtain 2*x* 300 bp paired-end reads. 

### 4.2. Clustering of Repeatome Elements 

To process Illumina NGS data and to compare the repetitive DNA fraction of the studied species, a public web server running RE version 1 (http://www.repeatexplorer.org) (České Budějovice, Czech Republic) was used [53]. The discovery and characterization of repetitive elements in the genome was performed using “clustering” tools. An all-to-all sequence comparison of sequencing reads was performed using the mgblast tool. All hits with similarities above 90% over at least 55% of the sequence length were recorded, thus identifying a set of related DNA fragments. The information on similarity hits was used for construction of a graph in which nodes represent sequence reads and the edges between nodes correspond to similarity hits (Figure 2A). This algorithm was first applied to each species separately and subsequently for the seven species in conjunction for the comparative analysis of repeatome quantitative values. For comparative analysis, sampling was performed proportionally to the genome size of the species (Table 2) [26]. 

### 4.3. Satellite DNA Clusters Screening for Tandem Repeats 

All genomically abundant clusters containing at least 0.01% of the input reads were examined manually to select those that potentially possess tandemly organized DNA. Primary selection of the clusters was performed based on their form (Figure 2A). Contigs of each selected cluster were analyzed using the following publicly available online tools: (i) the YASS genomic similarity tool, which enables searches of more fuzzy repeats for potential tandem organization (http://bioinfo.lifl.fr/yass/yass.php) [54], with each contig compared against itself and visualized by dot plots (Figure 2B); (ii) BLAST was used to confirm that the cluster belongs to the CficCl-61-40 satDNA family (supplementary data 1); and (iii) primers were designed from the consensus sequence for PCR conformation of the typical tandem array structure (Figure 2C). Each search was performed for each of the analyzed species.

### 4.4. Sequence Analysis

NGS clusters of *C. acuminatum*, *C. bryoniifolium*, *C. ficifolium*, *C. iljinii*, *C. pamiricum*, *C. suecicum* and *C. vulvaria* falling into the CficCl-61-40 satDNA family were investigated on the intra-unit (analysis of changes in single monomer) and inter-unit (analysis of changes in array components) levels with TRF software (https://tandem.bu.edu/trf/trf.html). As a result, performance tables with data on monomer sizes, copy numbers, percent matches, percent indels and consensus patterns were obtained (supplementary data 1).

For the reconstruction of phylogenetic relationships among the analyzed monomers k-mer based distance estimation was performed [55]. We have chosen the k-mer value equal to 9, as the most optimal for the analyzed sequences. For calculation of distances method based on fractional common k-mer count was used [56]. The phylogenetic relationships among the sequences are then reconstructed from the pairwise distance matrix [57]. The distance matrix thus obtained can be used to construct a phylogenetic tree using the Minimum Evolution method. The construction of the phylogenetic tree was performed in the MEGA program (Figure 3) [58]. The ancestral monomer (root) was reconstructed as follows: nucleotide-BLAST was used to align contigs of each cluster that, according to BLAST searches, show relatedness between satellite monomers of *Chenopodium* and *Beta* species. DNA fragments with 100% similarity were selected and aligned with each other (supplementary data 2). As a result, a fragment of the ancestral monomer was reconstructed.

### 4.5. Detection Physical Counterparts of Basic Monomer and Proposed HOR Units

RE identifies consensus sequences of the most abundant repetitive elements in the genome. However, these consensuses are only virtual assemblies of short reads originating from many different interspersed loci. To reveal the sequences’ physical counterparts and sequence variation within the selected repetitive elements that are proposed to be HOR units, primers were designed based on the consensus sequences (supplementary data 3). PCRs were performed in 25 µL reactions and contained 1× TopBio Plain PP Master Mix (TopBio, Vestec, Czech Republic), each primer at 0.2 mM and 10 to 50 ng of genomic DNA. The cycling conditions were as follows: 4 min at 95 °C followed by 35 cycles of 95 °C for 30 s, sequence-specific annealing temperature for 30 s and 72 °C for 2.5 min, and a final extension at 72 °C for 10 min. The PCR results were verified on a 1% agarose gel (Figure 4). The PCR products of clusters were excised from the gels, cloned and sequenced at GATC Biotech (Konstanz, Germany) according to standard protocols. 

### 4.6. FISH Procedure

FISH analysis was performed to further confirm the physical existence of the HOR units in the genome. Root tips were pre-treated in 0,002 M 8-hydroxyquinolin for 3 h in dark and fixed in 3:1 (*v*/*v*) 100% ethanol:acetic acid. The fixed root meristems were thoroughly washed in water and enzyme buffer (10 mM citrate buffer at pH 4.6) and partially digested in 0,3% (*w*/*v*) cytohelicase, pectolyase and cellulase (Sigma) at 37 °C for 3 h followed by washes in water [27]. The material, in a water drop, was carefully transferred onto a grease-free microscope slide and the cells were spread according to the technique of Pijnacker and Ferwerda [59] with modifications as previously described [60]. 

FISH experiments were performed with clones CficCl-61-40 X-1 and CacuCl-1-117 C-2 as probes labelled with Cy3 (Amersham, Amersham, Buckinghamshire, UK) and biotin (Roche, Basel, Switzerland) according to a standard oligolabeling protocol [61]. For evaluation of probe-specific chromosomal pattern probes were hybridized simultaneously to chromosomes of *C. acuminatum*, *C. bryoniifolium*, *C. ficifolium*, *C. iljinii*, *C. pamiricum*, *C. suecicum* and *C. vulvaria* (Figure 5, supplementary data 5). FISH was performed on ThermoBrite programmable temperature-controlled slide processing system at 63 °C for 3 h. Slides were stained with DAPI and mounted in antifade mountant (Vector Laboratories, Burlingame, CA, USA) and were examined and photographed on Zeiss Axio Imager.Z2 microscope system. Chromosome measurements were obtained by the analysis of metaphase plates using the computer application MicroMeasure version 3.3 [62].

## 5. Conclusions 

Application of the RE pipeline for analysis of whole genome shotgun Illumina reads from the genomes of seven diploid plant species from divergent lineages allowed us to distinguish three types of satDNA family evolutionary development: (i) concerted evolution with mutation and recombination events (most conserved); (ii) concerted evolution with a trend toward increased complexity and length of the satellite monomer (HOR formation); and (iii) non-concerted evolution, with low levels of homogenization and multidirectional trends.

## Figures and Tables

**Table 1:** The accessions and geographic origin of *Chenopodium* diploid species used for satDNA cluster analysis (NGS), probe preparation (cloning) and fluorescent in situ hybridization (FISH).

Species	Clade	ID Number	Locality
*C. acuminatum*	D	429/3	China, Burquin
*C. bryoniifolium*	A	742/4	Russian Federation, Nakhodka
*C. ficifolium*	B	330/2	Czech Republic, Slatina
*C. iljinii*	E	433/9	China, Hoboksar
*C. pamiricum*	E	830/3C	Tajikistan, Gorno-Badakhshan
*C. suecicum*	B	328/10	Czech republic, Švermov
*C. vulvaria*	H	771/1	Iran, Shahr

**Table 2:** Summary of chromosome parameters, genome size, RE clusters and percentage of CficCl-61-40 satDNA family in the genomes of *C. album* aggregate diploid species.

Species	Chr. Numb. 2*n*	Chr. Size μm	Genome Size 2C Values Mb [26]	RE Clusters #	RE Singlets #	CficCl-61-40 % in Genome
*C. acuminatum*	18	0.8–1.5	960	393251	34269	3.80
*C. bryoniifolium*	18	0.7–0.9	1200	307778	38905	2.25
*C. ficifolium*	18	1.5–4.5	1785	369861	20661	0.31
*C. iljinii*	18	1.7–3.3	1144	327760	82679	0.42
*C. pamiricum*	18	1.2–2.5	1154	249599	42427	0.25
*C. suecicum*	18	2.5–5.0	1775	369583	72167	0.27
*C. vulvaria*	18	1.5–2.0	924	542674	93278	0.79

## Supplementary Materials

Supplementary materials can be found at https://www.mdpi.com/1422-0067/20/5/1201/s1. **Supplementary data 1.** Occurrence of CficCl-61-40 satDNA family in genomes of *Chenopodium* diploid species revealed by RepeatExplorer pipeline and formations of high order repeat (HOR) units. **Supplementary data 2.** Reconstruction of the major part of the ancestral monomer. **Supplementary data 3.** Repetitive elements selected for sequence characterization and in situ hybridization and primers used for amplification. **Supplementary data 4.** Pairwise comparison of sequence variation within the CficCl-61-40 (**A**,**B**), and proposed HOR units CacuCl-1-117 (**C**,**D**), CvulCl-28-118 (**E**,**F**), CvulCl-28-397 (**G**,**H**), CvulCl-112-117 (**I**,**J**), CvulCl-134-117 (**K**,**L**) and Cvul-145-129 (**M**,**N**). **Supplementary data 5.** Chromosomal distribution CficCl-61-40 satDNA family sequences. CficCl-61-40 is labelled red; *C. acuminatum*-specific HOR unit CacuCl-1-117 of 117 bp is labelled green.
