# Supplementary material for: Natural History of a Satellite DNA Family: From the Ancestral Genome Component to Species-Specific Sequences, Concerted and Non-Concerted Evolution
Source: Int J Mol Sci. 2019 Mar 9;20(5):1201. doi: 10.3390/ijms20051201 (PMC6429384; doi:10.3390/ijms20051201)

Supplementary Data 1

Occurrence of CficCl-61-40 satDNA family in genomes of *Chenopodium* diploid species revealed by RepeatExplorer pipeline and formations of high order repeat (HOR) units

Species with uniform CficCl-61-40 satDNA family tandem repeat arrays

C. ficifolium

Cluster 61

>CL61Contig25 (4116-54.1-222672)

AATTGAATCAAATGGAATACAAACACATTCAAACAAAGCTAATTGATTCAAATGAAAGTCAAACACATTGAACTAA
AGCTTTATCAATAAAATGAAAGTCAAACACCTTCAAACAAAGTTTTTTGAATTAGAGGAAAGTCAAACACATTTAA
ACAAAGCTTTTTTGAATCAAATGAAAGTCAAACACATTCAAACAAATCTTTTTCAATCAAATGAAAGTCAAATGTAT
TAAAAGAAGCTAAGTGAATCAAATGAAAGTCGAAGACATTTAAACAAAGCTTTTTGAAGCAAAGCAAAGTCAAAC
ACATTCAAACAAAGCTTTTAGAATCAAATGAAAGTCAAACACATTCAAACAAATCTTTTTGAATCAAAAGAAAGTC
AAACATATTCAAACAAAGCTTTTTGAATCAAATGAAAGTCAAACACATTAAAACAAAGCATTTTAAACCAAAAGAA
AATCAAACACATTCAAACAAAGCTTTTTGAATTGAATGAAAGTCAAATGCATTCAACGAAGCTAATTGAATCAAAT
GAAAGTCAAACACATTCAAACAAAGCTTTTTGAATCGAAAGAAAGTCAAACACATTCAAACAAAGCTTTTTGAATC
AAATGAAAGTCAAACACATTCAAACAAAGCTTTTTGAATCAAAAGAAAGTCAAACACATTCAAACAAAGCTTTTTG
AATCAAAATAAAGTCAAACACATTCAAACAAAGCTTTTTGAATCAAATGAAAGTCAAACACATTCAAACAAAGCTT
TTTGAATCAAATGAAAGTCAAACACATTCAAACAAATCTTTTTGAATCAAAATAAAGTCAAACACATTCAAACAAAG
CTTTTTGAATCAAATGAAAGTCAAACACATTCAAACAAAGCTTTTTGAATCAAATGAAAGTCAAACACATTCAAACA
AAGCTTTTTGAATCAAATGAAAGTCAAACACATTCAAACAAAGCTTTTTGAATCAAATGAAAGTCAAACACATTCA
AACAAAGCTTTTTGAATCAAATGAAAGTCAAACACATTCAAACAAAGCTTTTTGAATCAAATGAAAGTCAAACACA
TTCAAACAAAGCTTTTTGAATCAAATGAAAGTCAAACACATTCAAACAAATCTTTTTAAATCAAATGAAAGTCAAAC
ACATTCAAACATAGCTTTTTGAATCAAATGAAAGTCAAACACATTCAAACAAAGCTTTTTGAATCAAATGAAAGTCA
AACACATTCAAACAAAGCTTTTTGAATCAAATGAAAGTCAAACACATTCAAACAATTCTTTTTGAATCAAAATAAAG
TAAAACACATTCAAACAAAGCTTTTTGAATCAAAAGAAAGTCAAACACATTCAAACAAAGCTTTTTGAATCAAATG
AAAGTCAAACACATTCAAACAAAGCTTTTTGAATCAAAAGAAAGTCAAACACATTCAAACAAAGCTTTTTGAATCA
AATGAAAGTCAAACACATTCAAACAAATCTTTTTAAATCAAATGAAAGTCAAACACATTCAAACATAGCTTTTTGAA
TCAAATGAAAGTCAAACACATTCAAACAAAGCTTTTTGAATCAAATGAAAGTCAAACACATTCAAACAAATCTTTTT
AGATCGAAATACAGTCAAACACATTCAAACAAAGCTTTATGAATCAAAAGAAAGTCAAACACATTCAAACAATTCT

TTTTGAATCGAAAAAAAGTAAAAACACATTCAAACAAAGCTTTTTGAATCAAAAGAAAGTCAAACACATTCAAACA
AAGCTTTTTGAATCAAATGAAAGTCAAACACATTCAAACAAAGCTTTTTGAATCAAAATAAAGTCAAAGACATTCA
AACATAGCTTTTTGAATCAAATGAAAGTGAAACACATTCAAACAAAGCTTTTTGAATCAAATAAGAGTCAAACACT
TTCAAACAAATCTCTTTAGATCAAATTACAGTCAAACACATTCAAACAAAGCTTTATGAATCAAAATAAAGTCAAAC
ACATTCTAACAATTCTTTTTAAATCGAAAAAAAATAAAACACATTTAAACAAAGTTTTTTGAATCAAAAGAAAGTCA
AACACATTCAAACAAATCTTTTTGAATCAAATAAAAGTCAAACACATTCAAACAAATATTTTTGAATCATATGAAAG
TCAAACACATTCAAACATAGCTTTTTGAATCAAATGAAAGTCAAACACATTCAAACAAAGCTTTTTGAATCAAATGA
AAGTCAAACACATTCAAACAAATCTTTTTGAATCGAAATAAAGTCAAACACATTCAAACAAAGCTTTTTGAATCAAA
TGAAAGTCAAACACATTCAAACAAAGCTTTTTGAATCAAATGAAAGTCAAACACATTCAAATAAATCTTTTTGAATC
GAAATAAAGTCAAACACATTCAAACAAAGCTTTTTGAATCAAATGAAAGTCAAACACATTCAAACAAAGCTTTTTG
AATCAAATGAAAGTTAAACACATTCAAACAAAGCTTTTTGAATCAAATGAAAGTCAAACACATTCAAACTAACCTTT
TTCGATCGAAATACAGTCAAACACATTCAAAAAAAGCTTAATAAATCAAAAGAAAGTCAAACACATTCAATAAATG
TTTCTGAATAACAAGAAAGTCAAACACATTTAAACAAAGCTTTTTTAATCAAATGAAAGTCAAACATTTAAACAAAT
CTTTTTAAATCAAATGAAAGTCAAACACATACAAACATAGCTTTTTGAATCAAATGAAAGTCAAACACATTCAAACA
ATGCTTTTTGAATTAAATGAAAGACAAAAACATTCAAAAAAATCTTTTTGAATCGAAATAAAGTCTAACACATTCAA
ACAAAGCTTTTTGAATCAAATAAAAGTCAAACACATTCAAACAAATCTTTTTAGATCAAAATACAGTCAAACACATT
CAAACAAATCTTTTTGAATCGAAATAAAGTCAAACACATTAAAACAAAGCTTTTTGAATCAAAAGAAAGTCAAACA
CATTCAAACAAATCTTTTTAAATCAAATGAAAGTCAAACACATTCAAAAAAAGCTTTTTGAATCAAATGAAAATCAA
ATACATTCAAACAATTCTTTTTAAATCGAAAAAAAGTCAAACCCATTCAAACAAAGCCTTTTGAATCAAAATAAAGT
AAAATACATTCAATTATAGCTTTTTGAATCAAATGAAAGTCAAACACATTCAAAAAAATTTTTTTAATCGAAATAA
AGTAAAACAAATTCAAACAAAGCTTTTTGAATTAAAAGAAAGTCAAACACATTCAAATAAAGGGTTTTGAACCAAA
AAAAAGTCAAGCACATTCAAATAAAGCTTTTTAAATCAAAAAAAAAGTAAAACACATACAAACAAATCTTTTTAAAT
CAAATGAAAGTCAAACACATTCAAACAAATCTTTTTGAATCAAATGAAAGTCAAACACATTCAAACAAAGCTTTTTG
AATCAAATGAAAGTCAAAGACATTCATACAAATCTTTTTGAATCAAATAAAAGTCAAACACATTCAAAAAAATCTTT
TTGATTCGAAATAAAGTCAAACACATTCAAACAAAACTTTTTGAATCAAATGAAAGTCAAACACATTCAAGCAAAG
ATTTTTGAATCAAATGAAAGAATAACACATTCAAATAAATCTTTTTTTATCGAAGTAAAGTAAAACACATTCAAACA
AAGCTTTATGAATCAAATGAAAGTTAAACACATTCAACAAAGCATTTTGAATCAAATGAAAGTCAAACACATTCAA
ACAAATCTCTTTAGATCAAAATACAGTCACACACATTCAAACAAAGCTTTATGAATCAAAAGAAAGTCAAACACATT
CTAACAATTCTTTTTGAATCGAAAAAAAGTAAAACACATTCAAACAAAGTTTTTTTAATCAAAAGAAAGTCAAACAC
ATTCAAACAAAGCTTTTTGAATCAAATGAAAGTCAAACACATTCAAACAAATATTTTTAAATCAAATGAAAGTCAAA
CACATTCAAACATAGCTTTTTGAATCAAATGAAAGTCAAACACATTCAAACAAAGCTTTTTGAATCAAATGAAAGTC
AAACACATTCAAACAAAGCTTTTTGAATCAAATGAAAGTTCACATTCACAAAGCTTTTT

Table of detected monomers

Indices	Period Size	Copy Number	Consensus Size	Percent Matches	Percent Indels	Score	A	C	G	T	Entropy (0-2)
3--4096	40	102.4	40	87	3	5732	48	16	8	25	1.75

Consensus pattern (40 bp):

TTGAATCAAATGAAAGTCAAACACATTCAAACAAAGCTTT

BLAST alignments:

Chenopodium quinoa clone 12-13p repeat region sequence. Sequence ID: HM641822.1

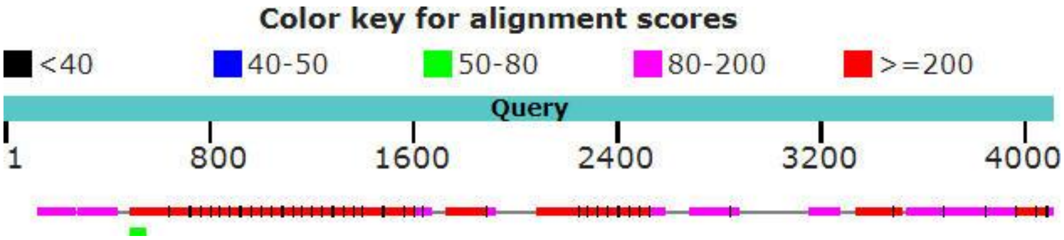

C. pamiricum

CLUSTER 52

>CL52Contig43 (6269-81.2-508851)

AATAAAATGAAAGTCAAACACATTCAAACAAAGCTTTTGGAATCAAATGAAAGTCAAAGACATTCAAACAAAGATT

TTTAGTCAAATGAAAGTCAAACACATTTAAACAAAGCTTTTTGAATCAAATGAAAGTCAAACACATTCAAACAAAG

CTTTTTTAATCAAATGATGTCAAACACATTCAAACAAAGCATTTTGAATCAAATGAAAGTCAAACACATTCAAACAA

AGCTTTTTGAATCAAATGAAAGTCAAACACATTCAAACAAAGCTTTTTGAATCAAATGAAAGTCAAACACATTCAA

ACAAAGCTTTTTGAATCAAATGAAAGTCAAACACATTCAAACAAAGCTTTTGGAATCAAATGAAAGTCAAACACAT

TCAAACAAAGCTTTTTGAATCAAATGAAAGTCAAACACATTCAAACAAAGCTTTTTGAATCAAATGAAAGTCAAAC

ACATTCAAACAAAGCTTTTTGAATCAAATGAAAGTCAAACACATTCAAACAAAGCTTTTTGAATCAAATGAAAGTC

AAACACATTCAAACAAAGCTTTTTGAATCAAATGAAAGTCAAACACATTCAAACAAAGCTTTTTGAATCAAATGAA

AGTCAAACACATTCAAACAAAGCTTTTTGAATCAAATGAAAGTCAAACACATTCAAACAAAGCTTTTTGAATCAAAT

GAAAGTCAAACACATTCAAACAAAGCTTTTTGAATCAAATGAAGTCAAACACATTCAAACAAAGCTTTTTGAATCA

AATGAAAGTCAAACACATTCAAACAAAGCTTTTTGAATCAAATGAAGTCAAACACATTCAAACAAAGCTTATTCAA

TCAAATGAAAGTCAAACACATTCAAACAAAGCTTTTTGAATCAAATGAAAGTCAAACACATTCAAACAAAGCTTTTT

GAATCAAATGAAAGTCAAACACATTCAAACAAAGCTTTTTGAATCAAATGAAAGTCAAACACATTCAAACAAAGCT
TTTTGAATCAAATGAAAGTCAAACACATTCAAACAAAGCTTTTGGAATCAAATGAAGTCAAACACATTCAAACAAA
GCTTTTTGAATCAAATGAAAGTCAAACACATTCAAACAAAGCTTTTTGAATCAAATGAAAGTCAAACACATTCAAAC
AAAGCTTTTTGAATCAAATGAAGTCAAACACATTCAAACAAAGCTTTTTGAATCAAATGAAAGTCAAACACATTCA
AACAAAGCTTTTTGAATCAAATGAAAGTCAAACACATTCAAACAAAGCTTTTTGAATCAAATGAAAGTCAAACACA
TTCAAACAAAGCTTTTTGAATCAAATGAAAGTCAAACACATTCAAACAAAGCTTTTTGAATCAAATGAAAGTCAAA
CACATTCAAACAAAGCTTTTTGAATCAAATGAAAGTCAAACACATTCAAACAAAGCTTTTTGAATCAAATGAAAGTC
AAACACATTCAAACAAAGCTTTTTGAATCAAATGAAAGTCAAACACATTCAAACAAAGCTTTTTGAATCAAATGAA
AGTCAAACACATTCAAACAAAGCTTTTTGAATCAAATGAAAGTCAAACACATTCAAACAAAGCTTTTTGAATCAAAT
GAAAGTCAAACACATTCAAACAAAGCTTTTTGAATCAAATGAAAGTCAAACACATTCAAACAAAGCTTTTTGAATC
AAATGAAAGTCAAACACATTCAAACAAAGCTTTTTGAATCAAATGAAAGTCAAACACATTCAAACAAAGCTTTTTG
AATCAAATGAAAGTCAAACACATTCAAACAAAGCTTTTTGAATCAAATGAAAGTCAAACACATTCAAACAAAGCTT
TTTGAATCAAATGAAAGTCAAACACATTCAAACAAAGCTTTTTGAATCAAATGAAAGTCAAACACATTCAAACAAA
GCTTTTTGAATCAAATGAAAGTCAAACACATTCAAACAAAGCTTTTTGAATCAAATGAAAGTCAAACACATTCAAAC
AAAGCTTTTTGAATCAAATGAAAGTCAAACACATTCAAACAAAGCTTTTTGAATCAAATGAAAGTCAAACACATTC
AAACAAAGCTTTTTGAATCAAATGAAAGTCAAACACATTCAAACAAAGCTTTTTGAATCAAATGAAAGTCAAACAC
ATTCAAACAAAGCTTTTTGAATCAAATGAAAGTCAAACACATTCAAACAAAGCTTTTTGAATCAAATGAAAGTCAA
ACACATTCAAACAAAGCTTTTTGAATCAAATGAAAGTCAAACACATTCAAACAAAGCTTTTTGAATCAAATGAAAG
TCAAACACATTCAAACAAAGCTTTTTGAATCAAATGAAAGTCAAACACATTCAAACAAAGCTTTTTGAATCAAATGA
AAGTCAAACACATTCAAACAAAGCTTTTTGAATCAAATGAAAGTCAAACACATTCAAACAAAGCTTTTTGAATCAA
ATGAAAGTCAAACACATTCAAACAAAGCTTTTTGAATCAAATGAAAGTCAAACACATTCAAACAAAGCTTTTTGAA
TCAAATGAAAGTCAAACACATTCAAACAAAGCTTTTTGAATCAAATGAAAGTCAAACACATTCAAACAAAGCTTTTT
GAATCAAATGAAAGTCAAACACATTCAAACAAAGCTTTTTGAATCAAATGAAAGTCAAACACATTCAAACAAAGCT
TTTTGAATCAAATGAAAGTCAAACACATTCAAACAAAGCTTTTTGAATCAAATGAAAGTCAAACACATTCAAACAA
AGCTTTTTGAATCAAATGAAAGTCAAACACATTCAAACAAAGCTTTTTGAATCAAATGAAAGTCAAACACATTCAA
ACAAAGCTTTTTGAATCAAATGAAAGTCAAACACATTCAAACAAAGCTTTTTGAATCAAATGAAAGTCAAACACAT
TCAAACAAAGCTTTTTGAATCAAATGAAAGTCAAACACATTCAAACAAAGCTTTTTGAATCAAATGAAAGTCAAAC
ACATTCAAACAAAGCTTTTTGAATCAAATGAAAGTCAAACACATTCAAACAAAGCTTTTTGAATCAAATGAAAGTC
AAACACATTCAAACAAAGCTTTTTGAATCAAATGAAAGTCAAACACATTCAAACAAAGCTTTTTGAATCAAATGAA
AGTCAAACACATTCAAACAAAGCTTTTTGAATCAAATGAAAGTCAAACACATTCAAACAAAGCTTTTTGAATCAAAT
GAAAGTCAAACACATTCAAACAAAGCTTTTTGAATCAAATGAAAGTCAAACACATTCAAACAAAGCTTTTTGAATC
AAATGAAAGTCAAACACATTCAAACAAAGCTTTTTGAATCAAATGAAAGTCAAACACATTCAAACAAAGCTTTTTG

AATCAAATGAAAGTCAAACACATTCAAACAAAGCTTTTTGAATCAAATGAAAGTCAAACACATTCAAACAAAGCTT
TTTGAATCAAATGAAAGTCAAACACATTCAAACAAAGCTTTTTGAATCAAATGAAAGTCAAACACATTCAAACAAA
GCTTTTTGAATCAAATGAAAGTCAAACACATTCAAACAAAGCTTTTTGAATCAAATGAAAGTCAAACACATTCAAAC
AAAGCTTTTTGAATCAAATGAAAGTCAAACACATTCAAACAAAGCTTTTTGAATCAAATGAAAGTCAAACACATTC
AAACAAAGCTTTTTGAATCAAATGAAAGTCAAACACATTCAAACAAAGCTTTTTGAATCAAATGAAAGTCAAACAC
ATTCAAACAAAGCTTTTTGAATCAAATGAAAGTCAAACACATTCAAACAAAGCTTTTGGAATCAATGAAAGTTAAA
CACATTCAAACAAAGCTTTTTGAATCAAATGAAAGTTAAACACATTCAATCAAAGCTTTTTGAATCAAATGAAAGTG
AAATGTATTTCACAAAGCTAATTGATACTTTCAAACAAATTTTTTGAATCAAATGAAACTCAAACACATTCAAACAA
AGCTTTTTGAATCAAATGAAAGTCAAACACATTCAAACAAAGCTTTTTGAATCAAATGAAAGTCAAACACATTCAA
ACAAAGCTTTTTGAATCAAATGAAAGTCAAACACATTCAAACAAAGCTTTTTGAATCAAATGAAAGTCAAACACAT
TCAAACAAAGCTTTTTGAATCAAATGAAAGTCAAACACATTCAAACAAAGCTTTTTGAATCAAATGAAAGTCAAAC
ACATTCAAACAAAGCTTTTTGAATCAAATTAAAGTCAAACACATTCAAACAAAGCTTTTTGAATCAAATGAAAGTCA
AACACATTCAAACAAAGCTTTTTGAATCAAATGAAAGTCAAACACATTCAAACAAAGCTTTTTGAATCAAATGAAA
GTCAAACACATTCAAACAAAGCTTTTTGAATCAAATGAAAGTCAAACACATTCAAACAAAGCTTTTTGAATCAAATG
AAAGTCAAACACATTCAAACAAAGCTTTTTGAATCAAATGAAAGTCAAACACATTCAAACAAAGCTTTTTGAATCA
AATGAAAGTCAAACACATTCAAACAAAGCTTTTGGAATCAATGAAAGTTAAACACATTAAACAAAGCTTTTTGAAT
CAAATGAAAGTCAAACACATTCAATCAAAGGTTTTTGAATCAAATGAAAGTCAAACACATTCAAACAAAGCTTTTT
GAATCAAATGAAAGTCAAACACATTCAAACAAAGCTTTTTGAATCAAATGAAAGTCAAACACATTCAAACAAAGCT
TTTTGAATCAAATGAAAGTCAAACACATTCAAACAAAGCTTTTTGAATCAAATGAAAGTCAAACACATTCAAACAA
AGCTTTTGGAATCAATGAAAGTCAAACACATTCAAACAAAGCTTTTTGAATCAAATGAAAGTCAAACACATTCAAT
CAAAGCTTTTTGAATCAAATGAAAGTCAAACACATTCAAACAAAGCTTTTTGAATCAAATGAAAGTCAAACACATTC
AAACAAAGCTTTTTGAATCAAATGAAAGTCAAACACATTCAAACAAAGCTTTTTGAATCAAATGAAAGTCAAACAC
ATTCAAACAAAGCTTTTTGAATCAAATGAAAGTCAAACACATTCAAACAAAGCTTTTTGAATCAAATGAAAGTCAA
ACACATTCAAACAAAGCTTTTTGAATCAAATGAAAGTCAAACACATTCAAACAAAGCTTTTTGAATCAAATGAAAG
TCAAACACATTCAAACAAAGCTTTTTGAATCAAATGAAAGTCAAACACATTCAAACAAAGCTTTTTGAATCAAATGA
AAGTCAAACACATTCAAACAAAGCTTTTTGAATCAAATGAAAGTCAAACACATTCAAACAAAGCTTTTTGAATCAA
ATGAAAGTCAAATGCATTTCACAAAGCTAATTGATACAAAAGAAAGTCAAACACATTCAAACAAAGCTTTTTGAAT
CAAATGAAAGTCAAACACATTCAAACAAAGCTTTTTGAATCAAATGAAAATCAAATGCATTCAACAAAGCTAATTG
AATCAAATGAAAGTCAAACACATTCAAACAAAGCTTTTTGAATCAAATGAAAGTCAAACACATTCAAACAAAGCTT
TTTGAATCAAATGAAAGTCAAACACATTCAAACAAAGCTTTTTGAATCAAATGAAAGTCAAACACATTCAAACAAA
GCTTTTTGAATCAAATGAAAGTCAAATACATTCAAACAAAGCTTTTTGAATCAAATGAAAGTCAAACACATTCAAAC
AAAGCTTTTTGAATCAAATGAAAGTCAAACACATTCAAACAAAGCTTTTTGAATCAAATGAAAGTCAAATGCATTTC

ACAAAGCTAATTGATACAAATGAAAGTTAAACACATTCAAACAAAGCTTTTTGAATCAAATGAAGTCAAATACATT

CAAACAAAGCTTTTTGAATTAAATGAAAGTCAAACACATTCAAACAAAGCTTTTTGAATCAAATGAAAGTCAAACA

CATTCAAACAAAGCTTTTTGAATCAAATGAAAGTCAAACACATTCAAACAAAGCTTTTTGAATCAAATGAAAGTCA

AACACATTCAAACAAAGCTTTTTGAATCAAATGAAAGTCAAATGCATTTCACAAAGCTAATTGATACAAAAGAAAG

TCAAACACATTCAAACAAAGCTTTTTGAATCAAATGAAAGTCAAACTGTTGTAGCCAAGCCTAACAACTTGGTTATG

GACATTTCTTTGGTTCAAGACGTAGCATCATGTATTGGTGCTATGCATTCTATCCATGGGCAACAAGATATTCAATC

CCTTGAGTTTCTTGTT

Table of detected monomers

Indices	Period Size	Copy Number	Consensus Size	Percent Matches	Percent Indels	Score	A	C	G	T	Entropy (0-2)
5--6145	40	153.4	40	96	1	11245	47	17	10	25	1.78

Consensus pattern (40 bp):

AAATGAAAGTCAAACACATTCAAACAAAGCTTTTTGAATC

Chenopodium quinoa clone 12-13p repeat region sequence Sequence ID: HM641822.1

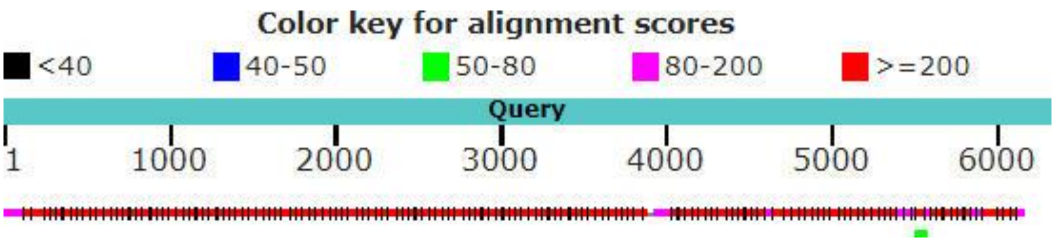

C. suecicum

CLUSTER 64

>CL64Contig159 (3299-17.3-57000)

ATTTTTCTCGAAAAAAGTAAAAACACATTCAAACAATGCTTTTTGAATCAAAAGAAAGTCAAACACATTCAAACAAA

GTATTTTGAATCAAATGAAAGTCAGACACATTGAAAGCAATCTTTTTAATCATATGAAAGTCAAACACATTCAAACA

TAGCTTTTTGAATCAAATGAAAGTCAAACACATTTAAACAAAGCTTTTGAATCAAATGAAAGTCAAACACATTCAAA

CAAATATTTTTAAATCATAAGAAAGTCAAACACATTCAAACATAGCTTTTTGAATCAAATGAAAGTCAAACACATTC

AAACAAAGCTTTTTGAATCAAATGAAAGTCAAACACATTCAAACAAATCTTTTTGAATCGAAATAAAGTCAAACAC
ATTCAAACAAAGCTTTTTGAATCAAATGAAAGTCAAACACATTCAAACAAAGCTTTTTGAATCAAATGAAAGTCAA
ACACATTCAAACAAAGCTTTTTGAATCAAATGAAAGTCAAACACATTCAAACAAATCTTTTTGAATCGAAATAAAGT
CAAACACATTCAAACAAAGCTTTTTGAATCAAATGAAAGTCAAACACATTCAAACAAATCTTTTTGAATCGAAATAA
AAGTCAAACACATTCAAACAAAGCTTTTTGAATCAAATGAAAGTCAAACACATTCAAACAAAGCTTTTTGAATCAA
ATGAAAGTCAAACACATTCAAACAAAGCTTTTTGAATCAAATGAAAGTCAAACACATTCAAATAAATCTTTTTGAAT
CAAATGAAAGTCAAACACATTCAAACAAAGCTTTTTGAATCAAATGAAAGTCAAACACATTCAAACAAAGCTTTTT
GAATCAAATGAAAGTCAAACACATTCAAACAAATCTTTTTAAATCATATGAAAGTCAAACACATTCAAACATAGCTT
TTTAAATCAAATGAAAGTCAAACACATTTAAACAAAGCTTTTAGAATCAAATGAAAGTCAAACACATTCAAATAAAT
CTTTTTGAATTGAAATAAAGTCAAACACATTCAAACAAAGCTTTTTGAATCAAATAAAAGTCAAACACATTCAAACA
AAGCTTTTTGAATCAAATGAAAGTCAAACACATTCAAACAAAGCTTTTTGAATCAAATGAAAGTCAAACACATTTAA
ACAAAGCTTTTTGAATCAAATGAAAGTAAAACACATTCAAACAAGCTTTTTGAATCAAATGAAAGTCAAACACATT
CAAACAAATCTTTCAAAATCAAATGAAAGTCAAACACAAACAAGCATAGCTTTTTGAATCAAATGAAAGTCAAACA
CATTCAAACAAAGCTTTTTGAATCAATTGAAAGTCAAACACATTGAAACAAATCTATTTGGATCGAAATACAGTCAA
ACACATTCAAACATAGCTTTTTGAATCTAATGAATGTCAAATACATTCAAACAAAGCTTTTTGAATCAAATGAAAAT
CAAACACATTCAAATAAATCTTTTTGAATCGAATGAAAGTCAAACACATTCAAACAAAACTTTTTAGATCGAAATTC
AGTCAAACAAATTCAAACAAAGCTTTATGAATCAAAAGAAAGTCAAACACATTCAAAAAAAGCTTTTTGAATCAAA
AAAAGTCAAACACATTCAAACAATTTTATTTGAAACGAAAAAAAGAAAAAACACATTCAAGCAAAGGTTTTTGAAT
CAAAAGAAAGTCAAACACAATTAAACAAAGTTTTATGAATCAAATGAAAGTCAAACACATTTAAACAAATCTTTTTA
AATAAGATGAAAGTCAAACACATTCAAATATAGCATTTTGAATCAAATGAAAGTCAAACACATTTAAACAAAGCTT
TTTGAATCAAATAAAAGTCAAACACTATCAAATTAATCTTTTTGAACAAAATAAAGTCAAACACATTCAAACAATTA
TTTTTAAATCGAAAAAAAGTAAAACACAATAAAACAAAGTTTTCTGAATCAAAAGAAAGTCAAACACATTTAAACA
AAGTTTTTTGATTCAAATGAAAGTCAAACACGTTCAAAAAAATCTTTTTAAATCAAATGAAAGTCAAACACCTTTAA
ACATAGCTTTTTGAATCAAATGAAAGTCAAACACATTCAAACAAAGTATTTTGAATCAAATGAAAGTCAAATACATT
CAAACAAAGCTTTTTGAATCTAATGAAAGTCAAACACATTCAAACAATTCTTTTTGAATCGAAATAAAGTAAAACAA
ATTCAAACAAAGCTTTTTGAATCAAAAGAAAGTCAAACACATTCAAACAAGCTTTTTGAATCAAATGAAAGTCAAA
CACATTCAAACAAATCTTTCAAAATCAAATGAAAGTCAAACACATTCAAACATAGCTTTTTGAATCAAATGAAAGTC
AAACACATTCAAACAAAGCTTTTTGAATCAAATGAAAGTCAAACACGTTCAAACAAAGCTTTTTGAATCAAATGAA
AGTCAAATACATTCAAACAATTCTTTTTGAATCGAAAAAAAGTAAAACACATTAAAACAAAAATTTTTGAATCAAAA
GAAAGTCAAACACATTCAAACAAAGTTTTTTTGAATAAAATGAAAGTCAAACACATTCAAACAAAGCCTTTTAAATC
AAATGAAAGTCAAACACATTCAAACAAAGCTTTTTGAATCAAATGAAAGTCAAACACATTCAAACAATTCTTTTTGA
ATCGAAATAAAGTAAAACAAATTCAAACAAGTTTTTTGAATCAAAAGAAAGTCAAACACATTCAAACAAATCTTTTT

TGAATCAAAAGAAATTAAAACACATTCAAACAAAGCTTTTTGAATCAAAATAAAGTCAAACACATTCAAACATAGC

TTTTGAATCAAATGAAAGTCAAACACATTGTAACAAAGCTTTTTGAATCAAATGAAAGTCAAACACATTCAAACAA

ATCTTTTTAGATCGAAATACAGTCAAACACATTCAAACAAAGCTTTATCAATCAAAATAAAGTCAAACACATTCTAA

CAATTCTGTTTGAATCGAAATAAAGTAAAACACATTCAAACAAGTTTTTTGAATCAAAAGAAAGTCAAACACATTCA

AACAAATCTTTTTAAATCATATGAAAGTCAAACACATTCAAACTTAGTTTTTTTAATCAAATGAAAGTCAAACACATT

TAAACAAAGCTTTTTGAATCAAATGAAAGTCAAACACTGAGAAGGGGGGCACTAGGTCATTGGACTAATCCTCCAT

AAGCCAAACAAAATTCGTAAACACTCAACTAACCTAAATACAAGTATAGAGGTAAGTCAAGGGTCGAGCCCAAAG

GAAC

Table of detected monomers

Indices	Period Size	Copy Number	Consensus Size	Percent Matches	Percent Indels	Score	A	C	G	T	Entropy (0-2)
21--3181	40	79.2	40	87	3	4369	48	16	8	26	1.75

Consensus pattern (40 bp):

AAACACATTCAAACAAAGCTTTTTGAATCAAATGAAAGTC

Chenopodium quinoa clone 12-13p repeat region sequence Sequence ID: HM641822.1

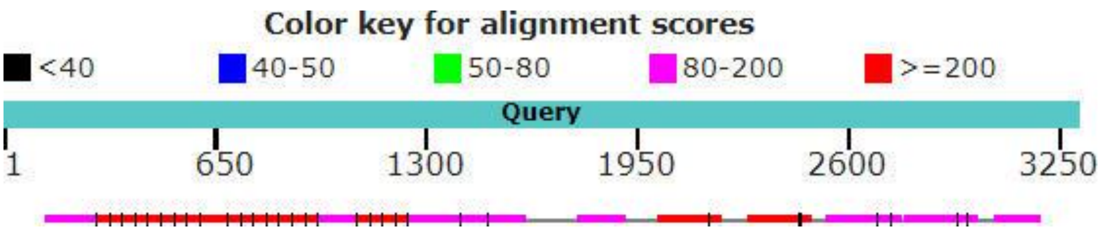

Species with HOR formation on the base of CficCl-61-40 satDNA family tandem repeat arrays

C. acuminatum

CLUSTER 1

>CL1Contig164 (22324-234.7-5239947)

TTTCCCAATTGTTGTATATCGAATTTAAATCTATTAGTTATTGCATATGTCCTGTCACATATCTTGCTTTAATCAGTTA
TATATGGCTTAAGTATGCGGTATTAGCTTTGTTGAATGCAGTTGACATTCATTTGATTCAAAAAGCTTTGTTTGAAT
GTGTTTCACTTTCATTTGATTCTAAAAGCTTTGTTGAATGTGTTTGACTTTCATTTGACTCAATTAGCTTTGTTGAATG
CATTTGACATTCATTTGATTCAAAAACTTTGTTTGAATGTGTTTTACTTTCATTTGATTCTAAAAGCTTTGTTGAAAGT
GTTTGACTTTCATTTGACTCAATTAGCTTTGTTGAATGCATTTGACTTTCATTTGATTCAAAAAGCTTTGTTTGAATG
TGTTTGACTTTCATTTGATTCTAAAAGCTTTGTTGAAAGTGTTTGACTTTCATTTGACTCAATTAGCTTTGTTGAATG
CATTTGACTTTCATTTGAGTCACGAAGCTTTGTTTGAATGTGTTTCACTTTCATTTGATTCTAAAAGCTTTGTTGAAA
GTGTTTGGCTTTCATTTGACTCAATTAGCATTGTGGAATGCAATTAACTTTCATTTGATTCAAAAAGCTTTGTTTGAA
TGTGTTTTACTTTCATTTGATTCTAAAAGCTTTGTTGAAAGTGTTTGACTTTCATTTGACTCAATTAGCTTTGTTGAAT
GCATTTGACTTTCATTTGATTCAAAAAGCTTTGTTTGAATGTGTTTCACTTTCATTTGATTCTAAAAGCTTTGTTGAA
AGTGTTTGACTTTCATTTGACTCAATTAGCTTTGTTGAATGCATTTGACTTTCATTTGAGTCACGAAGCTTTGTTTGA
ATGTGTTTCACTTTCATTTGATTCTAAAAGCTTTGTTGAAAGTGTTTGGCTTTCATTTGACTCAATTAGCATTGTGGA
ATGCAATTAACTTTCATTTGATTCAAAAAGCTTTGTTTGAATGTATTTGACTTTAATTTGATTCTAAAAGCTTTGATG
AAAGTGTTTGACTTTCATTTGACTCAATTAGCTTTGTTGAATGCATTTGACATTCATTTGAATCAAAAATCTTTGTTT
AAATGTGTTTGACGTTCATTTGATTCTAAAAGCTTAGATGAAAGTGTTTGACTTTCATTTGACTCAATTAGCTTTGTT
GAATGCATTTGACATTCATTTGAATCAAAAAGCTTTGTTTAAATGTGTTTGACGTTCATTTGATTCTAAAAGCTTTGA
TGAAAGTGTTTGACTTTCATTTGACTCAATTAGCTTTGTTGAAAGTGTTTGGCTTTCATTTGACTCAATTAGCATTGT
GGAATGCAATTAACTTTCATTTGATTCAAAAAGCTTTGTTTGAATGTATTTGACTTTCATTTGATTCTAAAAGCTTTG
TTGAAAGTGTTTGACTTTCATTTGACTCAATTAGCTTTGTTGAATGCATTTGACATTCATTTGAATCAAAAAGCTTTG
TTTAAATGTGTTTGACGTTCATTTGATTCTAAAAGCTTAGATGAAAGTGTTTGACTTTCATTTGACTCAATTAGCTTT
GTTGAATTCATTTGATTTTCATTTGATTCAAAAAGCTTTGTTTGAATGTGTTTGACTTTCATTTGATTCTAAAAGCTTT
GTTTGAATGTGTTTGACTTTCATTTGATTATAAAAGCTTTGTTGAAAGTGTTTGACTTTCATTTGACTCAATTAGCTT
TGTTGAATGCATTTGACATTCATTTGAATCAAAAAGCTTTGTTTGAATGTGTTTGACTTTCATTTGATTCTAAAAGCT
TTGTTGAAAGTGTTTGACTTTCATTTGACTCAATTAGCTTTGTTGAATGCATTTGACATTCATTTGAATCAAAAAGCT
TTGTTTAAATGTGTTTGACGTTCATTTGATTCTAAAAGCTTTGTTGAAAGTGTTTGACTTTCATTTGACTCAATTAGC
TTTGTTGAATGCATTTGACATTCATTTGAATCAAAAAGCTTTGTTTAAATGTGTTTGACGTTCATTTGATTCTAAAAG
CTTAGATGAAAGTGTTTGACTTTCATTTGACTCAATTAGCTTTGTTGAATTCATTTGATTTTCATTTGATTCAAAAAG

CTTTGTTTGAATGTGTTTGACTTTCATTTGATTCTAAAAGCTTTGTTGAAAGTGTTTGGCTTTCATTTGACTCAATTA
GCTTTGTTGAATGCATTTGACATTCATTTGAATCAAAAAGCTTTGTTTAAATGTGTTTGACGTTCATTTGATTCTAAA
AGCTTTGATGAAAGTGTTTGACTTTCATTTGACTCAATTAGCTTTGTTGAATGCATTTGACATTCATTTGAATCAAAA
AGCTTTGTTTAAATGTGTTTGACGTTCATTTGATTCTAAAAGCTTAGATGAAAGTGTTTGACTTTCATTTGACTCAAT
TAGCTTTGTTGAATTCATTTGATTTTCATTTGATTCAAAAAGCTTTGTTTGAATGTGTTTGACTTTCATTTGATTCTAA
AAGCTTTGTTGAAAGTGTTTGGCTTTCATTTGACTCAATTAGCTTTGTTGAATGCATTTGACATTCATTTGAATCAAA
AAGCTTTGTTTAAATGTGTTTGACGTTCATTTGATTCTAAAAGCTTAGATGAAAGTGTTTGACTTTCATTTGACTCAA
TTAGCTTTGTTGAATGCATTTGACATTCATTTGAATCAAAAAGCTTTGTTTAAATGTGTTTGACGTTCATTTGATTCT
AAAAGCTTAGATGAAAGTGTTTGACTTTCATTTGACTCAATTAGCTTTGTTGAATTCATTTGATTTTCATTTGATTCA
AAAAGCTTTGTTTGAATGTGTTTGACTTTCATTTGATTCTAAAAGCTTTGTTGAAAGTGTTTGACTTTCATTTGACTC
AATTAGCTTTGTTGAATGCATTTGACATTCATTTGAATCAAAAAGCTTTGTTTAAATGTGTTTGACGTTCATTTGATT
CTAAAAGCTTAGATGAAAGTGTTTGACTTTCATTTGACTCAATTAGCTTTGTTGAATTCATTTGATTTTCATTTGATT
CAAAAAGCTTTGTTTGAATGTGTTTGACTTTCATTTGATTCTAAAAGCTTTGTTGAAAGTGTTTGGCTTTCATTTGAC
TCAATTAGCATTGTGGAATGCAATTAACTTTCATTTGATTCAAAAAGCTTTGTTTGAATGTGTTTGACTTTCATTTGA
TTCTAAAAGCTTTGTAGAAAGTGTTTGGCTTTCATTTGACTCAATTAGCATTGTGGAATGCAATTAACTTTCATTTGA
TTCAAAAAGCTTTGATTGAATGTATTTGACTTTCATTTGATTCTAAACGCTTTGTTGCAATGTGTTTGACTTTCATTTA
ATCAAAAAGCTTTGTTTAAATGTGTTTGACGTTCATTTGATTCTAAAAGCTTAGATGAAAGTGTTTGACTTTCATTTG
ACTCAATTAGCTTTGTTGAATTCATTTGATTTTCATTTGATTCAAAAAGCTTTGTTTGAATGTGTTTGACTTTCATTTG
ATTCTAAAAGCTTTGTTGAAAGTGTTTGGCTTTCATTTGACTCAATTAGCATTGTGGAATGCAATTAACTTTCATTTG
ATTCAAAAAGCTTTGATTGAATGTATTTGACTTTCATTTGATTCTAAAAGCTTTGTTGAAAGTGTTTGGCTTTCATTT
GACTCAATTAGCATTGTGGAATGCAATTAACTTTCATTTGATTCAAAAAGCTTTGATTGAATGTATTTGACTTTCATT
TGATTCTAAACGCTTTGTTGCAAGTGTTTGACTTTCATTTGACTCAATTAGCTTTGTTGAATTCATTTGATTTTCATTT
GATTCAAAAAGCTTTGTTTGAATGTGTTTGACTTTCATTTGATTCTAAAAGCTTTGTTGAAAGTGTTTGGCTTTCATT
TGACTCAATTAGCATTGTGGAATGCAATTAACTTTCATTTGATTCAAAAAGCTTTGATTGAATGTATTTGACTTTCAT
TTGATTCTAAACGCTTTGTTGCAAGTGTTTGACTTTCATTTGACTCAATTAGCTTTGTTGAATGCATTTGATTTTCATT
TGATTCAAAAAGCTTTGTTTGAATGTGTTTGACTTTCATTTGATTCTAAAAGCTTTGTTGAAAGTGTTTGGCTTTCAT
TTGACTCAATTAGCATTGTGGAATGCAATTAACTTTCATTTGATTCAAAAAGCTTTGATTGAATGTATTTGACTTTCA
TTTGATTCTAAACGCTTTGTTGCAAGTGTTTGACTTTCATTTGACTCAATTAGCTTTGTTGAATGCATTTGATTTTCAT
TTGATTCAAAAAGCTTTGTTTGAATGTGTTTGACTTTCATTTGATTCTAAAAGCTTTGTTGAAAGTGTTTGGCTTTCA
TTTGACTCAATTAGCATTGTGGAATGCAATTAACTTTCATTTGATTCAAAAAGCTTTGATTGAATGTATTTGACTTTC
ATTTGATTCTAAACGCTTTGTTGCAAGTGTTTGACTTTCATTTGACTCAATTAGCTTTGTTGAATGCATTTGATTTTCA
TTTGATTCAAAAAGCTTTGTTTGAATGTGTTTGACTTTCATTTGATTCTAAAAGCTTTGTTGAAAGTGTTTGGCTTTC

ATTTGACTCAATTAGCATTGTGGAATGCAATTAACTTTCATTTGATTCAAAAAGCTTTGATTGAATGTATTTGACTTT
CATTTGATTCTAAACGCTTTGTTGCAAGTGTTTGACTTTCATTTGACTCAATTAGCTTTGTTGAATGCATTTGATTTTC
ATTTGATTCAAAAAGCTTTGTTTGAATGTGTTTGACTTTCATTTGATTCTAAAAGCTTTGTTGAAAGTGTTTGGCTTC
ATTTGACTCAATTAGCATTGTGGAATGCAATTAACTTTCATTTGATTCAAAAAGCTTTGATTGAATGTATTTGACTTT
CATTTGATTCTAAACGCTTTGTTGCAAGTGTTTGACTTTCATTTGACTCAATTAGCATTGTGGAATGCAATTAACTTT
CATTTGATTCAAAAAGCTTTGTTTGAATGTGTTTGACTTTCATTTGATTCTAAAAGCTTTGTTGAAAGTGTTTGGCTT
TCATTTGACTCAATTAGCATTGTGGAATGCAATTAACTTTCATTTGATTCAAAAAGCTTTGATTGAATGTATTTGACT
TTCATTTGATTCTAAACGCTTTGTTGCAAGTGTTTGACTTTCATTTGACTCAATTAGCTTTGTTGAATGCATTTGATTT
TCATTTGATTCAAAAAGCTTTGTTTGAATGTGTTTCACTTTCATTTGATTCTAAAAGCTTTGTTGAAAGTGTTTGGCT
TTCATTTGACTCAATTAGCATTGTGGAATGCAATTAACTTTCATTTGATTCAAAAAGCTTTGATTGAATGTATTTGAC
TTTCATTTGATTCTAAACGCTTTGTTGCAAGTGTTTGACTTTCATTTGACTCAATTAGCTTTGTTGAATGCATTTGATT
TTCATTTGATTCAAAAAGCTTTGTTTGAATGTGTTTCACTTTCATTTGATTCTAAAAGCTTTGTTGAAAGTGTTTGAC
TTTCATTTGACTCAATTAGCTTTGTTGAATGCATTTGATTTTCATTTGATTCAAAAAGCTTTGTTTGAATGTGTTTCAC
TTTCATTTGATTCTAAAAGCTTTGTTGAAAGTGTTTGACTTTCATTTGACTCAATTATTTGAATGTGTTTGACTTTCAT
TTGATTCTAAAAGCTTTGTTGAAAGTGTTTGGCTTTCATTTGACTCAATTAGCATTGTGGAATGCAATTAACTTTCAT
TTGATTCAAAAAGCTTTGATTGAATGTATTTGACTTTCATTTGATTCTAAACGCTTTGTTGCAAGTGTTTGACTTTCA
TTTGACTCAATTAGCTTTGTTGAATGCATTTGATTTTCATTTGATTCAAAAAGCTTTGTTTGAATGTGTTTCACTTTCA
TTTGATTCTAAAAGCTTTGTTGAAAGTGTTTGACTTTCATTTGACTTAATTAGCTTTGTTGAATGCATTTGACTTTCAT
TTGATTCTAAAAGCTTTGTTGAAAGTGTTTGGCTTTCATTTGACTCAATTAGCATTGTGGAATGCAATTAACTTTCAT
TTGATTCAAAAAGCTTTGATTGAATGTATTTGACTTTCATTTGATTCTAAAAGCTTTGTTGAAAGTGTTTGACTTTCA
TTTGACTCAATTAGCTTTGTTGAATGCATTTGATTTTCATTTGATTCAAAAAGCTTTGTTTGAATGTGTTTCACTTTCA
TTTGATTCTAAAAGCTTTGTTGAAAGTGTTTGACTTTCATTTGACTCAATTAGCATTGTGGAATGCAATTAACTTTCA
TTTGATTCAAAAAGCTTTGATTGAATGTATTTGACTTTCATTTGATTCTAAAAGCTTTGTTGAAAGTGTTTGACTTTC
ATTTGACTCAATTAGCTTTGTTGAATGCATTTGATTTTCATTTGATTCAAAAAGCTTTGTTTGAATGTGTTTCACTTTC
ATTTGATTCTAAAAGCTTTGTTGAAAGTGTTTGACTTTCATTTGACTCAATTAGCTTTGTTGAATGCAATTAACTTTC
ATTTGATTCAAAAAGCTTTGATTGAATGTATTTGACTTTAATTTGATTCTAAAAGCTTTGATGAAAGTGTTTGACTTT
CATTTGACTCAATTAGCTTTGTTGAATTCATTTGATTTTCATTTGATTCAAAAAGCTTTGTTTGAATGTGTTTCACTTT
CATTTGATTCTAAAAGCTTTGTTGAAAGTGTTTGACTTTCATTTGACTTAATTAGCTTTGTTGAATGCAGTTGACATT
CATTTGATTCAAAAAGCTTTGTTTGAATGTGTTTCACTTTCATTTGATTCTAAAAGCTTTGTTGAAAGTGTTTGACTT
TCATTTGACTCAATTAGCTTTGTTGAATGCAATTGACTTTCATTTGATTCAAAAAGCTTTGATTGAATGTATTTGACT
TTCATTTGATTCTAAAAGCTTTGTTGAAAGTGTTTGACTTTCATTTGACTCAATTAGCTTTGTTGAATGCATTTGATTT
TCATTTGATTCAAAAAGCTTTGTTTGAATGTGTTTCACTTTCATTTGATTCTAAAAGCTTTGTTGAAAGTGTTTGACT

TTCATTTGACTCAATTAGCTTTGTTGAATGCAATTGACTTTCATTTGATTCAAAAAGCTTTGTTTGAATGTATTTGAC
TTTCATTTGATTCTAAAAGCTTTGTTGAAAGTGTTTGACTTTCATTTGACTCAATTAGCTTTGTTGAATGCATTTGATT
TTCATTTGATTCAAAAAGCTTTGTTTGAATGTGTTTCACTTTCATTTGATTCTAAAAGCTTTGTTGAAAGTGTTTGAC
TTTCATTTGACTCAATTAGCTTTGTTGAATGCATTTGACTTTCATTTGATTCAAAAAGCTTTGTTTGAATGTGTTTCAC
TTTCATTTGATTCTAAAAGCTTTGTTGAAAGTGTTTGACTTTCATTTGACTTAATTAGCTTTGTTGAATGCAGTTGAC
ATTCATTTGATTCAAAAAGCTTTGTTTGAATGTGTTTGACTTTCATTTGATTCTAAAAGCTTTGTTTGACTCAATTAG
CATTGTGGAATGCAATTAACTTTCATTTGATTCAAAAAGCTTTGATTGAATGTATTTGACTTTCATTTGATTCTAAAA
GCTTTGTTGAAAGTGTTTGACTTTCATTTGACTCAATTAGCTTTGTTGAATGCAATTGACTTTCATTTGATTCAAAAA
GCTTTGATTGAATGTATTTGACTTTCATTTGATTCTAAAAGCTTTGTTGAAAGTGTTTGACTTTCATTTGACTCAATT
AGCTTTGTTGAATGCATTTGATTTTCATTTGATTCAAAAAGCTTTGTTTGAATGTGTTTCACTTTCATTTGATTCTAAA
AGCTTTGTTGAAAGTGTTTGACTTTCATTTGACTTAATTAGCTTTGTTGAATGCAGTTGACATTCATTTGATTCAAAA
AGCTTTGTTTGAATGTGTTTGACTTTAATTTGATTCTAAAAGCTTTGATGAAAGTGTTTGACTTTCATTTGACTCAAT
TAGCTTTGTTGAATTCATTTGATTTTCATTTGATTCAAAAAGCTTTGTTTGAATGTGTTTGACTTTCATTTGATTCTAA
AAGCTTTGTTGAAAGTGTTTGACTTTCATTTGACTCAATTAGCTTTGTTGAATGCATTTGACTTTCATTTGATTCAAA
AAGCTTTGTTTGAATGTGTTTGACTTTCATTTGATTCTAAAAGCTTTGTTGAAAGTGTTTGACTTTCATTTGACTCAA
TTAGCTTTGTTGAATGCATTTGATTTTCATTTGATTCAAAAAGCTTTGTTTGAATGTGTTTCACTTTCATTTGATTCTA
AAAGCTTTGTTGAAAGTGTTTGACTTTCATTTGACTTAATTAGCTTTGTTGAATGCAGTTGACATTCATTTGATTCAA
AAAGCTTTGTTTGAATGTGTTTGACTTTCATTTGATTCTAAAAGCTTTGTTGAAAGTGTTTGACTTTCATTTGACTCA
ATTAGCTTTGTTGAATGCATTTGACTTTCATTTGATTCAAAAAGCTTTGTTTGAATGTGTTTGACTTTCATTTGATTCT
AAAAGCTTTGTTGAAAGTGTTTGACTTTCATTTGACTCAATTAGCTTTGTTGAATGCATTTGATTTTCATTTGATTCA
AAAAGCTTTGTTTGAATGTGTTTCACTTTCATTTGATTCTAAAAGCTTTGTTGAAAGTGTTTGACTTTCATTTGACTT
AATTAGCTTTGTTGAATGCAGTTGACATTCATTTGATTCAAAAAGCTTTGTTTGAATGTGTTTCACTTTCATTTGATT
CTAAAAGCTTTGTTGAAAGTGTTTGACTTTCATTTGACTCAATTAGCTTTGTTGAATGCATTTGACTTTCATTTGATT
CAAAAAGCTTTGTTTGAATGTGTTTGACTTTCATTTGATTCTAAAAGCTTTGATGAAAGTGTTTGACTTTCATTTGAC
TCAATTAGCTTTGTTGAATGCATTTGACTTTCATTTGATTCAAAAAGCTTTGTTTGAATGTGTTTGACTTTCATTTGAT
TCTAAAAGCTTTGATGAAAGTGTTTGACTTTCATTTGACTCAATTAGCTTTGTTGAATGCATTTGATTTTCATTTGAT
TCAAAAAGCTTTGTTTGAATGTGTTTCACTTTCATTTGATTCTAAAAGCTTTGTTGAAAGTGTTTGACTTTCATTTGA
CTTAATTAGCTTTGTTGAATGCAGTTGACATTCATTTGATTCAAAAAGCTTTGTTTGAATGTGTTTCACTTTCATTTG
ATTCTAAAAGCTTTGTTGAAAGTGTTTGACTTTCATTTGACTTAATTAGCTTTGTTGAATGCATTTGACATTCATTTG
ATTCAAAAAGCTTTGTTTGAATGTGTTTGACTTTCATTTGATTCTAAAAGCTTTGTTGAAAGTGTTTGACTTTCATTT
GACTCAATTAGCTTTGTTGAATGCAATTAACTTTCATTTGATTCAAAAAGCTTTGTTTGAATGTATTTGACTTTCATTT
GATTCTAAAAGCTTTGTTGAAAGTGTTTGACTTTCATTTGACTCAATTAGCTTTGTTGAATGCATTTGATTTTCATTT

GATTCAAAAAGCTTTGTTTGAATGTGTTTCACTTTCATTTGATTCTAAAAGCTTTGTTGAAAGTGTTTGACTTTCATT
TGACTCAATTAGCTTTGTTGAATGCAATTGACTTTCATTTGATTCAAAAAGCTTTGATTGAATGTATTTGACTTTCAT
TTGATTCTAAACGCTTTGTTGCAAGTGTTTGACTTTCATTTGACTCAATTAGCTTTGTTGAATGCATTTGATTTTCATT
TGATTCAAAAAGCTTTGTTTGAATGTGTTTCACTTTCATTTGATTCTAAAAGCTTTGTTGAAAGTGTTTGACTTTCAT
TTGACTTAATTAGCTTTGTTGAATGCATTTGACTTTCATTTGATTCAAAAAGCTTTGTTTGAATGTGTTTCACTTTCAT
TTGATTCTAAAAGCTTTGTTGAAAGTGTTTGACTTTCATTTGACTTAATTAGCTTTGTTGAATGCAGTTGACATTCAT
TTGATTCAAAAAGCTTTGTTTGAATGTGTTTCACTTTCATTTGATTCTAAAAGCTTTGTTGAAAGTGTTTGACTTTCA
TTTGACTTAATTAGCTTTGTTGAATGCAGTTGACATTCATTTGATTCAAAAAGCTTTGTTTGAATGTGTTTCACTTTC
ATTTGATTCTAAAAGCTTTGATTGAATGTATTTGACTTTAATTTGATTCTAAAAGCTTTGATGAAAATGGTAGACCTT
CATTTGACTCAATTAGCTTTGTTGAATTCATTTAACTTTCATTTGATTCAAAAAGCTTTGTTTGAATGTATTTGACTTT
CATTTGATTCTAAACGCTTTGTTGAAAGTGTTTGACTTTCATTTGACTCAATTAGCTTTGTTGAATGCATTTGATTTTC
ATTTGATTCAAAAAGCTTTGTTTGAATGTGTTTGACTTTCATTTGATTCTAAAAGCTTTGTTGAAAGTGTTTGACTTT
CATTTGACTCAATTAGCTTTGTTGAATGCATTTGATTTTCATTTGATTCAAAAAGCTTTGTTTGAATGTGTTTCACTTT
CATTTGATTCTAAAAGCTTTGTTGAAAGTGTTTGACTTTCATTTGACTCAATTAGCTTTGTTGAATGCATTTGATTTT
CATTTGATTCAAAAAGCTTTGTTTGAATGTGTTTCACTTTCATTTGATTCTAAAAGCTTTGTTGAAAGTGTTTGACTT
TCATTTGACTTAATTAGCTTTGTTGAATGCAGTTGACATTCATTTGATTCAAAAAGCTTTGTTTGAATGTGTTTGACT
TTCATTTGATTCTAAAAGCTTTGTTGAAAGTGTTTGACTTTCATTTGACTCAATTAGCTTTGTTGAATTCATTTGATTT
TCATTTGATTCAAAAAGCTTTGTTTGAATGTGTTTCACTTTCATTTGATTCTAAAAGCTTTGTTGAAAGTGTTTGACT
TTCATTTGACTTAATTAGCTTTGTTGAATGCATTTGACATTCATTTGATTCAAAAAGCTTTGTTTGAATGTGTTTCACT
TTCATTTGATTCTAAAAGCTTTGTTGAAAGTGTTTGACTTTCATTTGACTTAATTAGCTTTGTTGAATGCAGTTGACA
TTCATTTGATTCAAAAAGCTTTGTTTGAATGTGTTTCACTTTCATTTGATTCTAAAAGCTTTGTTGAAAGTGTTTGAC
TTTCATTTGACTCAATTAGCTTTGTTGAATGCATTTGATTTTCATTTGATTCAAAAAGCTTTGTTTGAATGTGTTTCAC
TTTCATTTGATTCTAAAAGCTTTGTTGAAAGTGTTTGACTTTCATTTGACTTAATTAGCTTTGTTGAATGCAGTTGAC
ATTCATTTGATTCAAAAAGCTTTGTTTGAATGTGTTTCACTTTCATTTGATTCTAAAAGCTTTGTTGAAAGTGTTTGA
CTTTCATTTGACTTAATTAGCTTTGTTGAATGCAGTTGACATTCATTTGATTCAAAAAGCTTTGTTTGAATGTGTTTC
ACTTTCATTTGATTCTAAAAGCTTTGTTGAAAGTGTTTGACTTTCATTTGACTCAATTAGCTTTGTTGAATGCATTTG
ATTTTCATTTGATTCAAAAAGCTTTGTTTGAATGTGTTTCACTTTCATTTGATTCTAAAAGCTTTGTTGAAAGTGTTT
GACTTTCATTTGACTCAATTAGCTTTGTTGAATGCATTTGACATTCATTTGAATCAAAAAGCTTTGTTTAAATGTGTT
TGACCTTCATTTGATTCTAAAAGCTTAGATGAAAGTGGATTGAATGTATTTGACTTTCATTTGATTCTAAACGCTTTG
TTGCAAGTGTTTGACTTTCATTTGACTCAATTAGCTTTGTTGAATGCATTTGATTTTCATTTGATTCAAAAAGCTTTG
TTTGAATGTGTTTGACTTTCATTTGATTCTAAAAGCTTTGTTGAAAGTGTTTGACTTTCATTTGACTCAATTAGCTTT
GTTGAATGCATTTGATTTTCATTTGATTCAAAAAGCTTTGTTTGAATGTGTTTCACTTTCATTTGATTCTAAAAGCTTT

GTTGAAAGTGTTTGACTTTCATTTGACTTAATTAGCTTTGTTGAATGCAGTTGACATTCATTTGATTCAAAAAGCTTT
GTTTGAATGTGTTTCACTTTCATTTGATTCTAAAAGCTTTGTTGAAAGTGTTTGACTTTCATTTGACTCAATTAGCTTT
GTTGAATGCATTTGATTTTCATTTGATTCAAAAAGCTTTGTTTGAATGTGTTTCACTTTCATTTGATTCTAAAAGCTTT
GTTGAAAGTGTTTGACTTTCATTTGACTCAATTAGCTTTGTTGAATGCATTTGACATTCATTTGATTCAAAAAGCTTT
GTTTGAATGTGTTTCACTTTCATTTGATTCTAAAAGCTTTGTTGAAAGTGTTTGACTTTCATTTGACTTAATTAGCTTT
GTTGAATGCAGTTGACATTCATTTGATTCAAAAAGCTTTGTTTGAATGTGTTTCACTTTCATTTGATTCTAAAAGCTT
TGTTGAATGTGTTTGACTTTCATTTGACTCAATTAGCTTTGTTGAATTCATTTGATTTTCATTTGATTCAAAAAGCTTT
GTTTGAATGTGTTTGACTTTCATTTGATTCTAAAAGCTTTGTTGAAAGTGTTTGACTTTCATTTGACTCAATTAGCTT
TGTTGAATGCATTTGATTTTCATTTGATTCAAAAAGCTTTGTTTGAATGTGTTTCACTTTCATTTGATTCTAAAAGCTT
TGTTGAAAGTGTTTGACTTTCATTTGACTTAATTAGCTTTGTTGAATGCAGTTGACATTCATTTGATTCAAAAAGCTT
TGTTTGAATGTGTTTCACTTTCATTTGATTCTAAAAGCTTTGTTGAAAGTGTTTGGCTTTCATTTGACTCAATTAGCTT
TGTTGAATGCATTTAACTTAGGTTTGTTTGAATGTATTTTAATTTCATTTGAGTCACAAAGCTTTGTTTGAATGTGTT
TGACTTTCATTTGACTCAATTAGCTTTGTTGAATTCATTTGATTTTCATTTGATTCAAAAAGCTTTGTTTGAATGTGTT
TGACTTTCATTTGATTCTAAAAGCTTTGATGAAAGTGTTTGACTTTCATTTGACTCAATTAGCTTTGTTGAATTCATTT
GATTTTCATTTGATTCAAAAAGCTTTGTTTGAATGTGTTTCACTTTCATTTGATTCTAAAAGCTTTGTTGAAAGTGTT
TGACTTTCATTTGACTTAATTAGCTTTGTTGAATGCAGTTGACATTCATTTGATTCAAAAAGCTTTGTTTGAATGTGT
TTCACTTTCATTTGATTCTAAAAGCTTTGTTGAAAGTGTTTGACTTTCATTTGACTCAATTAGCATTGTGGAATGCAA
TTGACTTTCATTTGATTCTAAAAGCTTTGATGAAAGTGTTTGACTTTCATTTGACTCAATTAGCTTTGTTGAATTCATT
TGATTTTCATTTGATTCAAAAAGCTTTGTTTGAATGTGTTTCACTTTCATTTGATTCTAAAAGCTTTGTTGAAAGTGT
TTGACTTTCATTTGACTTAATTAGCTTTGTTGAATGCAGTTGACATTCATTTGATTCAAAAAGCTTTGTTTGAATGTG
TTTCACTTTCATTTGATTCTAAAAGCTTTGTTGAAAGTGTTTGACTTTCATTTGACTCAATTAGCTTTGTTGAATGCAT
TTGACATTCATTTGATTCAAAAAGCTTTGTTTGAATGTGTTTCACTTTCATTTGATTCTAAAAGCTTTGTTGAAAGTG
TTTGACTTTCATTTGACTCAATTAGCTTTGTTGAATTCATTTGATTTTCATTTGATTCAAAAAGCTTTGTTTGAATGTG
TTTCACTTTCATTTGATTCTAAAAGCTTTGTTGAAAGTGTTTGACTTTCATTTGACTCAATTAGCTTTGTTGAATGCAT
TTGACTTTCATTTGATTCAAAAAGCTTTGTTTGAATGTGTTTCACTTTCATTTGATTCTAAAAGCTTTGTTGAAAGTG
TTTGACTTTCATTTGACTCAATTAGCTTTGTTGAATGCATTTGATTTTCATTTGATTCAAAAAGCTTTGTTTGAATGTG
TTTCACTTTCATTTGATTCTAAAAGCTTTGTTGAAAGTGTTTGACTTTCATTTGACTTAATTAGCTTTGTTGAATGCA
GTTGACATTCATTTGATTCAAAAAGCTTTGTTTGAATGTGTTTCACTTTCATTTGATTCTAAAAGCTTTGTTGAAAGT
GTTTGACTTTCATTTGACTCAATTAGCTTTGTTGAATGCATTTGACATTCATTTGATTCAAAAAGCTTTGTTTGAATG
TGTTTCACTTTCATTTGATTCTAAAAGCTTTGTTGAAAGTGTTTGACTTTCATTTGACTCAATTAGCTTTGTTGAATTC
ATTTGATTTTCATTTGATTCAAAAAGCTTTGTTTGAATGTGTTTCACTTTCATTTGATTCTAAAAGCTTTGTTGAAAGT
GTTTGACTTTCATTTGACTCAATTAGCTTTGTTGAATGCATTTGATTTTCATTTGATTCAAAAAGCTTTGTTTGAATGT

GTTTCACTTTCATTTGATTCTAAAAGCTTTGTTGAAAGTGTTTGACTTTCATTTGACTTAATTAGCTTTGTTGAATGC
AGTTGACATTCATTTGATTCAAAAAGCTTTGTTTGAATGTGTTTCACTTTCATTTGATTCTAAAAGCTTTGTTGAAAG
TGTTTGGCTTTCATTTGACTCAATTAGCATTGTGGAATGCAATTAACTTTCATTTGATTCAAAAAGCTTTGTTTGAAT
GTGTTTCACTTTCATTTGATTCTAAAAGCTTTGTTGAAAGTGTTTGACTTTCATTTGACTCAATTAGCTTTGTTGAAT
GCATTTGACTTTCATTTGAATCAAAAAGCTTTGTTTGAATGTGTTTGACTTTCTTTTGACTCAATTAGCTTTGTTGAA
TGTGTTTGACTTTCATTTGACTTTTTGTTGAAAGTGTTTGACTTTCATTTGACTCAATTAGCTTTGTTGAATTCATTTG
ATTTTCATTTGATTCAAAAAGCTTTGTTTGAATGTGTTTGACTTTCATTTGATTCTAAAAGCTTTGTTGAAAGTGTTT
GACTTTCATTTGACTCAATTAGCTTTGTTGAATGCATTTGACATTCATTTGATTCAAAAAGCTTTGTTTGAATGTGTT
TCACTTTCATTTGATTCTAAAAGCTTTGTTGAAAGTGTTTGACTTTCATTTGACTCAATTAGCTTTGTTGAATGCATTT
GACTTTCATTTGATTCAAAAAGCTTTGTTTGAATGTGTTTCACTTTCATTTGATTCTAAAAGCTTTGTTGAAAGTGTT
TGACTTTCATTTGACTTAATTAGCTTTGTTGAATGCAGTTGACATTCATTTGATTCAAAAAGCTTTGTTTGAATGTGT
TTCACTTTCATTTGATTCTAAAAGCTTTGTTGAAAGTGTTTGACTTTCATTTGACTCAATTAGCTTTGTTGAATGCATT
TGACTTTCATTTGATTCAAAAAGCTTTGTTTGAATGTGTTTCACTTTCATTTGATTCTAAAAGCTTTGTTGAAAGTGT
TTGACTTTCATTTGACTTAATTAGCTTTGTTGAATGCAGTTGACATTCATTTGATTCAAAAAGCTTTGTTTGAATGTG
TTTCACTTTCATTTGATTCTAAAAGCTTTGTTGAAAGTGTTTGGCTTTCATTTGACTCAATTAGCATTGTGGAATGCA
ATTAACTTTCATTTGATTCAAAAAGCTTTCATTGACTTTCATTTGATTTAAAAAGCATTGTTTGAAAGTGTTTGACTTT
CATTTGACTCAATTAGCTTTGTTGAATTCATTTGATTTTCATTTGATTCAAAAAGCTTTGTTTGAATGTGTTTCACTTT
CATTTGATTCTAAAAGCTTTGTTGAAAGTGTTTGACTTTCATTTGACTCAATTAGCTTTGTTGAATGCATTTGACATT
CATTTGATTCAAAAAGCTTTGTTTGAATGTGTTTCACTTTCATTTGATTCTAAAAGCTTTGTTGAAAGTGTTTGACTT
TCATTTGACTTAATTAGCTTTGTTGAATGCAGTTGACATTCATTTGATTCAAAAAGCTTTGTTTGAATGTGTTTCACT
TTCATTTGATTCTAAAAGCTTTGTTGAATGTGTTTGACTTTCATTTGACTCAATTAGCTTTGTTGAATGCATTTGACA
TTCATTTGATTCAAAAAGCTTTGTTTGAATGTGTTTCACTTTCATTTGATTCTAAAAGCTTTGTTGAAAGTGTTTGAC
TTTCATTTGACTCAATTAGCTTTGTTGAATGCAGTTGACATTCATTTGATTCAAAAAGCTTTGTTTGAATGTGTTTCA
CTTTCATTTGATTCTAAAAGCTTTGTTGAAAGTGTTTGGCTTTCATTTGACTCAATTAGCATTGTGGAATGCAATTAA
CTTTCATTTGATTCAAAAAGCTTTGTTTGAATTAGCTTTGTTGAATTCATTTGATTTTCATTTGATTCAAAAAGCTTTG
TTTGAATGTGTTTCACTTTCATTTGATTCTAAAAGCTTTGTTGAAAGTGTTTGACTTTCATTTGACTCAATTAGCTTTG
TTGAATGCATTTGATTTTCATTTGATTCAAAAAGCTTTGTTTGAATGTGTTTCACTTTCATTTGATTCTAAAAGCTTTG
TTGAAAGTGTTTGACTTTCATTTGACTTAATTAGCTTTGTTGAATGCAGTTGACATTCATTTGATTCAAAAAGCTTTG
TTTGAATGTGTTTCACTTTCATTTGATTCTAAAAGCTTTGTTGAAAGTGTTTGACTTTCATTTGACTCAATTAGCTTTG
TTGAATGCATTTGACATTCATTTGATTCAAAAAGCTTTGTTTGAATGTGTTTCACTTTCATTTGATTCTAAAAGCTTT
GTTGAAAGTGTTTGGCTTTCATTTGACTCAATTAGCTTTGTTGAATGCAATTGACTTTCATTTGATTCAAAAAGCTTT
GTTTGAATGTGTTTCACTTTCATTTGATTCTAAAAGCTTTGTTGAAAGTGTTTGACTTTCATTTGACTTAATTAGCTTT

GTTGAATGCAGTTGACATTCATTTGATTCAAAAAGCTTTGTTTGAATGTGTTTCACTTTCATTTGATTCTAAAAGCTT
TGTTGAAAGTGTTTGACTTTCATTTGACTCAATTAGCATTGTGGAATGCAATTAACTTTCATTTGATTCAAAAAGCTT
TGTTTGAATGTATTTGACTTTCATTTGACTCAATTGGCTTTGTTGAATGCATTTGACTTTCATTTGACTCAATTAGTTT
TCATTTGACTCAATTTGGCTTTCATTCGACTCAATTAGCTTTGTTGAATTCATTTGATTTTCATTTGATTCAAAAAGCT
TTGTTTGAATGTGTTTCACTTTCATTTGATTCTAAAAGCTTTGTTGAAAGTGTTTGACTTTCATTTGACTTAATTAGCT
TTGTTGAATGCATTTGATTTTCATTTGATTCAAAAAGCTTTGTTTGAATGTGTTTCACTTTCATTTGATTCTAAAAGCT
TTGTTGAAAGTGTTTGACTTTCATTTGACTTAATTAGCTTTGTTGAATGCAGTTGACATTCATTTGATTCAAAAAGCT
TTGTTTGAATGTGTTTCACTTTCATTTGATTCTAAAAGCTTTGTTGAAAGTGTTTGACTTTCATTTGACTCAATTAGCT
TTGTTGAATGCAATTGACTTTCATTTGATTCAAAAAGCTTTGTTTGAATGTGTTTCACTTTCATTTGATTCTAAAAGC
TTTGTTGAAAGTGTTTGACTTTCATTTGACTTAATTAGCTTTGTTGAATGCAGTTGACATTCATTTGATTCAAAAAGC
TTTGTTTGAATGTGTTTCACTTTCATTTGATTCTAAAAGCTTTGTTGAAAGTGTTTGGCTTTCATTTGACTCAATTAGC
ATTGTGGAATGCAATTAACTTTCATTTGATTCAAAAAGCTTTGTTTGAATGTATTTGACTTTCATTTGATTCTAAAAG
CTTTGTTGAAAGTGTTTGACTTTCATTTGACTTAATTAGCTTTGTTGAATGCAGTTGACATTCATTTGATTCAAAAAG
CTTTGTTTGAATGTGTTTCACTTTCATTTGATTCTAAAAGCTTTGTTGAAAGTGTTTGGCTTTCATTTGACTCAATTAG
CATTGTGGAATGCAATTAACTTTCATTTGATTCAAAAAGCTTTGTTTGAATGTATTTGACTTTAATTTGATTCTAAAA
GCTTTGATGAAAGTGTTTGACTTTCATTTGACTCAATTAGCTTTGTTGAATTCATTTGATTTTCATTTGATTCTAAAA
GCTTTGTTGAAAGTGTTTGACTTTCATTTGACTTAATTAGCTTTGTTGAATGCAGTTGACATTCATTTGATTCAAAAA
GCTTTGTTTGAATGTGTTTCACTTTCATTTGATTCTAAAAGCTTTGTTGAAAGTGTTTGGCTTTCATTTGACTCAATTA
GCATTGTGGAATGCAATTAACTTTCATTTGATTCAAAAAGCTTTGTTTGAATGTATTTGACTTTAATTTGATTCTAAA
AGCTTTGATGAAAGTGTTTGACTTTCATTTGACTCAATTAGCTTTGTTGAATTCATTTTCACTTTCATTTGATTCTAAA
AGCTTTGTTGAAAGTGTTTGACTTTCATTTGACTTAATTAGCTTTGTTGAATGCAGTTGACATTCATTTGATTCAAAA
AGCTTTGTTTGAATGTGTTTCACTTTCATTTGATTCTAAAAGCTTTGTTGAAAGTGTTTGGCTTTCATTTGACTCAATT
AGCATTGTGGAATGCAATTAACTTTCATTTGATTCAAAAAGCTTTGTTTGAATGTATTTGACTTTAATTTGATTCTAA
AAGCTTTGTTGAAAGTGTTTGACTTTCATTTGACTTAATTAGCTTTGTTGAATGCAGTTGACATTCATTTGATTCAAA
AAGCTTTGTTTGAATGTGTTTCACTTTCATTTGATTCTAAAAGCTTTGTTGAAAGTGTTTGGCTTTCATTTGACTCAA
TTAGCATTGTGGAATGCAATTAACTTTCATTTGATTCAAAAAGCTTTGTTTGAATGTATTTGACTTTAATTTGATTCT
AAAAGCTTTGATGAAAGTGTTTGACTTTCATTTGACTCAATTAGCTTTGTTGAATTCATTTGATTTTCATTTGATTTTC
ATTTGACTTAATTAGCTTTGTTGAATGCAGTTGACATTCATTTGATTCAAAAAGCTTTGTTTGAATGTGTTTCACTTT
CATTTGATTCTAAAAGCTTTGTTGAAAGTGTTTGGCTTTCATTTGACTCAATTAGCATTGTGGAATGCAATTAACTTT
CATTTGATTCAAAAAGCTTTGTTTGAATGTATTTGACTTTAATTTGATTGTAAAACCTTTGATGAAAGTGTTTGACTT
TCATTTGACTCAATTAGCTTTGTTGAATTCATTTGATTTTCATTTGATTCTTGCAAGTGTTTGTTTGACTTTCATTTGA
CTTAATTAGCTTTGTTGAATGCAGTTGACATTCATTTGATTCAAAAAGCTTTGTTTGAATGTGTTTCACTTTCATTTG

ATTCTAAAAGCTTTGTTGAAAGTGTTTGGCTTTCATTTGACTCAATTAGCATTGTGGAATGCAATTAACTTTCATTTG
ATTCAAAAAGCTTTGTTTGAATGTATTTGACTTTAATTTGATTCTAAAAGCTTTGATGAAAGTGTTTGACTTTCATTT
GACTCAATTAGCTTTGTTGAATGCATTTGACTTTCATTTGATTCAAAAAGCTTTGTTTGAATGTATTTGACTTTAATT
TGATTCTAAAAGCTTTGATGAAAGTGTTTGACTTTCATTTGACTCAATTAGCTTTGTTGAATTCATTTGATTTTCATTT
GATTCAAAAAGCTTTGTTTGAATGTGTTTCACTTTCATTTGATTCTAAAAGCTTTGATGAAAGTGTTTGACTTTCATT
TGACTCAATTAGCTTTGTTGAATTCATTTGATTTTCATTTGATTCAAAAAGCTTTGTTTGAATGTGTTTCACTTTCATT
TGATTCTAAATCATTTGACTCAATTAGCTTTGTTGAATGCATTTTGACATTCATTTGATTCAAAAAGCTTTGTTTGAA
TGTGTTTCACTTTCATTTGATTCTAAAAGCTTTGTTGAAAGTGTTTGGCTTTCATTTGACTCAATTAGCATTGTGGAA
TGCAATTAACTTTCATTTGATTCAAAAAGCTTTGTTTGAATGTATTTGACTTTAATTTGATTCTAAAAGCTTTGATGA
AAGTGTTTGACTTTCATTTGACTCAATTAGCTTTGTTGAATTCATTTGATTTTCATTTGATTCAAAAAGCTTTGTTTGA
ATGTGTTTCACTTTCATTTGATTCTAAAAGCTTTGTTGAAAGTGTTTGGCTTTCATTTGACTCAATTAGCATTGTGGA
ATGCAATTAACTTTCATTTGATTCAAAAAGCTTTGTTTGAATGTCTTTGACTTTAATTTGATTCTAAAAGCTTTGATG
AAAGTGTTTGACTTTCATTTGACTCAATTAGCTTTGTTGAATTCATTTGATTTTCATTTGATTCAAAAAGCTTTGTTTG
AATGTGTTTCACTTTCATTTGATTCTAAAAGCTTTGTTGAAAGTGTTTGACTTTCATTTGACTCAATTAGCTTTGTTG
AATTCATTTGATTTTCATTTGATTCAAAAAGCTTTGTTTGAATGTGTTTCACTTTCATTTGATTCTAAAAGCTTTGTTG
AAAGTGTTTGACTTTCAATTAAAAAACCTTTGTTTGAATGTGTTTGACTTTCATTTGATTCTAAAAGCTTTGTTGAAA
GTGTTTGGCTTTCATTTGACTCAATTAGCATTGTGGAATGCAATTAACTTTCATTTGATTCAAAAAGCTTTGTTTGAA
TGTGTTTCACTTTCATTTGATTCTAAAAGCTTTGTTGAAAGTGTTTGGCTTTCATTTGACTCAATTAGCATTGTGGAA
TGCAATTAACTTTCATTTGATTCAAAAAGCTTTGTTTGAATGTATTTGACTTTAATTTGATTCTAAAAGCTTTGATGA
AAGTGTTTGACTTTCATTTGACTCAATTAGCTTTGTTGAATTCATTTGATTTTCATTTGATTCAAAAAGCTTTGTTTGA
ATGTGTTTCACTTTCATTTGATTCTAAAAGCTTTGTTGAAAGTGTTTGACTTTCATTTGACTCAATTAGCATTGTGGA
ATGCAATTAACTTTCATTTGATTCAAAAAGCTTTGTTTGAATGTATTTGACTTTAATTTGATTCTAAAAGCTTTGATG
AAAGTGTTTGACTTTCATTTGACTCAATTAGCTTTGTTGAATTCATTTGATTTTCATTTGATTCAAAAAGCTTTGTTTG
AATGTGTTTCACTTTCATTTGATTCTAAAAGCTTTGTTGAAAGTGTTTGACTTTCATTTGACTCAATTAGCTTTGTTG
AATGCATTTGACATTCATTTGAATCAAAAAGCTTTGTTTAAATGTGTTTGACGTTCATTTGATTCTAAAAGCTTAGAT
GAAAGTGTTTGACTTTCATTTGACTCAATTAGCTTTATTGAATGCATTTGACATTCATTTGAATCAAAAAGCTTTGTT
TAAATGTGTTTGACGTTCATTTGATTCTAAAAGCTTAGATGAAAGTGTTTGACTTTCATTTGATTCTAAAAGCTTTGT
TGAAAGTGTTTGGCTTTCATTTGACTCAATTAGCATTGTGGAATGCAATTAACTTTCATTTGATTCAAAAAGCTTTGC
TTGAATGTATTTGACTTTAATTTGATTCTAAAAGCTTTGATGAAAGTGTTTGACTTTCATTTGACTCAATTAGCTTTG
TTGAATGCATTTGATTTTCATTTGATTCAAAAAGCTTTGTTTGAATGTGTTTGACTTTCATTTGACTCAATTAGCTTTG
TTGAATTCATTTGATTTTCATTTGATTCAAAAAGCTTTGTTTGAATGTGTTTGACTTTCATTTGATTCTAAAAGCTTTG
TTGAAAGTGTTTGGCTTTCATTTGACTCAATTAGCATTGTGGAATGCAATTAACTTTCATTTGATTCAAAAAGCTTTG

ATTGAATGTATTTGACTTTAATTTGATTCTAAAAGCTTTGATGAAAGTGTTTGACTTTCATTTGACTCAATCAGCTTT
GTTGAATGCATTTGACATTCATTTGAATCAAAAATCTTTGTTTGAATTTTTTTCACTTTCATTTGATTCTAACAGATTT
TTTGT

Table of detected monomers

Indices	Period Size	Copy Number	Consensus Size	Percent Matches	Percent Indels	Score	A	C	G	T	Entropy (0-2)
104--22309	118	188.0	117	90	3	23488	25	11	16	45	1.81
7481--7678	98	2.0	98	92	0	324	27	11	16	44	1.82
12915--13113	104	1.9	103	89	3	310	22	11	17	49	1.78
15025--15087	32	2.0	32	96	0	117	17	9	19	53	1.70
16539--16567	14	2.0	15	93	6	51	20	6	20	51	1.70
17308--17372	21	3.2	21	68	12	64	21	12	16	49	1.79
17244--17426	20	9.2	20	67	12	99	24	15	14	45	1.84
19242--19272	11	2.8	11	100	0	62	19	9	9	61	1.54
19207--19313	50	2.1	50	89	0	160	22	12	14	51	1.74
20083--20188	55	1.9	54	83	7	133	25	14	13	47	1.80
20044--20232	95	2.0	94	95	1	342	25	13	13	47	1.79
20307--22105	39	45.9	37	68	15	257	26	11	16	45	1.81

Consensus pattern (117 bp):

AGCTTTGTTGAATGTGTTTGACTTTCATTTGATTCAAAAAGCTTTGTTGAATGTGTTTGACTTTCATTTGATTCAAAA
AGCTTTGTTGAATGTGTTTGACTTTCATTTGATTCAAAA

Consensus pattern (98 bp):

TTTGACTCAATTAGCATTGTGGAATGCAATTAACATTCATTTGATTCAAAAAGCTTTGATTGAATGTATTTGACTTTC
ATTTGATTCTAAAAGCTTTG

Consensus pattern (103 bp):

TTTGTTTGAATGTATTTCAATTTCATTTGAGTCTAAAAGCTTTGTTGAAAGTGTTTGACTTTCATTTGACTCAATTAG
CTTTGTTGAATGCATTTAACTTAGG

Consensus pattern (32 bp):

TTTGTTGAAAGTGTTTGACTTTCATTTGACTT

Consensus pattern (15 bp):

AGCTTTGTTTGAATT

Consensus pattern (21 bp):

TTTGAATGCATTTGACTTTCA

Consensus pattern (20 bp):

CTTTCATTTGACTCAATTAG

Consensus pattern (11 bp):

TTCATTTGATT

Consensus pattern (50 bp):

TTTGATTTTCATTTGACTCAATTAGCTTTGTTGAATGCAGTTGACATTCA

Consensus pattern (54 bp):

TCATTTGACTCAAAAAGCTTTGTTGAATGATTTTCACATTCATTTGATTCTAAA

Consensus pattern (94 bp):

TCATTTGACTCAATTAGCTTTGTTGAATGCATTTGACATTCATTTGATTCAAAAAGCTTTGTTTG

AATGTGTTTCACTTTCATTTGATTCTAAA

Consensus pattern (37 bp):

AAAAAGCTTTGTTTGAATGTGTTTGACTTTCATATTA

BLAST alignments:

Chenopodium quinoa clone 12-13p repeat region sequence Sequence ID: HM641822.1

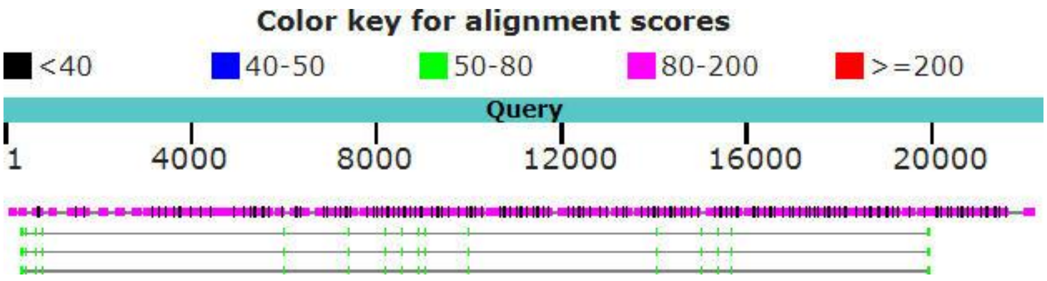

C. bryonifolium

CLUSTER 2

>CL2Contig17 (6990-40.3-281419)

TTTGATCGTGTTTGACTTTCATTTGATTCAAAAAGCTTTGTTTGTATGTGTTTGACTTTCATTTGTTTCAATTAGCTTT

GTAGAATGCATTTGACTTTCATTTGATTCAAACAGCTTTGTTTGAATGTGTTTGACTTTCATTTGATTCAAAAAGCTT

TGTTTGAATGTGTTTGACTTTCATTTGATTCAATTAGCTTTGTAGAATGCATTTGACTTTCATTTGATTCAAATAGCTT

TGTTTGAATGTGTTTGACTTTCATTTGATTCAAAAAGCTTTGTTTGAATGTGTTTGACTTTCATTTGATTCAATTAGCT
TTGTAGAATGCATTTGACTTTCATTTGATTCAAATAGCTTTGTTTGAATGTGTTTGACTTTCATTTGATTCAAATAGC
TTTATTTGAATGTGTTTGACTTTCATTTGATTCAAATAGCTTTGTTTGAATGTGTTTGACTTTCATTTGATTCAATTAG
CTTTGTAGAATGCATTTGACTTTCATTTGATTCAAATAGCTTTGTTTGAATGTGTTTGACTTTCATTTGATTCAATTAG
CTTTGTAGAATGCATTTGACTTTCATTTGATTCAAATAGCTTTGTTTGAATGTGTTTGACTTTCATTTGATTCAAAAA
GCTTTGTTTGAATGTGTTTGACTTTCATTTGATTCAAATAGCTTTGTTTGAATGTGTTTGACTTTCATTTGATTCAATT
AGCTTTGTAGAATGCATTTGACTTTCATTTGATTCAAATAGCTTTGTTTGAATGTGTTTGACTTTCATTTGATTCAAA
TAGCTTTGTTTGAATGTGTTTGACTTTCATTTGATTCAAATAGCTTTGTTTGAATGTGTTTGACTTTCATTTGATTCAA
ATAGCTTTGTTTGAATGTGTTTGACTTTCATTTGATTCAAATAGCTTTGTTTGAATGTGTTTGACTTTCATTTGATTCA
ATTAGCTTTGTAGAATGCATTTGACTTTCATTTGATTCAAATAGCTTTGTTTGAATGTGTTTGACTATCATTTGATTC
AAAAAGCTTTGTTTGAATGTGTTTGACTTTCATTTGATTCAATTAGCATTGTTGATTGCATTTGACTTTCATTTGATTC
AAAAAGCTTTGTTTGAATGTGTTTGACTTTCATTTGATTCAATTAGCTTTGTTGAATGCATTTGACTTTCATTTGATTC
AAAAAGCTTTGTTTGAATGTGTTTGACTTTCATTTGATTCAATTAGCTTTGTAGAATGCATTTGACTTTCATTTGATT
CAATTAGCTTTGTAGAATGCATTTGACTTTCATTTGATTCAAATAGCTTTGTTTGAATGTGTTTGACTATCATTTGAT
TCAAAAAGCTTTGTTTGAATGTGTTTGACTTTCATTTGATTCAATTAGCATTGTTGATTGCATTTGACTTTCATTTGAT
TCAAAAAGCTTTGTTTGAATGTGTTTGACTTTCATTTGATTCAATTAGCTTTGTAGAATGCATTTGACTTTCATTTGA
TTCAAATAGCTTTGTTTGAATGTGTTTGACTTTCATTTGATTCAATTAGCTTTGTTGAATGCATTTGACTTTCATTTGA
TTCAAATAGCTTTGTTTGAATGTGTTTGACTTTCATTTGATTCAAAAAGCTTTGTTTGAATGTGTTTGACTTTCATTTG
ATTCAATTAGCTTTGTTGAATGCATTTGACTTTCATTTGATTCAAAAAGCTTTGTTTGAATGTGTTTGACTTTCATTTG
ATTCAATTAGCTTTGTTGAATGCATTTGACTTTCATTTGATTCAAAAAGCTTTGTTTGAATGTGTTTGACTTTCATTTG
ATTCAATTAGCTTTGTAGAATGCATTTGACTTTCATTTGATTCAAATAGCTTTGTTTGAATGTGTTTGACTTTCATTTG
ATTCAATTAGCTTTGTTTGAATGCATTTGACTTTCATTTGATTCAAATAGCTTTGTTTGAATGTGTTTGACTTTCATTT
GATTCAAAAAGCTTTGTTTGAATGTGTTTGACTTTCATTTGATTCAATTAGCATTGTTGAATGCATTTGACTTTCATT
TGATTCAAAAAGCTTTGTTTGAATGTGTTTGACTTTCATTTGATTCAATTAGCTTTGTAGAATGCATTTGACTTTCAT
TTGATTCAAATAGCTTTGTTTGAATGTGTTTGACTTTCATTTGATTCAATTAGCTTTGTTGAATGCATTTGACTTTCAT
TTGATTCAAATAGCTTTGTTTGAATGTGTTTGACTTTCATTTGATTCAATTAGCTTTGTTGAATGCATTTGACTTTCAT
TTGATTCAAAAAGCTTTGTTTGAATGTGTTTGACTTTCATTTGATTCAATTAGCTTTGTTGAATGCATTTGACTTTCAT
TTGATTCAAAAAGCTTTGTTTGAATGTGTTTGACTTTCATTTGATTCAATTAGCTTTGTTGAATGCATTTGACTTTCAT
TTGATTCAAAAAGCTTTGTTTGAATGTGTTTGACTTTCATTTGATTCAATTAGCTTTGTTGAATGCATTTGACTTTCAT
TTGATTCAAATAGCTTTGTTTGAATGTGTTTGACTTTCATTTGATTCAATTAGCTTTGTTGAATGCATTTGACTTTCAT
TTGATTCAAAAAGCTTTGTTTGAATGTGTTTGACTTTCATTTGATTCAATTAGCATTGTTGAATGCATTTGACTTTCA
TTTGATTCAAAAAGCTTTGTTTGAATGTGTTTGACTTTCATTTGATTCAATTAGCTTTGTAGAATGCATTTGACTTTC

ATTTGATTCAAATAGCTTTGTTTGAATGTGTTTGACTTTCATTTGATTCAATTAGCTTTGTTGAATGCATTTGACTTTC
ATTTGATTCAAATAGCTTTGTTTGAATGTGTTTGACTTTCATTTGATTCAATTAGCTTTGTTAGAATGCATTTGACTTT
CATTTGATTCAAATAGCTTTGTTTGAATGTGTTTGACTTTCATTTGATTCAATTAGCTTTGTAGAATGCATTTGACTTT
CATTTGATTCAAATAGCTTTGTTTGAATGTGTTTGACTTTCATTTGATTCAATTAGCTTTGTTGAATGCATTTGACTTT
CATTTGATTCAAAAAGCTTTGTTTGAATGTGTTTGACTTTCATTTGATTCAATTAGCTTTGTTGAATGCATTTGACTTT
CATTTGATTCAAAAAGCTTTGTTTGAATGTGTTTGACTTTCATTTGATTCAATTAGCTTTGTTGAATGCATTTGACTTT
CATTTGATTCAAAAAGCTTTGTTTGAATGTGTTTGACTTTCATTTGATTCAATTAGCTTTGTTGAATGCATTTGACTTT
CATTTGATTCAAAAAGCTTTGTTTGAATGTGTTTGACTTTCATTTGATTCAATTAGCTTTGTTGAATGCATTTGACTTT
CATTTGATTCAAAAAGCTTTGTTTGAATGTGTTTGACTTTCATTTGATTCAATTAGCTTTGTTGAATGCATTTGACTTT
CATTTGATTCAAAAAGCTTTGTTTGAATGTGTTTGACTTTCATTTGATTCAATTAGCTTTGTTGAATGCATTTGACTTT
CATTTGATTCAAAAAGCTTTGTTTGAATGTGTTTGACTTTCATTTGATTCAATTAGCTTTGTTGAATGCATTTGACTTT
CATTTGATTCAAATAGCTTTGTTTGAATGTGTTTGACTTTCATTTGATTCAATTAGCTTTGTTGAATGCATTTGACTTT
CATTTGATTCAAATAGCTTTGTTTGAATGTGTTTGACTTTCATTTGATTCAATTAGCTTTGTTTGAATGCATTTGACTT
TCATTTGATTCAAATAGCTTTGTTTGAATGTGTTTGACTTTCATTTGATTCAAAAAGCTTTGTTTGAATGTGTTTGAC
TTTCATTTGATTCAATTAGCTTTGTAGAATGCATTTGACTTTCATTTGATTCAAATAGCTTTGTTTGAATGTGTTTGAC
TTTCATTTGATTCAATTAGCTTTGTAGAATGCATTTGACTTTCATTTGATTCAAATAGCTTTGTTTGAATGTGTTTGAC
TTTCATTTGATTCAAAAAGCTTTGTTTGAATGTGTTTGACTTTCATTTGATTCAATTAGCTTTGTTGAATGCATTTGAC
TTTCATTTGATTCAAATAGCTTTGTTTGAATGTGTTTGACTTTCATTTGATTCAATTAGCATTGTTGAATGCATTTGAC
TTTCATTTGATTCAAAAAGCTTTGTTTGAATGTGTTTGACTTTCATTTGATTCAAAAAGCTTTGTTTGAATGTGTTTG
ACTTTCATTTGATTCAATTAGCATTGTTGAATGCATTTGACTTTCATTTGATTCAAAAAGCTTTGTTTGAATGTGTTT
GACTTTCATTTGATTCAATTAGCTTTGTAGAATGCATTTGACTTTCATTTGATTCAAATAGCTTTGTTTGAATGTGTTT
GACTATCATTTGATTCAAAAAGCTTTGTTTGAATGTGTTTGACTTTCATTTGATTCAATTAGCATTGTTGAATGCATT
TGACTTTCATTTGATTCAAATAGCTTTGTTTGAATGTGTTTGACTTTCATTTGATTCAATTAGCATTGGACTTTCATTT
GATTCAAAAAGCTGTGTTTGAATGTGTTTGACTTTCATTTGATTCAATTAGCTTTGTAAAATGCATTTGACTTTCATT
TGATTCAAAAAGCTTTGTTTGAATGCATTTGACTTTCATTTGATTCAAATAGCTTTGTTTGAATGTGTTTGACTTTCA
TTTGATTCAATTAGCTTTGTAGAATGCATTTGACTTTCATTTGATTCAAATAGCTTTGTTTGAATGTGTTTGACTATC
ATTTGATTCAAAAAGCTTTGTTTGAATGTGTTTGACTTTCATTTGATTCAATTAGCATTGTTGAATGCATTTGACTTT
CATTTGATTCAAAAAGCTTTGTTTGAATGTGTTTGACTTTCATTTGATTCAATTAGCATTGTTGAATGCATTTGACTT
TCATTTGATTCAAAAAGCTTTGTTTGAATGTGTTTGACTTTCATTTGATTCAATTAGCTTTGTAGAATGCATTTGACT
TTCATTTGATTCAAATAGCTTTGTTTGAATGTGTTTGACTTTCATTTGATTCAAAAAGCTTTGTTTGAATGTGTTTGA
CTTTCATTTGATTCAATTAGCATTGTTGAATGCATTTGACTTTCATTTGATTCAAAAAGCTTTGTTTGAATGTGTTTG
ACTTTCATTTGATTCAATTAGCTTTGTAGAATGCATTTGACTTTCATTTGATTCAAATAGCTTTTTTTGAATGTGTTTG

ACTTTCATTTGATTCAAAAAGCTTTGTTTGAATGTGTTTGACTTTCATTTGATTCAATTAGCATTGTTGATTGCATTTG
ACTTTCATTTGATTCAAAAAGCTTTGTTTGAATGTGTTTGACTTTCATTTGATTCAATTAGCTTTGTAGAATGCATTT
GACTTTCATTTGATTCAAATAGCTTTGTTTGAATGTGTTTGACTTTCATTTGATTCAAAAAGCTTTGTTTGAATGTGT
TTGACTTTCATTTGATTCAAATAGCTTTGTTTGAATGTGTTTGACTTTCATTTGATTCAAATAGCTTTGTTTGAATGTG
TTTGACTTTCATTTGATTCAATTAGCTTTGTAGAATGCATTTGACTTTCATTTGATTCAAATAGCTTTGTTTGAATGTG
TTTGACTTTCATTTGATTCAAATAGCTTTGTTTGAATGTGTTTGACTTTCATTTGATTCAATTAGCTTTGTTTGAATGT
GTTTGACTTTCATTTGATTTAATTAGCTTTGTTTGAATGTGTTTGACTTTCATTTGATTCAATTAGCTTTGTTGAATGC
ATTTGACTTTCATTTGATTCAAAAAGCTTTGTTTGAATGTGTTTTACTTTCATTTGATTAAAAAAAGCTTTGCTTTAAT
GTGTTTGACTTTTATTTGTTTCAATGAGCTTTGTTTGAGTGTGTTTGACTTTTATATGATTCATTTAGCTTTGTTGAAT
GCATTTGACTTTCATTTGATTCAAAAAGCTTTGTTTGAATGTGTTTGACTTTCTTAAATGTGTTGGACTTTTCATTTG
ATTCAATTAGCTTTGTTTGAATGTGTTTGACTTTCATTTGATTAAAAAAAGCTTTGCTTTAATGTGTTTGACTTTTATT
TGTTTCAATGAGCTTTGTTTGAGTGTGTTTGACTTTTATTTGATTCATTTAGCTTTGTTGAATGCATTTGACTTTCATT
TGATTCAAAAAGCTTTGTTTGAATGTGTTTGACTTTCATTTGATTCAATTAGCTTTGTTTGAATGTGTTTGACTTTCAT
TTGATTCAATTAGCTTTGTAGAATGCATTTGACTTTCATTTGATTGAAATAGCTTTGTTTGAATGTGTTTGACTATCA
TTTGATTCAATTAGCTTTGTTTTAATGTGTTTGACTTTCATTTGATTCAATTTGCTTTGTTGATTTTATTTGACTTTCAT
TTAATTCAAAAAACATTGTTTGAAGTGATTGACTTTCATTTGAATCATTTAGCATTGTTGAATGCATTTGACTTTCAT
GATTCAATTTGCTTTGTTGAATGCATTTGACTTTCATTTGATTCAAAATACTTTGTTTGAATGTGTTTGACTTTCATTT
GATTCAATTAGCTTTGTAGAATGCATTTGACTTTCATTTGATTCAAAAAGCTTTGTTTGAATGTGTTTGACTTTCATT
TGATTCAAATAGCATTGTTGAATGCAATTGACTGTCATTTAATTTAAAAAGCTTTGTTTGAATGTGTTTGACTTTCAT
GTGATTCAATTAGCTTTGTAGAATGCATTTGACTTTCATTTGATTCATATAGCTTTGATTTGAATGTGTTTGACTTTC
ATTTGATTCAAAAAGCTTTGTTGAATGTGTTTGACTTTCATTTGATTCAAAAATCTTTGTTTGAATGTGTTTGACTTT
CATTTGATTCAATTAGCTTTGTTAGAATGCATTTGACTTTCATTTGATTCAAAAAGCTTTGATTGACTGT

Table of detected monomers

Indices	Period Size	Copy Number	Consensus Size	Percent Matches	Percent Indels	Score	A	C	G	T	Entropy (0-2)
8--6986	79	88.0	79	91	2	9793	24	11	16	48	1.78
8--6986	119	58.7	120	88	3	9631	24	11	16	48	1.78
4483--4701	67	3.1	67	80	15	269	25	12	15	46	1.81
4556--6986	40	60.8	40	83	4	2743	24	10	16	48	1.78
5939--6131	101	1.9	99	88	5	291	22	9	17	50	1.75

6506--6563	19	3.1	19	71	14	66	20	12	17	50	1.78

Consensus pattern (79 bp):

GTGTTTGACTTTCATTTGATTCAATTAGCTTTGTTGAATGCATTTGACTTTCATTTGATTCAAATAGCTTTGTTTGAAT

Consensus pattern (120 bp):

GTGTTTGACTTTCATTTGATTCAAATAGCTTTGTTTGAATGTGTTTGACTTTCATTTGATTCAAATAGCTTTGTTTGAA
TGTGTTTGACTTTCATTTGATTCAAATAGCTTTGTTTGAAT

Consensus pattern (67 bp):

GCATTTGACTTTCATTTGATTCAAAAAGCTTTGTTTGAATGTGTTTGACTTTCATTTGATTCAATTA

Consensus pattern (40 bp):

GACTTTCATTTGATTCAAATAGCTTTGTTTGAATGTGTTT

Consensus pattern (99 bp):

TGTGTTGGACTTTTATATGATTCAATTAGCTTTGTTGAATGCATTTGACTTTCATTTGATTAAAAAAAGCTTTGCTTT
AATGTGTTTGACTTTCTTAAA

Consensus pattern (19 bp):

TTGTTGAATGCATTTGACT

BLAST alignments:

Chenopodium quinoa clone 12-13p repeat region sequence Sequence ID: HM641822.1

Beta corolliflora minisatellite DNA, clone pBC1447 Sequence ID: AJ288880.1

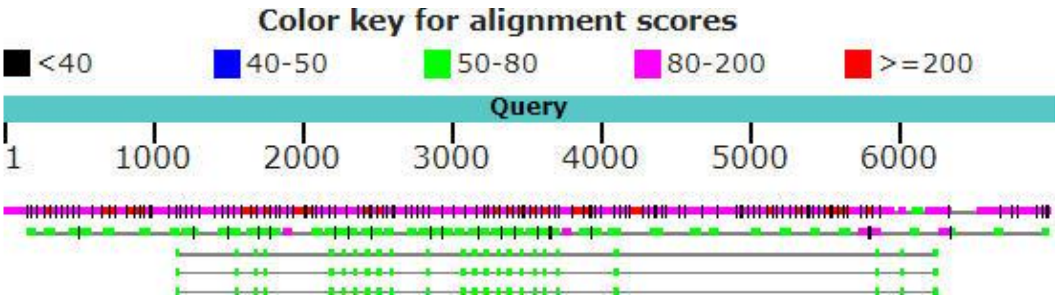

C. iljinii

Cluster 28

>CL28Contig57 (6247-278.0-1736891)

AAATGCATTCAAACAAAACTTTTTAAATCAAATGAAAATCAAACACAATCAAACAAAGAATCATGAATAAAATGAA
AGTCAAACACATTCAAACAAAGCTTTTTAAATCAAATGAAAGTCAAAATGCATCAATTGAAAATCAAATACATTAA
ACAAAGCTTTTTGATTCAAATGAAATTCAAGCACATTTAAACAAAGATTCTTGAATAAAATGAAAGTCAAACACATT
CAAACAAAGCTTTTTAAATCAAATGAAAGTCAAAATGCATTCAACAAATCTAATTGAGTCAAATTAAAGTCAAACA
CATTCAAACAAAGCTTTTTGAATCAAATGAAAGTCAAATGCATTCAACAAAGCTTTTGAATCAAATGAAAGTCAAA
CACATTCAAACAGAGCTTTTTGAATCAAATGAAAGTCAAATGCATTCAACAAAGCTAATTGAGTCAAATGAAAGTC
AAACACATTCAAACAAAGCTTTTTGAATCAAATGAAAGTCAAATGCATTCAACAAAGCTAATTGAGTCAAATGAAA
GTCAAACACATTCAAACAAAGCTTTATAAATCAAATGAATGTCAAAATGCATTCTACAAAGCTAATTGAGTCAAATT
AAAGTCAATAACATTCAAACAAACCGTTTATAATCAAATGAAAGTCAAATGCATTCAACAAAGCTTTTTGAATCAAA
TGAAAGTCAAATGCATTCAACAAACCTTTTTGAATCAAATGAAAGTCAAATGCATTCAACAAAGCTAATTGAGTCA
AATGAAAGTCAAACACATTCAAACAAAGCTTTATAAATCAAATGAATGTCAAAATGCATTCTACAAAGCTAATTGA
GTCAAATTAAAGTCAATAACATTCAAACAAACCGTTTATAATCAAATGAAAGTCAAACACATTCAAACAGAGCTTTT
TGAATCAAATGAAAGTCAAATGCATTCAACAAAGCTAATTGAGTCAAATGAAAGTCAAACACATTCAAACAAAGCT
TTATAAATCAAATGAATGTCAAAATGCATTCTACAAAGCTAATTGAGTCAAATTAAAGTCAATAACATTCAAACAAA
CCGTTTATAATCAAATGAAAGTCAAATGCATTCAACAAAGCTTTTTGAATCAAATGAAAGTCAAATGCATTCAACAA
ACCTTTTTGAATCAAATGAAAGTTGAATACATTCAAACAAAGCTTTTTGAATCAAATGAAAGTCAAATACATTCAAC
AAATCTTTTTGAATCAAATGAAATTCAAGCACATTCAAACAAAGATTCTGAAAGACAAACACATTCAAACAAATCTT
TTTGAATAAAATGAAAGTCAAATGCATTCAACAAAGCTAATTGAGTCAAATGAAAGTCAAACACATTCAAACAAAG
CTTTATAAATCAAATGAATGTCAAAATGCATTCTACAAAGCTAATTGAGTCAAATTAAAGTCAATAACATTCAAACA
AACCGTTTATAATCAAATGAAAGTCAAATGCATTCAACAAAGCTTTTTGAATCAAATGAAAGTCAAATGCATTCAAC
AAACCTTTTTGAATCAAATGAAAGTTGAATACATTCAAACAAAGCTTTTTGAATCAAATGAAAGTCAAATACATTCA
ACAAAGCTTTTTGAATCAAATGAAATTCAAGCACATTCAAACAAAGATTCTTGAATAAAATGAAAGACAAACACAT
TCAAACAAAGCTTTTTGAATAAAATGAAAGTCAAATGCATTCAACAAAGCTAATTGAGTCAAATGAAAGTCAAACA
CATTCAAACAAAGCTTTTTAAATCAAATGAAAGTCAAAATACATTCAACAAAGCTAATTCAGTAAAATAAAAGTCA
AACACATTCAAACAAAGCTTTTTGATCAAAATGAAAGTCAAATGCATTCAACAAACCTTTTTGAATCAAATGAAAGT
TGAATACATTCAAACAAAGCTTTTTGAATCAAATGAAAGTCAAATACATTCAACAAAGCTTTTTGAATCAAATGAAA
TTCAAGCACATTCAAACAAAGATTCTTGAATAAAATGAAAGACAAACACATTCAAACAAAGCTTTTTGAATAAAAT
GAAAGTCAAATGCATTCAACAAAGCTAATTGAGTCAAATGAAAGTCAAACACATTCAAACAAAGCTTTTTAAATCA
AATGAAAGTCAAAATGTATTAAACAAAGCTAATTGAGTCAAATTAAAGTCAAACACATTCAAACAAAGCTTTTTGA
ATCAAATGAAAGTCAAATGCATTCAACAAATTTTTTTAATCAAATGAAAGTCAAATGCATTCAACAAATCTTTTTG
AATTAAATGAGTTGAATACATTCAAACAATGATTTTTAAATCAAATGAAAGTCAAATACATTCAACAAAGCTTTTTG
AATCAAATGAAATTCAAGCACATTCAAACAAAGATTCTTGAATAAAATGAAAGACAAACACATTCAAACAAAGCTT

TTTGAATAAAATGAAAGTCAAATGCATTCAACAAAGCTAATTGAGTCAAATGAAAGTCAAACACATTCAAACAAAG
CTTTTTAAATCAAATGAAAGTCAAAATGTATTAAACAAAGCTAATTGAGTCAAATTAAAGTCAAACACATTCAAACA
AAGCTTTTTGAATAAAATGAAAGTCAAATGCATTCAACAAAGCTAATTGAGTCAAATGAAAGTCAAACACATTCAA
ACAAAGCTTTTTAAATCAAATGAAAGTCAAAATGTATTAAACAAAGCTAATTGAGTCAAATTAAAGTCAAACACAT
TCAAACAAAGCTTTTTGAATCAAATGAAAGTCAAATGCATTCAACAAAAGTTTTTGAACCAAATGAATGTCAAATG
CATTCAAACAAAGCTTTTTAAATCAAATGAAAGTCAAAATGTATTAAACAAAGCTAATTGAGTCAAATTAAAGTCA
AACACATTCAAACAAAGCTTTTTGAATCAAATGAAAGTCAAATGCATTCAACAAAAGTTTTTGAACCAAATGAATG
TCAAATGCATTCAACAAAGATTTTTAAATCAAAAGAAAGTCAAAATGTATTAAACAAAGCTAATTGAGTCAAATTA
AAGTCAAACACATTCAAACAAAGCTTTTTGAATCAAATGAAAGTCAAATGCATTCAACAAAAGTTTTTGAACCAAA
TGAATGTCAAATGCATTCAACAAAGATTTTTAAATCAAAAGAAAGTCAAAATGTATTAAACAAAGTTAATTGAGTC
AAATTAAAGTCAAACACATTCAAAAAAAGCTTTTTGAATCAAATGAATGTCAAATGCATTCAACAAAGCTTTTTAAA
TCAAATGAAAGTCAAATGCATTCAACAAATCTTTTAGAATCAAATGAAAGTTAAACATATTCAAACAAAGCTTTT
TGAATCAAATGAAGGTAAAATGCATTTAACAAAGCTTTTTGGATCAAACGGAAGTCAAGCACATTCAAACAAAGAT
ACGTGAATAAAATGAAGGTCAAACACATTCAAACAAAGCTTTTTGAACCAAATGAATGTCAAATGCATTCAACAAA
GATTTTTAAATCAAAAGAAAGTCAAAATGTATTAAACAAAGTTAATTGAGTCAAATTAAAGTCAAACACATTCAAA
AAAAGCTTTTTGAATCAAATGAATGTCAAATGCATTCAACAAAGCTTTTTAAATCAAATGAAAGTCAAATGCATTCA
ACAAATCTTTTAGAATCAAATGAAAGTTAAACATATTCAAACAAAGCTTTTTGAATCAAATGAAGGTAAAATGCATT
TAACAAAGCTTTTTGGATCAAACGGAAGTCAAGCACATTCAAACAAAGATACGTGAATAAAATGAAGGTCAAACA
CATTCAAACAAAGCTTTTTGAATCAATTGAAAGTCAAATGAATTCAACAAAGCTAATTGAGTCTAATGAAAGTCAA
ACACATTCAAACAAAGCTTTTCAAATCAAATGAAAGTCAAAATGTATTAAATAAAGCTAATTGAGTCAAATTAAAG
TCAAACACCTTCAAACAAAGCTTTTTGAATCAAATGAAAGTCAAATCCATTCAACAAAAGTTTTTGAATCAAATGAA
AGTCAAACACATTCAAACAAAGCTTTTTGAATCAATTGAAAGTCAAATGAATTCAACAAAGCTAATTGAGTCTAAT
GAAAGTCAAACACATTCAAACAAAGCTTTTCAAATCAAATGAAAGTCAAAATGTATTAAATAAAGCTAATTGAGTC
AAATTAAAGTCAAACACCTTCAAACAAAGCTTTTTGAATCAAATGAAAGTCAAATCCATTCAACAAAAGTTTTTGAA
TCAAATGAAAGTCAAATGCATTCAAACAAATCTTTTTAAATCAAATGAAAGTCAAAAAGTATTAAACAAAGTTAATT
GAGTCAAACACATTCAAACAAAGCTTTTTGAATCAATTGAAAGTCAAATGAATTCAACAAAGCTAATTGAGTCTAA
TGAAAATCAAACACATTCAAACAAAGCTTTTTAAATCAAATGAAAGTCAAAATGTATTAAATAAAGCTAATTGAGT
CAAATTAAAGTCAAACACATTCAAACAAAGCTTTTTGAATCAATTGAAAGTCAAATGAATTCAACAAAGCTAATTG
AGTCTAATGAAAGTCAAACACATTCAAACAAAGCTTTTCAAATCAAATGAAAGTCAAAATGTATTAAATAAAGCTA
ATTGAGTCAAATTAAAGTCAAACACCTTCAAACAAAGCTTTTTGAATCAAATGAAAGTCAAATCCATTCAACAAAA
GTTTTTGAATCAAATGAAAGTCAAATGCATTCAAACAAATCTTTTTAAATCAAATGAAAGTCAAACACATTCAAACA
AAGATTCATGAATAAAATGAAAGTCAAACACATTCAAACAAAGCTTTTTAAATCAAATGAAAGTCAAAATGCATTC

AACAAATCTAATTGAGTCAAATTAAAGTCAAACACATTCAAACAAAGCTTTTTGAATCAAATGAAAGTCAAATGCA
TTCAACAAAGCTTTTGAATCAAATGAAAGTCAAACACATTCAAACAGAGCTTTTTGAATCAAATGAAAGTCAAATG
CATTCAACAAAGCTAATTGAGTCAAATGAAAGTCAAACACATTCAAACAAGCTTTTTGAATCAAATGAAAGTCAAA
TGCATTCAACAAATCTTTTTAAATCAAATGAAAGTCAAACACATTCAAACAAAGATTCTTGAATAAAATGAAAGTCA
AACACATTCAAACAAAGCTTTTTGAATCAAATGAAAGTTAAAATGCATTCAACAAATCTAATTGAGTCAAATTAAA
GTCAAACACATTCAAACAAAGCATTTTGAATCAAATGAAAGTCAAATGCATTCAACAAAGCTTTTTGAATCAAATG
AAAGTCAAACACATTCAAACAAAGCTTTTTGAATCAAATGAAAGTCAAACACATTCAAACAAAGCTTTTTAAATCAA
ATGAAAGTCAAAATGCATTCAACAAATCTAATTGAGTCAAATTAAAGTCAAACACATTCAAACAAAGCTTTTTGAAT
CAAATGAAAGTCAAATGCATAAACAAAGCTTTTGAATCAAATGAATTCAAACACATTCAAACAGAGCTTTTTGAAT
CAAATGAAAGTCAAATGCATTCAACAAAGCTAATTGAGTCAAATGAAAGTCAAACACATTCAAACAAAGCTTTATA
AATCAAATGAATGTCAAAATGCATTCTACAAAGCTAATTGAGTCAAATTAAAGTCAATAACAATCAAACAAACCGT
TTATAATCAAATGAAAGTCAAATGCATTCAAACAAACCGTTTATAATCAAATGAAAGTCAAATGCATTCAACAAAG
TTTTTTGAATCAAATGAAAGTCAAATGCATTCAACAAACCTTTTTGAATCAAATGAAAGTTGAATACATTCAAACAA
AGCTTTTTGAATCAAATGAAAGTCAAATACATTCAACAAAGCTTTTTGAATCAAATGAAATTCAAGCACATTCAAAC
AAAGATTCTTGAATAAAATGAAAGACAAACACATTCAAACAAAGCTTTTTGAATAAAATGAAAGTCAAATGCATTG
AACAAAGCTAATTGAGTCAAATGAAAGTCAAACACATTCAAACAAAGCTTTTTAAATCAAATGAAAGTCAAAATGT
ATTAAACAAAGCTAATTGAGTCAAATTAAAGTCAAACACATTCAAACAAATCCTTCCATGGTGATTTGATTTTGCTG
GTTTTTCTTCAGCTTAATCGGTTTCAATGGCTCGAAAGAAGAAACAATCCACCCCCATACCTCA

Table of detected monomers

Indices	Period Size	Copy Number	Consensus Size	Percent Matches	Percent Indels	Score	A	C	G	T	Entropy (0-2)
6--123	40	3.0	40	79	0	155	55	16	6	22	1.64
128--6156	40	152.8	40	76	6	5035	47	15	10	26	1.78
1--742	158	4.9	144	79	11	516	48	15	10	25	1.77
128--6156	79	76.4	80	83	5	6141	47	15	10	26	1.78
128--6156	159	38.2	158	84	4	6076	47	15	10	26	1.78

Consensus pattern (40 bp):

CATTCAAACAAAGCTTTTTAAATCAAATGAAAGTCAAACA

Consensus pattern (40 bp):

ATCAAATGAAAGTCAAACACATTTAAACAAAGCTTTTTGA

Consensus pattern (144 bp):

AAATACATTCAAACAAAGCTTTTTGAATCAAATGAAAATCAAACACATTAAACAAAGATTCTTGAATAAAATGAAA

GTCAAACACATTCAAACAAAGCTTTTTAAATCAAATGAAAGTCAAAATGCATCTAATTGACAAATTAC

Consensus pattern (80 bp):

ATCAAATGAAAGTCAAACACATTCAAACAAAGCTTTTTGAATCAAATGAAAGTCAAATACATTCAAACAAAGCTTTT

TGA

Consensus pattern (158 bp):

ATCAAATGAAAGTCAAATACATTCAACAAAGCTTTTTGAATCAAATGAAAGTCAAATACATTCAAACAAAGCTTTTT

GAATCAAATGAAAGTCAAACACATTCAAACAAAGCTTTTTGAATCAAATGAAAGTCAAAACATTCAAACAAAGCTT

TTTGA

BLAST alignments:

Chenopodium quinoa clone 12-13p repeat region sequence. Sequence ID: HM641822.1

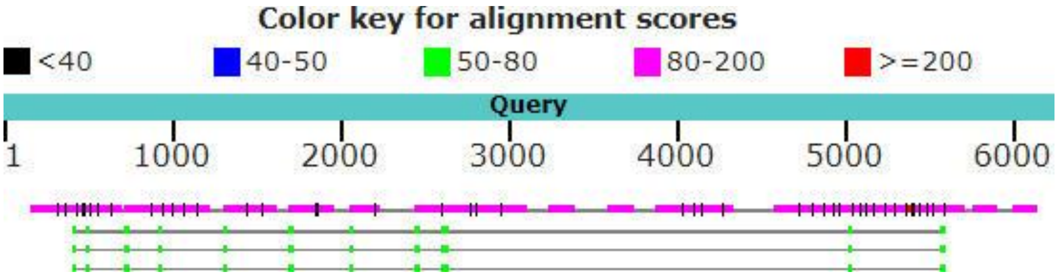

C. vulvaria

CLUSTER 28

>CL28Contig18 (3488-247.5-863128)

AATCTCACATACAAACAAATCTAATGTATCAAATGAAAGTCAGACACATTCAAACAAAGCTGATCTTATCAAATGA

AAGTCAAACACATTCAAACAAAGGTAATACAATCAAATGAATTCAACAAAGCTAATTCACTCAAATGAAAGTCAAA

TGCGTTTAACAAAGCTAAATGAATCAAATGAAAGTCAAACACATTTAATCAAAGCTAATTAAATCAAATGAAAGTC

AAATGCATTTAAGAAAGCTAATTGAATTAAATGAAAGTCAAACACATTCAAGCAAAGATAATTAAATCAAATGAAA

GTCAAATGAGCTCAACAAAGGTAGTTGGATCAAATGATAGTCAAACACATTCAAACAAAGCTAATTGAATCTAAAC

ACATTCAAACACAGCTAATTGAATCAAATGAAAGTCAAATGCATTCTACAAAGCTAATTCACTCAAATGAAAGTCA

AATGCCTTCAACAAAGATAAATGAATCAAATGAAATTCAAACACATTCAATTAAAGCTAATAGAATCAAATGTAAG

TTAAATGCATTCAACAAACCTATGTCACTCAAATGAAAGTCAAATGCATTCTACAAAGATAAATGAATCAAATGAA

AGTCTAACACATTCAATCAAAGCTAATTGCATCAAATGAAAGTCAAACACATTCAAATAACGCTAAATTGTATCGA

ATGAAAGTGAAAGAAATTCAAACAAAGATATTTAAATCAAATGCATTCAACAAAGCTAATTGAATCAAATGAAAGT
CAAAATCATTTAAACAAAGCTAATTAAATAAAATAAAAGTCAAAAACATACAAACAAATCTAATGTAATCAAATGA
AAGTCAGATGCATTCAACAAAGCAAATTAAATCAAATGAAAGTCAAACTCATTCAAACAAAGCTAATTGAATTAAA
TGATAGTCAAACACATTAAAACAAAGCTAATCGAATCAAATGAAAGTCAAACACATTCAAACAAAGCTGATCTTAT
CAAATGAAAGTCAAACACATTCAAACAAAGCTAATACAATCAAATGAATTCAACAAAGCTAATTCACTCAAATGAA
AGTCAAATGCACTCAACAAATAAAAGACTAACACATTTAATCAAAGCTAATTGAATCAAATGAAGAACAATGCATT
TAATAAAGCTAATTGAATTAAATGAAAATCAAATCAAAAAAAGATAATTGAATCAAATCAAAGTCAAATGCATTAA
ACAAAGCTAATTGAATCAAATGATAGTCAAACACATTCAAACAAAGCTAACAAAATCAAATGAAAGTCATATGCAT
TCACGAAAGCTAATTGAATCAAATGATAGTCAAACATATTCAAACAAAGGTAATTTAATCAAATGAAAGAAAAACA
CATTCAAACAAAGCGAATTCTCTCAAATGAAAGTCAAATGCATTCAACAATGCTAATTGAATCAAATGATAGTCAA
ACACTTTCAAACAAAGCTAATTGAATCAAATGAAAGTCAGACACATTCAAACAAAGCTGATTAAATCAAATGACGG
TCAAACACATTCAAAAAAACCTTATTGAATCAAATGATAGTCAAACACTTTCAAACAAAGCTAATTGAATAGATTAA
AAGTCAATTGCATTCAACAAATCTAATTGAATCAAATGATAGTCAATCACATTCAAACAAAGATAATTGAATCAAAT
GAAAGTTAAACACAATCAAAGAAAACTTATAGAATCAACTGAAAGTCAAATGCGTTCAACAAAGCTAATTCACTCA
AATCAAAGTCAAATGCTTTCAATAAAGATAAATGAATCAAATGCAAGTCAAGGACATTCAACCAAAGCTAATTGAT
CAAATGAAAGTCAAATGCATTTAAATAAAGCTAGTTGAACCAAATGGTAGTCAAACACATTAAAACAAAGCTAATT
AAATCAATTTTTTAGTCCATTGCATTCATCAAATCTAATTGAATCAAATGATAGTTAAACACATTCAAACATGGGTA
ATTGAATCAAATGAAATTTAAACGCAATCAAACAAAGCAAATTGAATCAAACCAAACTTATGGATTCAAATGAGAG
TCATACACATTCTATCAAAGCTAAATGAATCAAATGAAAGTTAAACACAATCAAAGAAAACTTATAGAATCAACTG
AAAGTCAAATGCGTTCAACAAAGCTAATTCACTCAAATCAAAGTCAAATGCTTTCAATAAAGATAAATGAATCAAA
TGCAAGTCAAGGACATTCAACCAAAGCTAATTGATCAAATGAAAGTCAAATGCATTTAAATAAAGCTAGTTGAACC
AAATGGTAGTCAAACACATTAAAACAAAGCTAATTAAATCAATTTTTTAGTCCATTGCATTCATCAAATCTAATTGA
ATCAAATGATAGTTAAACACATTCAAACATGGGTAATTGAATCGAATGAAAGTTAAACACAATCAAACAAAACTTA
TAGAATCAAATGAGAGTCATACACATTCAATCAAAGCTAAATGCATCAAATGAAAGTCAAACACATTCAAAAAAAG
CAAATTGAATCTAATGAAAGTCAAAGAAATTCAAACAAAGATATTTGAATCAAATAAAAGTCAAATGCATTCAACA
AAGCTAATTGAATCAAATGAAAGTCAAAATCATTCAAACAAAGATATTTGAATCAAATGAAAGTCAAACAGATTTA
AACAAAGCTAATTAAATCAAATGAAATTCAAACACAATCAAACCAAACTAATAGAATCAAATGGAAGTCAAATGCA
TTCAACAGTGCTAATTCACTCAAATGAAAGTCAAATGCATTCAACAAAGCTAAATGAATCAAATGAAAGTCAAACA
CATTCAATCAAAGCTAATTGCATCAAATGAAAGTCAAACTCATTCAAATGACGCTAAATTGTATCAAATGAAAGTC
AAAGAAATTCAAACAAAGATATTTGAATCAAATAAAAGTCAAATGCATTCAACAAAGCTAATTGAATCAAATGAAA
GTCAAAATCATTCAAACAAAGATATTTGAATCAAATGAAAGTCAAACAGATTTAAACAAAGCTAATTAAATCAAAT
GAAATTCAAACACAATCAAACCAAACTAATAGAATCAAATGGAAGTCAAATGCATTCAACAGTGCTAATTCACTCA

AATGAAAGTCAAATGCATTCAACAAAGCTAAATGAATCAAATGAAAGTCAAACACATTCAATAAAAACTAACTGCA
TCAAATGAAAGACAAAGTATTAAAACAAAGCTAATCAAATAAAATGAAAGTTAAATGCATTCAAAAGTGCTAGTTC
ACTCAAATGCAATCAACAAAGCTAAACGAATCGAATGAAAGTCAAACACATTCAAACAAACCTAATAGAATCAAAT
GTAAGTTAAATAAATTCAAACAATGATATTTGAATCAAATAAAAGTCAAATGCATTCAACAGTGAAATAATAGAAT
CAAATGGAAGTCAATTGCATTCAACAGTGCTAATTCACTCAAATGAAAGTCAAATGCATTCAAC

Table of detected monomers

Indices	Period Size	Copy Number	Consensus Size	Percent Matches	Percent Indels	Score	A	C	G	T	Entropy (0-2)
6--117	40	2.8	40	85	4	138	50	18	9	20	1.75
88--145	28	2.0	29	86	3	82	51	17	8	22	1.72
117--3488	79	42.6	78	71	11	1595	49	15	11	23	1.77
117--3487	40	85.2	40	72	9	1837	49	15	11	23	1.77
347--405	30	2.0	30	96	0	109	49	22	6	22	1.73
355--419	30	2.2	30	83	5	78	50	18	9	21	1.74
700--755	28	2.0	29	85	3	78	50	14	8	26	1.72
721--3260	119	21.1	118	70	12	589	49	15	10	23	1.76
1007--1064	28	2.0	29	90	3	91	51	18	6	22	1.70
1391--3054	416	4.1	397	82	7	1572	48	15	11	24	1.78
2071--3488	358	4.0	355	84	4	1509	49	15	11	23	1.77
3381--3488	39	2.7	40	84	2	137	46	16	12	24	1.82

Consensus pattern (40 bp):

CACATTCAAACAAAGCTAATCTAATCAAATGAAAGTCAAA

Consensus pattern (29 bp):

ATTCAAACAAAGCTAATACAATCAAATGA

Consensus pattern (78 bp):

ATTCAACAAAGCTAATTGAATCAAATGAAAGTCAAATGCATTCAACAAAGCTAATTGAATCAAATGAAAGTCAAAC
AC

Consensus pattern (40 bp):

ATTCAAACAAAGCTAATTGAATCAAATGAAAGTCAAACAC

Consensus pattern (30 bp):

AAACACATTCAAACAAAGCTAATTGAATCT

Consensus pattern (30 bp):

TCAAACAAAGCTAATTGAATCTAAACAAAG

Consensus pattern (29 bp):

ATTCAAACAAAGATAATTAAATCAAATGC

Consensus pattern (118 bp):

TCAAATGCATTCAACAAAGCTAATTAATCAAATGAAAGTCAAAATCATTCAAACAAAGCTAATTAAATAAAATAAA
AGTCAAAAACATTAAAACAAAGCTAATCGAATCAAATGAAAG

Consensus pattern (29 bp):

ATTCAAACAAAGCTAATACAATCAAATGA

Consensus pattern (397 bp):

TCAAATGCAAGTCAAGGACATTCAACCAAAGCTAATTGATCAAATGAAAGTCAAATGCATTTAAATAAAGCTAGTT
GAACCAAATGGTAGTCAAACACATTAAAACAAAGCTAATTAAATCAATTTTTTAGTCCATTGCATTCATCAAATCTA
ATTGAATCAAATGATAGTTAAACACATTCAAACATGGGTAATTGAATCGAATGAAAGTCAAACACAATCAAACAAA
ACTTATAGAATCAAATGAGAGTCATACACATTCAATCAAAGCTAAATGAATCAAATGAAAGTTAAACACAATCAAA
GAAAACTTATAGAATCAACTGAAAGTCAAATGCGTTCAACAAAGCTAATTCACTCAAATCAAAGTCAAATGCTTTC
AATAAAGATAAATGAA

Consensus pattern (355 bp):

CAAAGCTAAATGAATCAAATGAAAGTCAAACACATTCAATCAAAACTAATTGCATCAAATGAAAGTCAAACTCATT
CAAAAAAGCTAATTGAATCAAATGAAAGTCAAATGAATTCAACAAAGATATTTGAATCAAATAAAAGTCAAATGCA
TTCAACAAAGCTAATTGAATCAAATGAAAGTCAAAATCATTCAAACAAAGATATTTGAATCAAATGAAAGTCAAAC
AGATTTAAACAAAGCTAATTAAATCAAATGAAAGTCAAACACAATCAAACCAAACTAATAGAATCAAATGGAAGTC
AAATGCATTCAACAGTGCTAATTCACTCAAATGAAAGTCAAATGCATTCAA

Consensus pattern (40 bp):

AATCAAATGAAAGTCAAATGCATTCAACAGTGAATAATAC

BLAST alignments:

Chenopodium quinoa clone 12-13p repeat region sequence. Sequence ID: HM641822.1

Beta corolliflora minisatellite DNA, clone pBC1447 Sequence ID: AJ288880.1

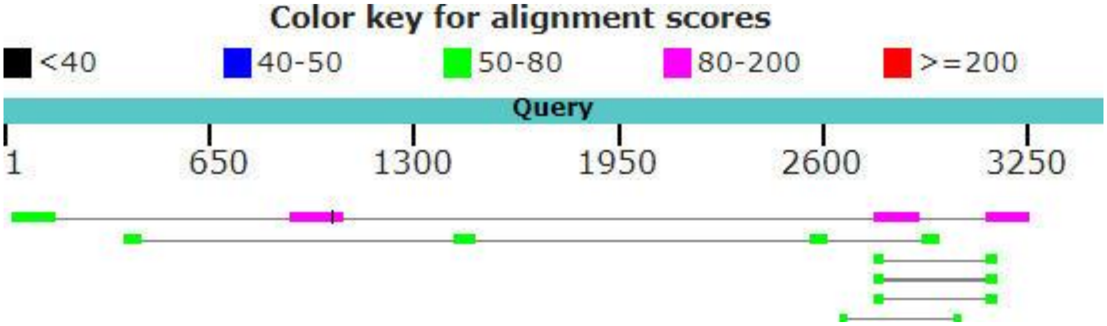

CLUSTER 94

>CL94Contig11 (2506-87.2-218590)

ATCTAATTAAATAATATGAAGGTCAAAGACGTTGAAAGAAAGTTCATTGAATCAAACGAAACTGAAATGTTTTCAA
CAAAGCCATTTTAGTCAAAATGAAAGTCAAACACAATGATATAAAGTTAATTAAATCAAATGAAAGTCAAATGACT
TCAACAAAGGCTAATTGAATCAATTGAAAGTCAAATGCATTCAACAATGCTAATTGAGTCAAATGAAACTTAAATA
CATTGAAACACATTGAAACAAAGTTAATTGAATAAAATGAAAGTCAAATGCACTCAACAAAGCAAATTGAATAAAA
TGAAAATCAAAAACATTGAAGCCAAGTTAATTGAATAAAGCATAAGTAAAACACATTAAAAACAAAGTTAATTGAA
TCAAATGAAAGTCAAATGATTTTAAAAAAGCTAATTGAATCCAATGACAGTCAAACACCTTGAAACAGAGTTAATT
GAATCAAATAAATCTCAAATGCTTTCAACAAAGCTAATTGAATCAAATGAAAGTCAAATGCATTCAAAAAAGCTAA
TTGAATCAAATGAAAGTCAAACACATTGAAACAAAGTTAGCTGAATCAAATGAAAGTCAAATGCTTTCAACAAAGC
TAATTGAATCAAATGAAAGTCAAATGCATTCAAAAAAGCTAATTGAATCAAATGAAAGTCAAACACATTGAAACAA
AGTTAGCTGAATCAAATGAAAGTCAAATGCTTTCAACAAATCTAATTAAATCAAAAGAAAGTCAAATTCACTCAAC
AAAGCTAATTGAATCAAATGAAAGTCAAACTCATTCAACAAAGCTAATTGAATAAAAAAAAAAGTCAATCACATTA
AAACAAAGCCGATTAAATCGAATGAAAGTCAAATGCTCTCAACAGAGCTGATAGAATGAAATGAAAGTCAAATGC
CTTGAAACAAAGGTAGTTGAATAAAAATATATGTAAAATGATTTTAACAAAGCTATATAAATCAAATGAAAGTCAA
ACGCATTAAAAAATCCTAATGGAATCAAATGAATGTCATACACATTGAAACATAGTTTATTGAATCAAATGAAACT
CAAATGCTTTCAACGAAGCTAATTAAAACATAGAAAACCCAAATACATTGAAATATAGTTAATTCAATCAAATGAC
AGTCAAATGATTTACACAATCGTAATTGAATCAAATGAAAGTCAAATTCTTTTGACAAACGTAATTGAATCAAATGA
AAGTCAAACACATTGAAACAAAGTAAATTGAATCAAATGAAAGTCAAATGTTTTCAACAAAGCTAATTGAATCAAA
TGAAAGTCAAACGCATTTAACAAAGCTAATTGAATCATATGAAAGTCAAATTCATTCAACAAAGCTAATTGAATCA
AATGAAAGTCAAACGCATTTAACAAAGCTAATTGAATCATATGAAAGTCAAATTCATTCAACAAAGCAAATTGAAT
CAAATGAAAGACAAAAACACACTGAAATAAAGTTAACTGAATCAAATGAAAGTCAAATGATTTCAACAAAGCTAA
TCAAATAAATTGAAAGTCAAATGCATTAAACAAAGCTGATTGAATCAAATGAAATTCAAACACATTGAAACAAAGT
TAATTGAATCAATTGAAAGTCAAATGCATTCGACAAATCTAATTAAATCATATGAAGGTCAAACACGTTGAAACAA
AGTTCATTGAATCAAACGAAACTCAAATGCTTTCAACAAAGCTATTTTAATCAAAATGAAAGTCAAACACAATGAT
ATAAAGTTAATTAAATCAAATGAAAGTCAAATGACTTCAACAAAGGCTAATTGAATCAATTGAAAGTCAAATGCAT

TCAACAATGCTAATTGAGTCAAATGAAACTTAAATACATTGAAACAAAGTTAATTGAATCAAATGAAAGTCAACAG
ATTTCAACAAAGCTAATTAAATCAATTGAAAGTCAAATGCATTCAAAAAAGCTAATTGAACCTAAGAAAGTCAAAT
GCATTCAAAAAAGGTAATTGAATTAAATGAAAGTCAAACACATTGAAACAAAGTTAATTGAATCAAATGAAATTAA
AATTCTTTCAACAAAGCTAATTGAATGAAATGAAAGTCAAATGCACTCAACAAAGCAAATTGAATAAAATGAAAAT
CAAAAGCATTGAAGCAAAGTTAATTGAATCAAGCATAAGTAAAACACATTAAAAACAAAGTTAATTGAATCAAATG
AAAGTCAAATGATTTTAAAAAAGCTAATTGAATCCAATGACAGTCAAACACCTTGAAACAGAGTTAATTGAATCAA
ATAAATCTCAAATGCTTTCAACAAAGCTAATTGAATCAAATGAAAGTCAAATGCATTCAAAAAAGCTAATTGAATC
AAATGAAAGTCAAACACATTGAAACAAAGTTAGCTGAATCAAATGAAAGTCAAATGCTTTAAACAAAGCTAATTGA
CTCAAATGAAAGTCAAACACACTAAAACAACGTTAATTGAGTCAATTGAAAGTCAATTGCTTTCAACAAAGCTAA

Table of detected monomers

Indices	Period Size	Copy Number	Consensus Size	Percent Matches	Percent Indels	Score	A	C	G	T	Entropy (0-2)
46--220	40	4.4	39	72	7	136	47	13	13	25	1.79
155--324	89	1.9	90	85	1	234	51	12	12	22	1.73
245--2506	39	57.3	39	76	6	1806	49	14	12	24	1.77
245--2506	118	19.1	118	78	6	1795	49	14	12	24	1.77
1311--1369	18	3.1	18	68	13	55	49	15	10	25	1.76
1928--1988	19	3.2	19	65	17	54	52	14	13	19	1.74
238--2506	79	28.7	79	76	7	1642	49	14	12	24	1.77

Consensus pattern (39 bp):

ATTGAATCAAATGAAAGTCAAATGCATTCAACAAAGCTA

Consensus pattern (90 bp):

AACAAAGGCTAATTGAATAAAATGAAAGTCAAATGCACTCAACAAAGCAAATTGAATAAAATGAAAATCAAAAAC
ATTGAAACACATTGA

Consensus pattern (39 bp):

AACAAAGCTAATTGAATCAAATGAAAGTCAAATGCATTC

Consensus pattern (118 bp):

AACAAAGCTAATTGAATCAAATGAAAGTCAAATGCATTCAAAAAAGCTAATTGAATCAAATGAAAGTCAAACACAT
TGAAACAAAGTTAATTGAATCAAATGAAAGTCAAATGCTTTC

Consensus pattern (18 bp):

AACAAAGCTAATTGAATC

Consensus pattern (19 bp):

AAAGTCAAATGCATTCAAA

Consensus pattern (79 bp):

ACATTGAAACAAAGCTAATTGAATCAAATGAAAGTCAAATGCTTTAAACAAAGCTAATTGAATCAAATGAAAGTCA
AAT

BLAST alignments:

Beta corolliflora minisatellite DNA, clone pBC1447 Sequence ID: AJ288880.1

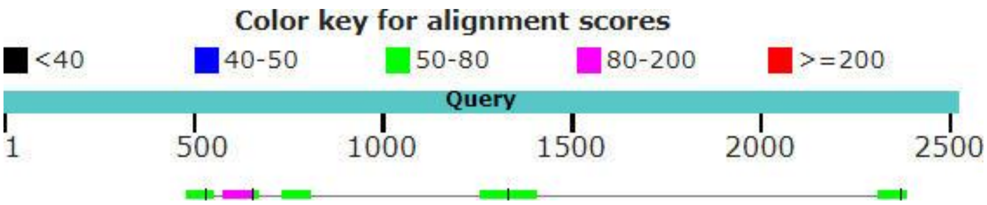

Cluster 112

>CL112Contig18 (2943-182.8-537871)

TCTTTGTTTGAATGTGTTTGACTTTCATTTCATACAATTAGCTTTATAAATGCATTTAACTTTCATTTGATTTAATTTG
CTTTGTTGAAAGTGTTTGACTTTCATTTGATTCATTTAGCTTTGTTAAATGCATTTGACTTTCATTTAAGTGTATTAGA
TTTATTGAATGCATTTGACTTTTTTTTTTTTATTCAATAAGCTTTGTTGAATGCATTTGACTTTCATTTGATTGAATTTG
TTTTAATGAATGCATTCTACTTTCATTAAATTCAAATAACTTTATTGAATGCGTTTCACTTTCATTTGATTCAACTAGC
TTTGTTTGAATGCATTTGACTTACATTTGATTCAATGACTTTCATTTGATTCAATTCGCGTTGTTTGAATGTATTAGA
CTTAGCTTTGTTTGAATGTATTTGACTTTTATTTCACTCGATTAGCTTTGTGAATGCATTTGACTTTCATTTGATTCAA
TTAGCTTTGTTCAATGCATTTGACTGTCATGTGATGCATTTGAGTTTTAATTTGATTCAATTAGCTTTGTCTGAATGT
GTTTGACTTTCATTTGATTCAACTAGCTTTCATGAATGCGATTAACTTTCATTTGATTCAATTAGCTTTGATTGAATG
TGTTTGACTTTCATTTGATCCAATTACCTTTGTTTAATGTGTTTGACTTTCATTAGATTTAATTAGCTTTGATGAATGC
ATTTGACTTTCATTTGATTTAATTTTCTTTGTTGAATGTGTTTGACTTTCATTTGATTCATATAGCTTTGTTAAATGCA
TTTGACTTTCATTTAGGTGAATTAGATTTGTTGAATGCATTTGACTTTCATTAGATTTAATTAGCTTTTTTGAATATAT
TTGACTTTCACAGGATTCAATTAGCTCTGGTGGAATGCATTTCACTTCCATTGGATTCAATAAGCCTTTTTGAAACA
ATTTGACTTTCATAGGATTCAATTAGCTTTGTTTTATTGTGTTTAACTTGCACTTGATTCAATAAGCATTGTTGAATG
CATTTGATGTTCATTTAATTCAATTAGCTTTGTTCAATACATCTCACTTTCTTTTGATTCAATTAGCTTTGTTATGAATT

TGTGTTTCATTTGATTCAATTATCTTTGTTCGAATGTTTTTGACTTTCTTTAGATTTAATCAGCTTTGTTGAATGCATT
TTGACTTTCATTTGATTCAATTAGCTTTATTGAATGCAATTGACCAATATTTGATTCAATTAACATACATTTTGATTCA
ATAAGCTTTGTTTGAATGTGGTTCACTTTCAGTTAAGAGAATTATATTTGTTGAATGCATTTGGCATTTTTTGATTCA
ATTAGCTTTGTTGAATGCATTTGACTTTCATTTGATTTAATTTGCTTTGTTTGAATGTGTTTGACTGTGATTTGATTCA
ATTAGCTTTGTTGAGTGCATTTCACTTTCATTTGATTCAGTTAGCTTTGTTTGAATGTGTTTGACTTTTATTTGATTCA
ATTACCTTTGTTTGAATGTGTTTAACTATCATTAGATTTAATTAGCTTTATTGAATGCATTTGTCTTTCATTTGATTTA
ATTATATTTGTTGAATGCATTTGACTTTTATTTGATACAATTAGCTTTGTTTGAATGTATTTGACTTTCATTTGATTCA
ATAAGCATTGTTGAATGCATTTGATGTTCATTTGATTCAATTAGGTTTGTTCAATGAATTTGATTTTCATTTGATTCA
ATAAGCATTATTGAATGCATTTGATTCAATAAGCTTTGCTCAATGCATTTGGCTTTCACTTGATTCAATTAGCTTTGT
TATGCATTTGAGTTTCATTTGATTCAATTATCTTTGTTCGAATGTGCTTGACTTTAATTTTATTCAATTAGCATTTTTT
GAATGTGTTTGACTTTCATTTAAGAGAATTAATTTGTTGCATGCATTTGACTTTTTTTTTTATTCAATTAGCTTTGTTG
AATGCATTTTACTTTCATTTGATCAAATTAGCTTTGTTGAATGTATCTGACTTTCGTTTAATTCAATTAGCTGTGTTGA
GTGTATTTCACTTCCATTTGATTCAATTAGCTTTGTTGGAATGCGTTTGACTCTCATTTAATTCAATTAGCTTTATTGA
ATGCATTTGACTTTTATTTGATTCAATTAACTTAGTTTGAATGTGTTTCACTTTCATTTAATTTAATTATCTTTGTTTGA
ATGTGTTTGACTTTCATTTCATACAATTAGCTTTATAAATGCATTTAACTTTCATTTGATTTAATTTGCTTTGTTGAAT
GTGTTTGACTTTCATTTGATTCATTTAGCTTTGTTAAATGCATTTGACTTTCATTTAAGTGTATTAGATTTATTGAATG
CATTTGACTTTTTTTTTTTTATTCAATAAGCTTTGTTGAATGCATTTGACTTTCATTTGATTGAATTTGTTTTAATGAAT
GCATTCTACTTTCATTAAATTCAATTAACTTTATTGAATGCGTTTCACTTTCATTTGATTCAACTAGCTTTGTTTGAAT
GCATTTGACTTACATTTGATTCAATGACTTTCATTTGATTCAATTCGCTTTGTTTGAATGTATTAGACTTTCATTTGAT
TAAATTAGCTTTGTTTGAATGTATTTGACTTTTATTTCACTCGATTAGCTTTGTGAATGCATTTGACTTTCATTTGATT
CAATTAGCTTTGTTCAATGCATTTGACTGTCATGTGATGCATTTGAGTTTTAATTTGATTCAATTAGCTTTGTCTGAA
TGTGTTTGACTTTCATTTGATTCAACTAGCTTTCATGAATGCGATTAACTTTCATTTGATTCAGTTAGCTTCGATTGA
ATGTGTTTGACTTTCATTTGATTCAACTAGCTTTTATGTAATGCGATTAACTTTCATTTGATTCAATTAGCTTTGATTG
ATTGTGATTGACTTTCATTTGATGTAT

Table of detected monomers

Indices	Period Size	Copy Number	Consensus Size	Percent Matches	Percent Indels	Score	A	C	G	T	Entropy (0-2)
17--2544	39	63.5	39	70	12	480	24	11	14	49	1.77
49--351	120	2.6	116	77	9	297	25	11	12	49	1.75
334--372	19	2.1	19	95	0	69	28	15	10	46	1.78

559--621	20	3.2	20	71	13	67	25	17	11	46	1.81
524--1734	39	30.3	39	74	9	604	24	11	15	48	1.78
524--1243	79	9.2	78	78	5	603	25	12	14	47	1.79
524--2548	118	17.1	117	76	8	1517	24	11	14	49	1.77
1306--1361	19	2.9	20	71	7	62	21	8	19	50	1.75
1330--1756	79	5.4	79	84	1	509	24	9	15	50	1.74
1644--1838	106	1.9	104	86	4	284	26	12	15	45	1.81
1722--1783	28	2.2	28	88	0	88	30	14	14	40	1.86
1753--1847	37	2.5	37	86	6	111	22	14	14	48	1.80
1750--2548	39	20.3	39	77	4	701	24	12	13	49	1.76
2529--2567	19	2.1	19	95	0	69	28	15	10	46	1.78
2491--2603	59	1.9	59	90	0	181	23	14	13	48	1.79
2548--2719	40	4.4	40	85	2	215	22	12	15	48	1.79
2734--2939	40	5.2	39	80	5	243	23	13	16	47	1.81
2734--2939	80	2.6	80	92	2	344	23	13	16	47	1.81
2768--2939	20	8.6	20	64	12	68	24	13	15	46	1.82

Consensus pattern (39 bp):

TTTGACTTTCATTTGATTTAATTTGCTTTGTTAAATGCA

Consensus pattern (116 bp):

AATGCATTTCACTTTCATTTGATTCAACTAGCTTTGTTGAATGCATTTGACTTTCATTTGATTCATTTGCTTTAATAAA

TGCATTCTACTTTCATTAAAGTCAAATAACTTTATTG

Consensus pattern (19 bp):

TGACTTACATTTGATTCAA

Consensus pattern (20 bp):

CTTTCATTTGATTCAATTAG

Consensus pattern (39 bp):

ATTTGATTCAATTAGCTTTGATGAATGTGTTTGACTTTC

Consensus pattern (78 bp):

ATTTGATTCAATTAGCTTTGTTGAATGTATTTGACTTTCATTTGATTCAATTAGCTTTGTTGAATGTATTTGACTTTC

Consensus pattern (117 bp):

ATTTGATTCAATTAGCTTTGTTGAATGTGTTTGACTTTCATTTGATTCAATTAGCTTTGATGAATGCATTTGACTTTCA

TTTGATTCAATTAGCTTTGTTGAATGTATTTGACTTTC

Consensus pattern (20 bp):

ATTTGTTGAATGCATTTGGC

Consensus pattern (79 bp):

TTTGATTCAATTAGCTTTGTTGAATGCATTTGACTTTCATTTGATTCAATTAGCTTTGTTTGAATGTATTTGACTTTCA

Consensus pattern (104 bp):

CATTTGATTCAATAAGCATTGCTCAATGCATTTGGATTTCACTTGATTCAATTAGCTTTGTTATGAATTTGAGTTTCA

TTTGATTCAATAAGCATTATTGAATG

Consensus pattern (28 bp):

CATTTGATTCAATAAGCATTACTCAATG

Consensus pattern (37 bp):

TTGATTCAATAAGCTTTGTAATGCATTTGAGTTTCAC

Consensus pattern (39 bp):

CATTTGATTCAATTAGCTTTGTTGAATGCATTTGACTTT

Consensus pattern (19 bp):

TGACTTACATTTGATTCAA

Consensus pattern (59 bp):

ACTTTCATTTGATTCAACTAGCTTTGTTTGAATGCATTAGACTTACATTTGATTCAATG

Consensus pattern (40 bp):

TGACTTTCATTTGATTCAATTAGCTTTGTTTGAATGTATT

Consensus pattern (39 bp):

ATTTGATTCAATTAGCTTTGTTGAATGTGATTGACTTTC

Consensus pattern (80 bp):

ATTTGATTCAATTAGCTTTGATTGAATGTGTTTGACTTTCATTTGATTCAACTAGCTTTCATGTA

ATGCGATTAACTTTC

Consensus pattern (20 bp):

ACTTTCATTTGATTCAATTA

BLAST alignments:

Chenopodium quinoa clone 12-13p repeat region sequence. Sequence ID: HM641822.1

Beta corolliflora minisatellite DNA, clone pBC1447 Sequence ID: AJ288880.1

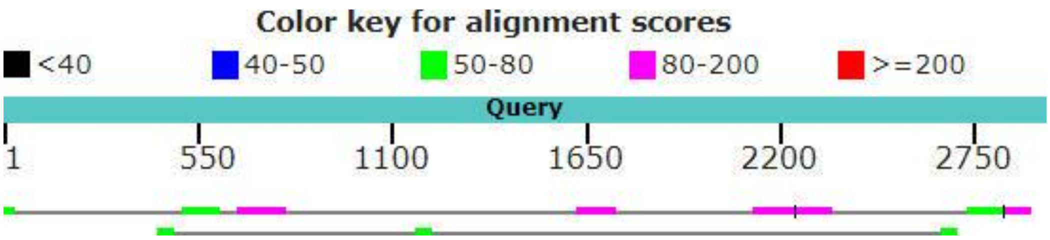

CLUSTER 134

>CL134Contig5 (2403-184.0-442166)

AAATTAAAAAAAAAAAGTCAATGCATTCGAGCAAAAGCTAATTGAATCAGATGAAAGTCAAACACAAACAAATCTA

ATGGAATCAAACGAAAGTCAAACACATTCTAACAAAGCTAATTGAATCAAATGAAAGTCAAATGCAATCAGCAAA

GCTAATTGAATCAAAAGAAAGTCAATTGCTTTCTATAAAGCTAATTGAACCAAATGAAAGTCAAATGCTTCCAACA

AAGCCAATTGAATCAAATGAAAGTAAAATGCATTCAACAAAGCTAAGTTCATCACATGAAACCCAAACGCATTTTA

CAAAGCTAAATGAATTAAATAAAAGTCAATGCATTCGAGCAAAGCTAATTGAATCAAATGAAAGTCAAACACATTC

TAACAAAGCTAATTGAATCAAATGAAAGTCAAAGACATTAAAACAATGCTAATTGAATCAATTAAAATTTAACTTC

ATTCATCATGGCTAATTCACTCAAATTTGACTCAAATGCAATTTACAAAGCTAAACAAATGAAATGAAAGTAAAAC

ACATCCATACAAAGCTAATTAAATGAAATGAAAGTTAAAAGCATTTAATCAGGCAAATTGAATCAAACCAATGTCA

AATGCATTTAAAAAAAGCTAATTAAATCAAATGAAAGTCAAACACGTTCAAAAAAAGATAATTAAATCAAATGAAA

CTCAAATGCATGTTACAAAGGTAAACGAATCGAATGAAAGTCAAACAAAGCTAATTGAATCAAATGAAAGTTAAA

TGCATTCAACAAGGCTACATCACTCAAATGAAAGTCAAATGCAATTTAGAAATCTAAATGAATGAAATGAAATTCA

AACACATTCAAACAAAGCTAGCTAAATGAAATGAAAGATTCAACAAAGCAAATTGAATCAAACCAAAGTCAAATTC

ATTCAAAAAAGCTAATTAATTCAAATGAAAGCCAAACACATTCAAAAAAAGCTAATTGAATCAAATGAAAGTCAAA

TACATTCAACAACGCTAAATTAATTGAATGAAATTCAAATACATTAAAGGTAAGCTACTTAAATCAATTAAAAGTCA

ACTGCATTCAAAAAAGCAAATCGAATCAAATGGAAGTCAAATGCATTCAACAAAGCTAATTAAAGCAAAAGAAAG

TCAAACACATTCAGAAGAAAAATCTAATTCAATCAAATGAAAGTCAAATGCATTTTCATAGCTAATTGAGCTGAAT

GAAAGTCAAACACATTCAAACAAAGCTAATTGAATCAAATGAAAGTCAAATGCATTCAACAAGGGTAATTCAATCG

AATGAACGTCAAATGCAATTCATAAAGCCAAATGAATCAAATAAAAGTCAAACACATTCAAACAAAGTGAAATGA

AAGTCAAATACATTCTACAAAAAAAACCGAATCGATTGAAGTCATTGCATTCAGCAAAGCTAACTGAATCAGACGA

AAGCCAATCACATTCAAACAAAGCCAATTTAATCAAATAAAAGTCAAATGCAATTAACAAAGCCAATTGAATCAAA

TAAAAGTCAAATGCTTTCAGAAATGTATACTAAAATCAAATGAAAGTGAAGAGCATTCAACAAAGCGAATTGAATC

AAATGAAAGTCTAACACATTAAATCAAAGCAAATTAAGTAAAATGAAAGTCAAATGCATTCAAACAAAGCTAATTG

AATCAAATGAAAGCCAAATGCAATCAGCAAAGCTAATTGAATCAAAAGAAAGTCAATTGCTTTCTATAAAGCTAAT
TGAACCAAATGAAAGTCAAATGCTTCCAACAAAGCCAATTGAATCAAATGAAAGTAAAATGCATTCAACAAAGCTA
AGTTCATCACATGAAACCCAAACGCATTTTACAAAGCTAAATGAATTAAATAAAAGTCAATGCATTCGAGCAAAGC
TAATTGAGTCAGATGAAAGTCAAACACATTCAAACAAATCTAATTGAATCAAACGAAAGTCAAACACATTCTAACA
AAGCTAATTGAATCAAATGAAAGTCAAAGACATTAAAACAATGCTAATTGAATCAATTAAAATTTAACTTCATTCAT
CATGGCTAATTCACTCAAATTTGACTCAAATGCAATTTACAAAGCTAAACAAATGAAATGAAAGTAAAACACATCC
ATACAAAGCTAATTAAATGAAATGAAAGTTAAAAGCATTTAATCAGGCAAATTGAATCAAACCAATGTCAAATGCA
TTAAATCAAAGCAAATTAAGTAAAATGAAAGTCAAATGCATTCAAACAAAGCTAATTGAATCAAATGAAAGCCAAA
TGCAATCAGCAAAGCTAATTGAATCAAAAAAAAAGTCAATTGCTTTTTATAAAGCTAATTGAACCAAATGAAAGTC
AAATGCTTCCAACAAAGCCAATTGAATCAAATGAAAGTAAAATGCATTCA

Indices	Period Size	Copy Number	Consensus Size	Percent Matches	Percent Indels	Score	A	C	G	T	Entropy (0-2)
13--2403	39	61.4	39	72	8	1484	49	16	12	22	1.78
13--2401	117	20.5	117	72	9	1136	49	16	12	22	1.78
13--2391	79	30.6	77	71	9	1234	49	16	12	22	1.78
697--753	30	1.9	30	85	0	78	52	12	17	17	1.74
877--916	21	2.0	20	85	5	53	52	20	7	20	1.70
583--1024	149	3.0	148	74	9	448	51	14	11	22	1.74

Consensus pattern (39 bp):

AAAGTCAAATGCATTCAACAAAGCTAATTGAATCAAATG

Consensus pattern (117 bp):

AAAGTCAAATGCATTCAACAAAGCTAATTGAATCAAATGAAAGTCAAACGCATTTTACAAAGCTAATTGAATCAAA
TGAAAGTCAAATGCATTCCAACAAAGCTAATTGAATCAAAT

Consensus pattern (77 bp):

AAAGTCAATGCATTCAACAAAGCTAATTGAATCAAATGAAAGTCAAACACATTCAACAAAGCTAATTGAATCAAAT
G

Consensus pattern (30 bp):

ACAAAGCTAAACGAATCAAATGAAAGTCAA

Consensus pattern (20 bp):

CAAAGCAAATTCAATCAAAC

Consensus pattern (148 bp):

GCAAATTGAATCAAACCAAAGTCAAATGCATTCAAAAAAGCTAATTAAATCAAATGAAAGTCAAACACATTCAAAA

AAAGCTAATTGAATCAAATGAAACTCAAATACATTCAACAAAGCTAAACTAATTGAATGAAAGTCAAACAAA

BLAST alignments:

Chenopodium quinoa clone 12-13p repeat region sequence. Sequence ID: HM641822.1

Beta corolliflora minisatellite DNA, clone pBC1447 Sequence ID: AJ288880.1

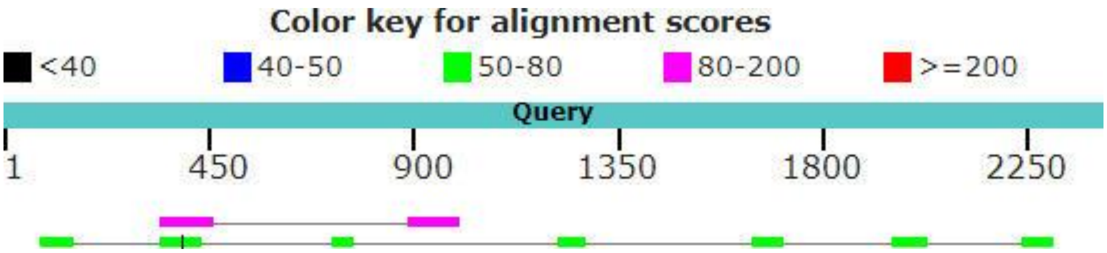

CLUSTER 144

>CL144Contig8 (2170-136.3-295855)

AAAAAAGCTACTTGAATCAAATGATAGTCTAATGAATTCAAACAAGCTATTTGAATCAATTAAAAGTGAAATGCAT

AAAAAAAAAAAAGCTAATTGAATCAAAAAAGAAAGTCAAACACATTCAAACAAAGCTAATTGAATCAAATGAAAA

TAAAACACGTTCAAACAAAGCTAATTGAATCGAATGAAAGTCAAATGCATTCAAACAAAGCTAATTGAATCAAATG

AAAGTCAAACCCATTCAAACAAAGCTAATTATATCAAATGAAAGTTAAAGACATTCAAACAAATCTAATTAAATCA

AAAGAATGTCAATTGCATTCAACAAAGCTAGTTTAATAAAATGAAAGTCAATTGCATTCAACAAACTTAATTAAAT

GAAATAAAAGTCAAACACATTCAAACAAAGCTAACTGAATGAAATGAATTCAAAAAAGCTAATTGAATCAATTAAA

AGTCAAATGAATTAAAACAAAGCTAATTCAATGAAATGAAAGTCAAACACATTCAAATAAAGCTAATTGAACCAAA

TGAAACTCAAACACATTCAAACAAAGCTAATTGAACCAAATGAAAGTCAAATGAATTCAAAAGCTAATTGAATCGT

TTAAAAGTCAAATGCTTTCACCAGAGCTAGTTGAATCAAAAAAGAAAGTCAAACACTTTCAAACACAGCCAATTGA

AACAAATGAGACTCAAATGAATTCAACAAAGCCAATTGAATCAAATGAAATTCAAATGCTTTCAACAAAGCTAATC

AAATCAATTGATAGTCAAATGCATTTAAACAAAGCTAATCGAAAGTCAAACACATTCAAACAGAGCTAGTTAAATC

AAAACAATGTCAATTGCATTCCACAAAGCTAATTGAATCAAATGAATTCAAAAAAGCTAATTAAATCAATTAAAAG

TCAAATGCATTCAAAAAAAGCTAATTGAATCAAATGAAAGTCAAACACATTCAAACAAAGCTAATTGAATCAAATA

AAAGTCAAACACAACCTAACAAAGCTAATTGAATCAAATAAAAGTGAAATGCATTCAGAGAAGCTATTTGAATAGT

TTGAAAGTCAAATGCATTCAAAGAAAGCTAATTGAATCAAATGGAAACAATCACATCCAAACAAAGCTAATTGAAT

CAAATGAAAGTCAAATGCATTCAAAAAAGCCGTTTGAATCAATTAAAAGTCAAATGCATTGAAAAAACCTATTTGG

ATCAACTGAGAGTCAAATGCATTCAAACGAGACTAATTGAATCAAACGAAAGTCAAACACATTCAAACAAAGCTAC

TTGAATCAAATGAAAGTCAAATGAATCCAAAAAAGCTAATTGAATAAATTGAAAGTCAAATGCGTTCAAGCAAAG

CTAGTTCTCAAAAGAAAGTCAAAACACATTAAAGCAAAGCTAATTGAATCAAATGAAAATCAAACACATTCAAACA

AAGCTAATTGAATCAAATGAAAGTCAAATGCATTCAAAAAGCTATTTGAATCAATTGAAAGTCAAATGCTTTCAAA

AAAGCTATTTGGATCAATTGAAAGTCAAACACACTCAAACAAAGCTAAAGAATAGAATACATGTCAAACGCATTCA

AAAAAAGCTATTTGAATCAATTGAAAGTCAAATGCATTCAATAAAGCTATTTGGATCAATTAAAAGTCAAATGCAT

TCAAACGAGACTAATTGAATCGAATGAAAGTCAAACACATTCAAAAAAAGCTACTTGAATCAAATGATAGTCTAAT

GAATTCAAACAAGCTATTTGAATCAATTAAAAGTGAAATGCATAAAAAAAAAAAGCTAATTGAATCAAAAAAGAA

AGTCAAACACATTCAAACAAAGCTAATTGAATCAAATGAAAATAAAACACGTTCAAACAAAGCTAATTGAATCGAA

TGAAAGTCAAATGCATTCAAACAAAGCTAATTGAATCAAATGAAAATAAAACACGTTCAAACAAAGCTAATTGAAT

CGAATGGAAGGCAAATGCATTCAAACAAAGCTAATTGAATCAAATGAAAGTCAAACCCATTCAAACAAAGCTAATT

ATATCAAATGAAAGTTAAAGACATTCAAACAAATCTAATTAAATCAAAAGAATGTCAATTGCATTCAACAAAGATA

AATGAATCAAATTAAATTCAAACACATCCAAACAAAGCTAGTAGA

Indices	Period Size	Copy Number	Consensus Size	Percent Matches	Percent Indels	Score	A	C	G	T	Entropy (0-2)
86--2165	40	53.2	40	76	8	1586	50	15	11	22	1.77
1293--1353	20	3.1	20	70	25	58	52	9	14	22	1.71
1611--1674	19	3.2	20	66	20	53	43	14	12	29	1.81

Consensus pattern (40 bp):

AAAGCTAATTGAATCAAATGAAAGTCAAACACATTCAAAC

Consensus pattern (20 bp):

TGAATCAAATGAAAGTCAAA

Consensus pattern (20 bp):

TCAATTAAAGTCAAATGCAT

BLAST alignments:

Chenopodium quinoa clone 12-13p repeat region sequence. Sequence ID: HM641822.1

Beta corolliflora minisatellite DNA, clone pBC1447 Sequence ID: AJ288880.1

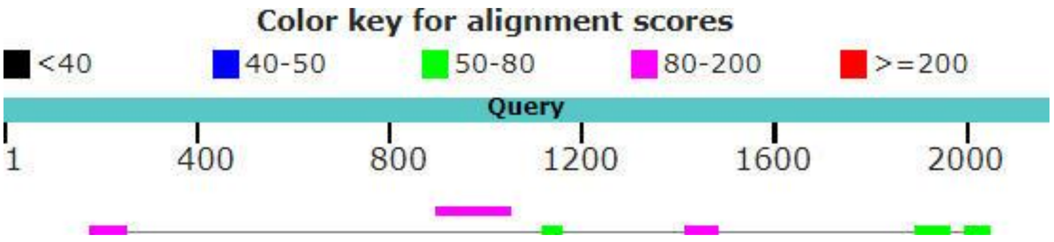

CLUSTER 145

>CL145Contig8 (3578-52.4-187313)

TCAAATGAAAGCCATACACATTTTAAAAAAGCTAATTGAATCAAATGAAATTCAAAAACATTACCGATAAAGCTAA

TTGAAAAAAATGAAATTCAAATGCATTCAACAAATCTAATTTAATCAAATGAAATTCAAACACATTCAACAAAGCTA

ATTGAATCAAATGAGAGTCAAATGCATTCAACAAAGCTAATTGAATTAAATGAAAGTCAAATGCATTTAACATTGC

TAAATGAGTCAAATGCAAGACAAATGCTTTTAGCAAAGCTAATTGAATCAAATGAAAGTCAAAAACATTCTAGCAA

AGCTAATTGAGTCAACTAAAAGTCAAACACATTCAAACAACGCTAATCAAATCAAATGAAACTCAAATGCATTCAA

CAATGCTAATTAAATCAAATGAAAGTCAAACACATTCTAACAAAGCTAATTGAATCAAATGAAAGTCAAATGCATT

CAACAAAGCTAATTTATTCAAATGAAAGTCAAATGCGTTCAATAATGTTAATTGAATCTAATGCAAGTCAAATGTAT

ACAATAAAGCTAATTAAATCAAAAGAGAGTCAAATGCATTCAACAAAGCTAATTGAATAAAATGAAAGTCAAATG

CATTCAACAATGCTAATTGAATCATTTGCAAGTCAAACAAACTAATTGAATCAAACGAAAGTCAAATGCATTCACTA

ATGCTAATTGAATCAAATAAAATTCAAATGTTTAGCTAATTGAGTCAAAAGAAAGTCAAATGCATTCAAACAAATC

TAATAAAAGCAAATGAAAGTCAAATGCATTTAACAAATCTAATTGAATCAAATGAAAATCAAATTTATTCAATGAA

GGTAATTAAAAGAAATTAAATTCAAACACATTCTAACAAAGCTAGTTGAATCAAATGCGAATCAAATGTGTTCAAA

CAAAGCTAATTGAATCAAACGAAATTCGAACACATTCAACAAAGCTAATTGAATCAAATGCAAATCAAATGTATTC

AAACAAAGCTAATTGAATCAAATGAAATTCAAACACATTCAACAAAGCTAATTGTATCCAACGAAATTCAAACATA

ATCAACAAAGCTAATTGAATCAAATGAAAATCAAATGCATGCAACAAATTTAATTGAATAAAATGAAAGTCAAATG

TATTCAATAATGTTAATTGAATCAAATGCAAGTCAAATGCATTTGACAAGCTAATTGAGTCAAATGAAAGTCAAAA

ACATTCTAAAAAGGCTAATTAAATCAAACGAAACTCAAACACATTCTACAAAGCTAATTGAGTCAAATGAAAGTCT

AACACATTTAAACAAAGCTAATTGAATCAAGTGAAACTCAAATGCATTCGATAAAGCTAATTGAAAAAAATGAAAT

TCAAATGCATTCAACAAATCTAATTTAATCAAATGAAATTCAAATACATTCTAACAAAGCTAGTTGAGTCAAATAAA

AGTCAAATACATTCTAACAAAGCTAGTTGAGTCAAATAAAAGTCAAACACATTAATCAACAAAGCTAATTAAATCA

TATGAAACTCAAATGCGTTCAATAAAGCTAATTAAATTAAATGAAAGTCAAAAACATTCTAGCAAGGCTAATTAAG

TCAACTAAAAGTCAAACACATTCAAACACATTCAAACATTGCTAATCAAATCAAATGAAACTCAAATGCATTCAACA

AAGCTAATTAAATCAAATGAAAGTCAAACACATTCTAACAAAGCTAATTGAATCCAATAAAAGTCAAATGCATTCA

GTAAAGCTAACTGAATCAAATGAAAGTCAATTGCATTAAAACAAAGCAAGTTGAATGAAATGAAAGTCCAATGCA

TTCAATAAAGATAATTGAGTTAAATGAAAGTAAAACACATTCAGACAAAGCTAGTTGAATCAAATGAAAGTCTAAT
GCATTCAATAAAGCTAAGTGAATCAAATGAAAGTCAATCACATTAATACAAAGCTAATTAAAACAAGTGAAAGTCA
AAAGCATTTAAAAAAGCTAATTGAAATAAATTAATGTAAAATGTATTCAACCAAAGCTAATTGAATCAAATGAAAG
TCAAACACATTCTAATAAAGCTAATTGAATCAAATGAAACTCAAATGCATTCAACAAAGCTAATTGAATCAAATGA
AAGTCAAATGCATTCAACTAAGCTAATTGAATCAAATGAAAGTCTAATGCATTCAATAAAGCTAAGTGAATCAAAT
GAAAGTCAATCATATTAATACAAAGCTAATTGAATGAAATGAAAGTCAAATGCTTTCAACAAAGCTAATTGAATCA
AATGAAAGTCAAACACATTCTCACAAAGCTAATTGAATCCAATAAAAGTCAAATACATTCAGTAAAGCTAACTGAA
TCAAATGAAAGTCAATCGCATTAAAACAAAGCTAGTTGAATGAAATGAAAGTCCAATGCATTCAAGAAAGATAATT
GAATCAAATGAAAGTAAAACACATTCAAACAAAGCTAGTTGAATCAAATGAAAGTCCAATGCATTCGATAAAGCTA
ATTGAATAAAATTCAAGTGAATTTCATTCGACAAGCTAATTGAACCAACTGAAAGTCAAACACATTCTAACAAAAA
CAAATCAATCAAATGATACTCAAATTCATTTAACTAAGCAACTTGAATCAAATGAAAGTCAAATGCATTCAAATAAG
CTAATTGAATCAAATGAAACTAAAATGCATTTGACAAACAAAATTGAATCAAATTAAAGTCAAATACATTCAAACA
AAGCTATTTGAATCTACTGAAACTCAAAATAAATTCAACAAAGCTAATTGAAACAAATGAAAGTCAAAACATATTC
ACACAAAGCTAATTGAATTAAATGAAACTCAAATGCATTCAACAAATCTAATTGAATCAAATGAAAGTCAAATGCA
TTCAACTAAGCTAATTGAAAGAAAAGAAAGTCTAATGCATTCAACAAAGCTAATTAAATCAAATGCAAGTCAAATG
CATTCAAAAAAGCTAATTGAATCCAATGAAAGTCAAAATCATTCTTAAAAAGCTAATTGAATCAAATGAAACTGAA
GCACATTCTAACAAAGTTGATTGAGTCCAATGAAAATCAAACACATTCAAACAAAGCTAATTGAATCAACTAAAAC
TCAAATGCATTTAACTAAGCAAATTGAATCAAATGAAAGTCAAATGCATTCAACTAAGCTATTTGAATCAAATGAA
AGTCTAATGCATTCAATAAAGCTAAGTGAATCAAATGAAAGTCAATCACATTAATACAAAGCTAATTAAAACAAGT
GAAAGTCAAAAGCATTTAAAAAAGCTAATTGAAATAAATTAATGTAAAATGTATTCAACCAAAGCTAATTGAATCA
AATGAAAGTCAAACACATTTTAATAAAGCTAATTGAATCAAATGGAACTCAAATGCATTCAACAAAGCTAATTGAA
ATCTAATGAAAGTCAAATGCATTCAACTATGCTAATTGAAAGAAAAAAAAGTCTAATGCATTCAACAAAGCTAATT
AAATCAAATGCAAGTCAAATGCATTCAAAAAAGTTAATTGAATCCAATGAAAGCAAACACATTCTTAAAAAGCTAA
TT

Table of detected monomers

Indices	Period Size	Copy Number	Consensus Size	Percent Matches	Percent Indels	Score	A	C	G	T	Entropy (0-2)
28--3557	39	89.7	39	78	5	3103	48	15	11	24	1.77
1--3578	79	45.5	78	77	7	2752	48	15	11	24	1.77
436--499	21	3.2	21	67	13	62	45	15	12	26	1.82

572--754	68	2.7	67	82	7	199	46	14	12	26	1.80
1611--1637	10	2.7	10	100	0	54	51	29	0	18	1.46
1511--3031	118	12.7	129	72	13	434	48	15	11	24	1.77
1550--1716	89	1.9	90	84	1	228	49	18	8	23	1.74

Consensus pattern (39 bp):

AAAGCTAATTGAATCAAATGAAAGTCAAATGCATTCAAC

Consensus pattern (78 bp):

TCAAATGAAAGTCAAATACATTCAACAAAGCTAATTGAATCAAATGAAAGTCAAATGCATTCAACAAAGCTAATTG
AA

Consensus pattern (21 bp):

TCAAATGAAAGTCAAATGCAT

Consensus pattern (67 bp):

ATTCAAACAAACTAATTGAATCAAAAGAAAGTCAAATGCATTCACAATGCTAATTGAATCAAATAAA

Consensus pattern (10 bp):

TCAAACACAT

Consensus pattern (129 bp):

GCTAATCAAATCAAATGAAACTCAAATGCATTCAACAAAGCTAATTAAATCAAATGAAAGTCAAAAACATTCTAAC
AAAGCTAATTGAATCCAATAAAAGTCAAACACATTCAAACACATTCAAACATT

Consensus pattern (90 bp):

GCTAATCAAATCAAATGAAACTCAAAAACATTCTAACAAAGCTAATTAAATCAAATAAAAGTCAA
ACACATTCAAACACATTCAAACATT

BLAST alignments:

Chenopodium quinoa clone 12-13p repeat region sequence. Sequence ID: HM641822.1

Beta corolliflora minisatellite DNA, clone pBC1447 Sequence ID: AJ288880.1

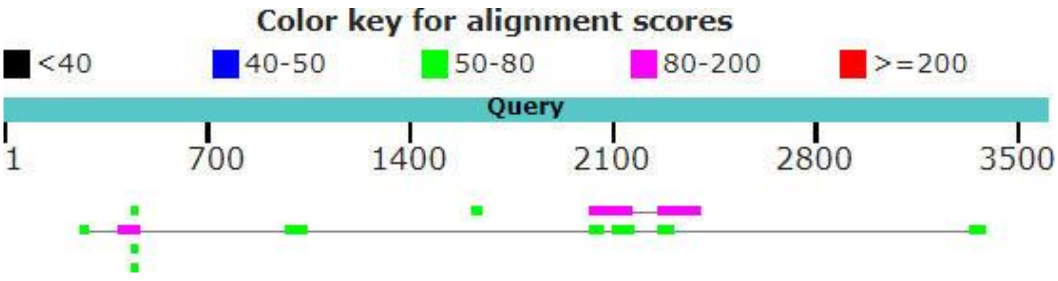

CLUSTER 253

>CL253Contig2 (3841-24.2-93112)

ATGCATTCGACAAAGTTATTTGAATCAAATGAAGGTCAAAGGCTTTTAACAATGCAAATTGAATCAAATAATATTC
AAATGCATTTAACAAAGCTAATTGAATCAAATGACAGTCAAATGCATATACGTAAGCTAGTTCATTCAAATGAAAG
TCAAGTGGATTAAACAAAGTTAAATAAACCTAATGACAGTCAAAAACTTTCAAACAAACCTTATTAAATCAAATGA
AAATCAATCACATTTAAACAAAGCTAATTAAGCCAAATAAAAGTCAAACACATCCACACAAAGCTACATGAATCAA
ATGAAAGTGAAATGCATTCTACAAAGCTAATTAAATCAAATAAAAGTTAAATGCGTTAAACAAAGCTAATTGAATC
CAATGAAAGCAAATGCAATCAAACAAAGCTAATTGAATCCAATAAAACTAAAATGCTTCCAACAAAGCTAATTGAA
TCAAATATAAGTAAAGAACATTTAACAAACCTAATTGAACCAAAAGAAAATCAAACACATCCAAGCAAAGTTAATT
GAATTAAATAAAAGTCAAATGCATTCAACAAAGTTAGTTGAATCAAATGAAGGTCACATGCTTTAAACAACGCTAA
TTTAATCAAATGATATTCAAATGCATTCAACAAAGCTAATTGAATCAAATGACTGTCAAATGCATTTACGTAAGCTA
ATTCACTCAAATGAAAGTCAAGTAAATTAAACAAAGCTAACTGAATCAAATGAAAGTCAAATACATCCACACAAAG
CTAAATTAATCAAGTGAAAATAAAACACATTCAAACAAAGCTCACTGAATCAAATGAAAGTAAAATGCATTCAACA
AAGCTAATCGAATCAAATGAAAGTCAAACACATCCACACAAAGTTTAATAAATCAAATAAAAATCAAACACATTTA
AACAAAGCTAATTGAATCAAGTGAAAGTCAAACACAATCCCAAAAAGCTAAATGAATCATATGAAAGTCAAATGTT
TTCAACAAAGATAATTGAATCAAATGAAATTCAAATGCATTCAACAAATCTAATTGAGTCGAATAAAAGTCAAGCA
CATCCAATGAAATCTTAATGAATCAAATGAAAATCAAATGTATTCAACAAATTTAATTAAGTCAAATGAAAGTAAAT
TGCTTTCAACAAAGCTAATTGAATTAAATGAAAGTCAAATGGATTCAATAAAGCAATTGAATCAAATGAAAATCAA
ACACGTTCAAACAAAGCTAATTGTATCAAATAAATTTCAAACACTTCCAACAAAGCTAATTGAATCAAATGAAAGT
AAAATGCGCTCAAAATCTATTTGAATTAATTGAAAATCAATCATATTAATATATAGAGAATTGAATCAAATAGAAAT
CAAATGCATTAAACAAAGTTAGTTGAACCAAATGAAGATCAAATGTTTTCACAAAGCTAATAGAATAAAATGATAT
TCAAATGCATTCAACAAGGCAAATTGAATCAAATGGAAGTGAAATCCATTTAACATAGCTAATTAACGTAAACGAA
ATTCAAATGCAAATAATAAAGCTAATTGAATCAAATGAAAGTCAAATGCATTTAACAAAGCTAACTGAATCAAATG
GAAGCCAAATGTTTTCGACAAAGCTAATGGAATCAAATGAAAGTGAAATGCATTCAACAAAGCTAATTGAACCAA
ATGAAAGTCAAATGCGTTAAAAAAAGCTAATTGAATCAAATGAAAGAAAATGCATTCAAGCAAAGCTAATTGAAT
CAAATAAAATTAAAATGCTTCCAACAAAGCGAATTGAGTCAAAAGAAAATCAAACACATCCAAACAAAGCTATGA
ATGAAATAGAGATTAAATGCAGTCAACAAAGTTATTTGAATCAAATGAAGGTCAAAGGCTTTTAACAATGCTAATT
GAATCAAATGATATTCAAATTCATTTAACAAAGCTAATTGAATCAAATGACAGTCAAATGCATATACGTAAGCTAG
TTCACTCAAATGAAAGTCAAGTGCATTAAACAAAGCTAAATAAACCTAATGACAGGCAAAAACTATCAAACAAACC
TTATTGAATCAAATGACAATCAATCACATTCAAACAAAGCTAATTGAATAAAATAAAAATTAAATGCATTAAACAAA
GTTAGTTAAATCAAATAAAGATCAAATGTTTTTAACCAAGCAGATTGAATCAAATGATAGTCAAATGCATTCTACAA

AGAAAATTGAATCAAGTAAAAGTAAAATGCATTTTACAAAGCTAATACACTTAAATGAAAGTAAAAAGCATTTAAC
ATAGCTAATTAAATCAAATGAAAGTCAAATGCATTCGACAAAGCTAACTGAATCTAATTAGGGTAAAACACATCTA
AACAAAGCTAATTGTATCAAATGCATTCAACAAAGTTAATTGAATCAAATGAAAGTGAAATGCATTCAAACAAACC
TAATTGAATCCAATGAAATTCAAACAAATTCAAACAAAGCTTATTGAATTAAATGAAAGTCAAATGCATTCGGAAA
AGTTAATTGATTCAAATAAAAGTGAGATGCATTCAACAAATGTAATTGAATTAACCGAAGGTCAAACACATTCTAA
CACAGCTTACTGAATCAAATGAAAGTCCAACACATTCAAACAAAGCTAATAGAATCAAATGACAATCAAATGAATT
CAACAAAGAAAATTCAGTCTAATGTAAGTCAAATGCATTAAAAAAAAGCTAAATGAATTCAATGATAGTCAAACAC
ATTCAAACAATGCTATTTGAATCAAATAAAACTCAAACGCTTCCAACAAAGCTAATAGAATCAAATGAAAGTAAAA
TGCGCTCAAAAACTATTTGGATCAAATGAAAATCAAATGCATTGAACAAAGTTAGTTGAATCAAATGATGGTCAAA
TGCTTTTCACAAAGTTAATTAAATAAAATGAAAGTGAAATGTATTTAACAAAGCTAATTAAATCAAATAAAAGTCA
AATGCATTCAACAAAGCTAAATAAATCAAATAAAAGTAAAACGCATTTAAAGAAAGCTAATTAAATCAAATAAAAG
TGAAATGTTTTCGACAAAGCTAACTGAATTAAATAAAAGTGAAATGCGTTTAGCAAAGCTAATTGAATGAAATAAA
AGTCAGATGCATTCAACAAAGCTAGTTAAATCAAATGAAAGTCAAACACATTCAAACAAAGCTAATAGAGTCAAAT
AAAAGTCAAATGTATTTAAGAAATCTAATTAAATCAAATGATAGTCAAAAACATTCAAACAAAGATAATTTAATCA
AAAGAAATTTAAATGCATTCAACACATCTAATTCAACCAAATGAAAATCAAACAGATTCAAACAAATCTAATTGAAT
CAAATAAAATTAAAATGCTTCCAACAAAGCTAATTGAATCAAATGAAAGTCAAATGCATATACGTAAGCTAGTTCA
CTCAAATGAAAGTCAAGTGCATTAAACAAAGCTAAATAAACCTAATGACTGTAAAAAAACTTTCAAAAAAACCTTA
TTAAATCAAATGAAAATCAATCACATTTAAACAAAGCTAATTAAGCCAAATAAAAGTCAAACACATCCACACAAAG
CTACATGAATCAAATGAAAGTGAAATGCATTCAACAAAGATAATTAAATCAAATGAAAGTCAAATGCATTCAACAA
AGCTATTTGAGTCGAATAAATGTCAAGCACATCTAATAAAAGCTTAATGAATCAAATAAAAATCAAACACATTCAA
ATTAAGCTAATTGAATCAAATGAAAGTTAAATGCATTCAACAAAGCTAATTGAATCAAATGAAAGTCAACCACATC
CACACAAAGCTAAATGAATCAAATAAAAGGCAAATATA

Table of detected monomers

Indices	Period Size	Copy Number	Consensus Size	Percent Matches	Percent Indels	Score	A	C	G	T	Entropy (0-2)
1--3838	39	98.1	39	72	7	2113	49	14	11	24	1.77
162--281	40	3.0	40	73	8	100	53	17	5	23	1.65
1--3838	78	49.0	78	73	7	2408	49	14	11	24	1.77
60--3838	235	16.1	235	71	9	1017	49	14	11	24	1.77
2375--2816	158	2.8	155	77	8	399	48	15	11	23	1.78

Consensus pattern (39 bp):

ATGCATTTAACAAAGCTAATTGAATCAAATGAAAGTCAA

Consensus pattern (40 bp):

TTAAACAAACCTAATTAAACCAAATAAAAGTCAAACACAT

Consensus pattern (78 bp):

ATGCATTCAACAAAGCTAATTGAATCAAATGAAAGTCAAATGCATTCAACAAAGCTAATTGAATCAAATGAAAGTC
AA

Consensus 235:

TGAATCAAATAAAAGTCAAATGCATTCAACAAAGCTAATTAAATCAAATAAAAGTCAAATGCGTTAAACAAAGCTA
ATTGAATCAAATGAAAGCAAATGCAATCAAACAAAGCTAATTGAATCAAATAAAACTAAAATGCTTCAACAAAGCT
AATTCAATCAAATGAAAGTAAAGTAACATTTAACAAAGCTAATTGAACCAAAAGAAAGTCAAACACATCCACACAA
AGCTACA

Consensus pattern (155 bp):

TCAAATGCATTCAACAAAGCTAATTGAATTAATGAAGTCAAACACATTCAAACAAGCTTATTGAA
TCAAATGAAACTCAAACACATTCAAACAAAGCTAATAGAATCAAATGAAAGTCAAATGAATTCGA
AAAAGAAAATTCAGTCAAATAAAAG

BLAST alignments:

Beta corolliflora minisatellite DNA, clone pBC1447 Sequence ID: AJ288880.1

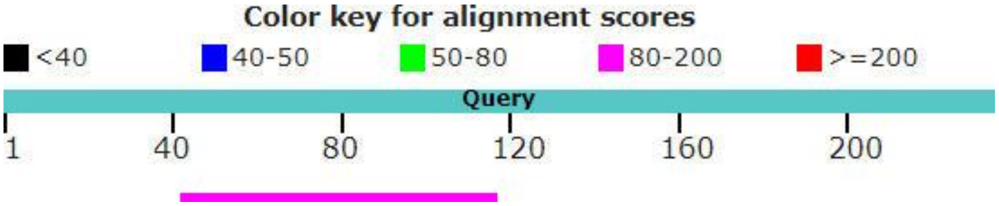

Supplement: Supplementary file 1 [file ijms-20-01201-s001.zip › suppl_Data-1.pdf]
