# Supplementary material for: Natural History of a Satellite DNA Family: From the Ancestral Genome Component to Species-Specific Sequences, Concerted and Non-Concerted Evolution
Source: Int J Mol Sci. 2019 Mar 9;20(5):1201. doi: 10.3390/ijms20051201 (PMC6429384; doi:10.3390/ijms20051201)
Supplement: Supplementary file 1 [file ijms-20-01201-s001.zip › suppl_Data-2.pdf]

Supplementary Data 2

Reconstruction of the major part of the ancestral monomer

BLAST-detected relatedness between minisatellite monomers of CficCl-61-40 satDNA family makes it possible to determine the major part of the ancient monomer. For this reconstruction for one RE cluster of C. bryobifolium (cluster 2) and for seven RE clusters C. vulvaria (clusters 28, 94, 112, 134, 144, 145 and 253) that show relatedness to C. quinoa and B. corolliflora minisatellites BLAST alignment was performed. DNA fragments with 100% matches, in combination, formed the most conservative fragment of the basic monomer.

Results

Tandem Repeat Finder results for detection of basic monomer in *B. corolliflora* GenBank

ID: AJ288880.1

gatcaaattaaagtaaaataaagtcaaacaaagctaattgaatcaaatgaaagtcaaatgaagtcaaacatagctaatagatc

Indices	Period Size	Copy Number	Consensus Size	Percent Matches	Percent Indels	Score	A	C	G	T	Entropy (0-2)
2--78	40	1.9	40	89	0	118	54	11	11	22	1.68

```
Indices: 2--78  Score: 118
    Period size: 40  Copynumber: 1.9  Consensus size: 40

          1 G

                    *
          2 ATCAAATTAAAGTAAAATAAAGTCAAACAAAGCTAATTGA
          1 ATCAAATGAAAGTAAAATAAAGTCAAACAAAGCTAATTGA

                          *    *          *
         42 ATCAAATGAAAGTCAAATGAAGTCAAACATAGCTAAT
          1 ATCAAATGAAAGTAAAATAAAGTCAAACAAAGCTAAT

         79 AGATC


Statistics
Matches: 33,  Mismatches: 4, Indels: 0
        0.89            0.11        0.00

Matches are distributed among these distances:
  40   33  1.00

ACGTcount: A:0.55, C:0.12, G:0.12, T:0.22


Consensus pattern (40 bp):
ATCAAATGAAAGTAAAATAAAGTCAAACAAAGCTAATTGA
```

Alignment:

```
B. corolliflora

                       Monomer 1                         Monomer 2

       +--------------------------------------++-----------------------------------+

      GATCAAATTAAAGTAAAATAAAGTCAAACAAAGCTAATTGAATCAAATGAAAGTCAAATGAAGTCAAACATAGCTAATAGATC

C. bryonifolium
Cl-2:                        TCAAACAAAGCTAATTGAATCAAATGAAAGTCAAA
                                AACAAAGCTAATTGAATCAAATGAAAGTCAAA
                                 ACAAAGCTAATTGAATCAAATGAAAGTCAAA
                                 ACAAAGCTAATTGAATCAAATGAAAGTCAAATG
C. vulvaria
Cl-28:                          AACAAAGCTAATTGAATCAAATGAAAGTCAAA
                                     AGCTAATTGAATCAAATGAAAGTCAAATG
                             TCAAACAAAGCTAATTGAATCAAATGAAAGTCA
Cl-94:                          AACAAAGCTAATTGAATCAAATGAAAGTCAAATG
                                   AAAGCTAATTGAATCAAATGAAAGTCAAA
                                AACAAAGCTAATTGAATCAAATGAAAGTCAAA
Cl-112:                         AACAAAGCTAATTGAATCAAATGAAAGTCAAATG
                                   AAAGCTAATTGAATCAAATGAAAGTCAAA
Cl-134:                         AACAAAGCTAATTGAATCAAATGAAAGTCAAA
                             TCAAACAAAGCTAATTGAATCAAATGAAAGTCAAATG
                                AACAAAGCTAATTGAATCAAATGAAAGTCAAATG
                             TCAAACAAAGCTAATTGAATCAAATGAAAG
                                  CAAAGCTAATTGAATCAAATGAAAGTCAAA
Cl-144:                      TCAAACAAAGCTAATTGAATCAAATGAAAGTCAAA
                             TCAAACAAAGCTAATTGAATCAAATGAAAGTCAAATG
                              CAAACAAAGCTAATTGAATCAAATGAAAGTCAAATG
Cl-145:                         AACAAAGCTAATTGAATCAAATGAAAGTCAAATG
                                  CAAAGCTAATTGAATCAAATGAAAGTCAAA
                             TCAAACAAAGCTAATTGAATCAAATGAAA
                                AACAAAGCTAATTGAATCAAATGAAAGTCAAA
Cl-253:                         AACAAAGCTAATTGAATCAAATGAAAGTCAAATG
                                AACAAAGCTAATTGAATCAAATGAAAGTCAA
                                   AAAGCTAATTGAATCAAATGAAAGTCAAATG
                                AACAAAGCTAATTGAATCAAATGAAAGT
Ancestral monomer:           TCAAACAAAGCTAATTGAATCAAATGAAAGTCAAATG
```
