# Supplementary material for: Natural History of a Satellite DNA Family: From the Ancestral Genome Component to Species-Specific Sequences, Concerted and Non-Concerted Evolution
Source: Int J Mol Sci. 2019 Mar 9;20(5):1201. doi: 10.3390/ijms20051201 (PMC6429384; doi:10.3390/ijms20051201)
Supplement: Supplementary file 1 [file ijms-20-01201-s001.zip › suppl_Data-3.pdf]

Supplementary data 3.

Repetitive elements selected for sequence characterization and in situ hybridization. Information on the source Chenopodium species, primer sequences used for amplification, annealing temperature (Ta) and number of sequenced clones is provided.

Repeat	Species	Primers	Ta (°C)	No of clones
CficCl-61-40	*C. ficifolium*	F: TCAAACAAAGCTTTTTGAATC<br>R: TTGTTTGAATGTGTTTGACTTT	50º	10
Proposed HOR units				
CacuCl-1-117	*C. acuminatum*	F: TCACGAAGCTTTGTTTGAATG<br>R: TGATTCAAATGAATGTCAAATGC	50º	8
CvulCl-28-118	*C. vulvaria*	F: TCAAATGAAAGTCAAAATCATTCA<br>R: TCATTTGATTCGATTAGCTTTGT	60°	5
CvulCl-28-397	*C. vulvaria*	F: TCAAGGACATTCAACCAAAGC<br>R: TGAACGCATTTGACTTTCAGTT	56°	5
CvulCl-112-117	*C. vulvaria*	F: CAATTAGCTTTGTTGAATGTGTTTG<br>R: TGAATCAAATGAAAGTCAAATGC	62°	5
CvulCl-134-117	*C. vulvaria*	F: TTGAATCAAATGAAAGTCAAACG<br>R: TCAATTAGCTTTGTTGGAATGC	60°	3
Cvul-145-129	*C. vulvaria*	F: CAAATCAAATGAAACTCAAATGC<br>R: TGTTTGAATGTGTTTGAATGTGTT	56°	9
