# Supplementary material for: Natural History of a Satellite DNA Family: From the Ancestral Genome Component to Species-Specific Sequences, Concerted and Non-Concerted Evolution
Source: Int J Mol Sci. 2019 Mar 9;20(5):1201. doi: 10.3390/ijms20051201 (PMC6429384; doi:10.3390/ijms20051201)
Supplement: Supplementary file 1 [file ijms-20-01201-s001.zip › suppl_Data-4.pdf]

Supplementary Data 4

Pairwise comparison of sequence variation within the CficCl-61-40 (A, B), and proposed HOR units CacuCl-1-117 (C, D), CvulCl-28-118 (E, F), CvulCl-28-397 (G, H), CvulCl-112-117 (I, J), CvulCl-134-117 (K, L) and Cvul-145-129 (M, N). For these monomers the sequence alignments containing the consensus sequence reconstructed by RE and sequences obtained by cloning and sequencing of clones are presented. For the cloned sequences identities with the consensus is represented by dots, differences by the alternative nucleotide characters. Tables contain the % of sequence similarities between the different sequences.

A

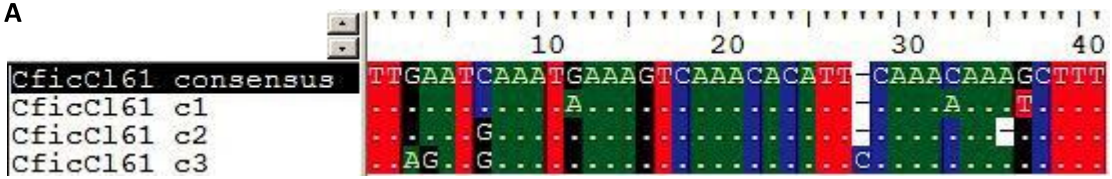

B

clones	CficCl61_consensus	CficCl61_c1	CficCl61_c2	CficiCl61_c3
CficCl61_consensus		92.5	95	90.2
CficCl61_c1	92.5		87.5	82.9
CficCl61_c2	95	87.5		90.2
CficiCl61_c3	90.2	82.9	90.2	

C

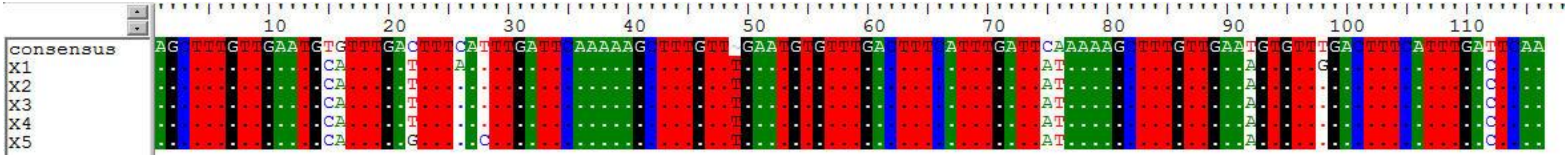

D

clones	CacuCl1_consensus	X1	X2	X3	X4	X5
CacuCl1_consensus		91.3	93.1	93.1	93.1	92.2
X1	91.3		98.2	98.2	98.2	96.5
X2	93.1	98.2		100.0	100.0	98.2
X3	93.1	98.2	100.0		100.0	98.2
X4	93.1	98.2	100.0	100.0		98.2
X5	92.2	96.5	98.2	98.2	98.2	

E

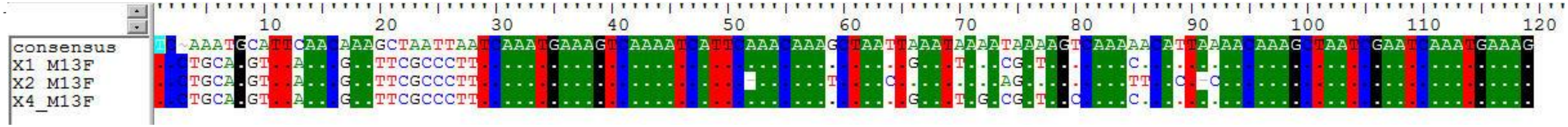

F

clones	CvulCl-28-118 consensus	X1_M13F	X2_M13F	X4_M13F
CvulCl-28-118 consensus		79.8	76.4	78.1
X1_M13F	79.8		89.9	98.3
X2_M13F	76.4	89.9		88.2
X4_M13F	78.1	98.3	88.2	

G

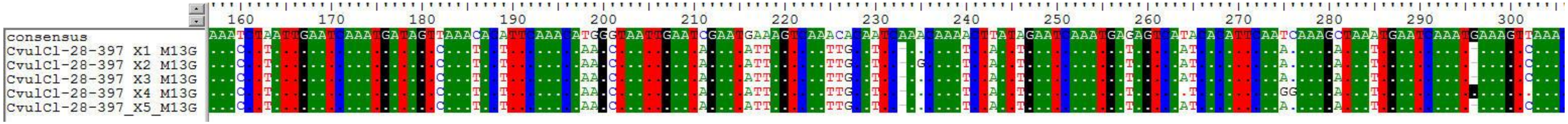

H

clones	CvulCl-28-397_consensus	X1_M13G	X2_M13G	X3_M13G	X4_M13G	X5_M13G
CvulCl-28-397_consensus		68.9	68.6	68.9	69.6	68.9
X1_M13G	68,9		99.7	100.0	98.3	100.0
X2_M13G	68,6	99.7		99.7	98.1	99.7
X3_M13G	68,9	100.0	99.7		98.3	100.0
X4_M13G	69,6	98.3	98.1	98.3		98.3
X5_M13G	69,9	100.0	99.7	100.0	98,3	

I

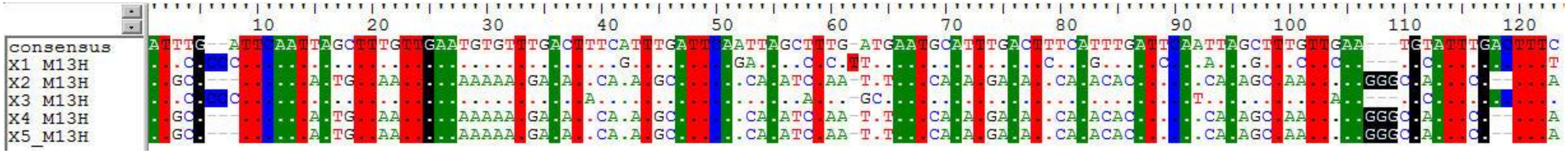

J

clones	CvulCl-112-117_consensus	X1_M13H	X2_M13H	X3_M13H	X4_M13H	X5_M13H
CvulCl-112-117_consensus		83.3	51.6	90.7	51.6	51.6
X1_M13H	83.3		49.5	85.0	49.5	49.5
X2_M13H	51.6	49.5		48.3	100.0	100.0
X3_M13H	90.7	85.0	48.3		48.3	48.3
X4_M13H	51.6	49.5	100.0	48.3		100.0
X5_M13H	51.6	49.5	100.0	48.3	100.0	

K

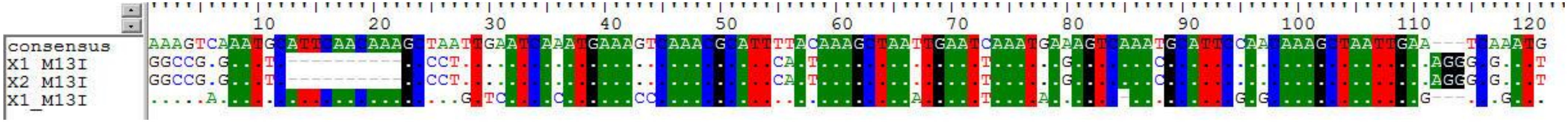

L

clones	CvulCl-134-117_consensus	X1_M13I	X2_M13I	X3_M13I
CvulCl-134-117_consensus		73.5	73.5	87.2
X1_M13I	73.5		100.0	62.8
X2_M13I	73.5	100.0		62.8
X3_M13I	87.2	62.8	62.8	

M

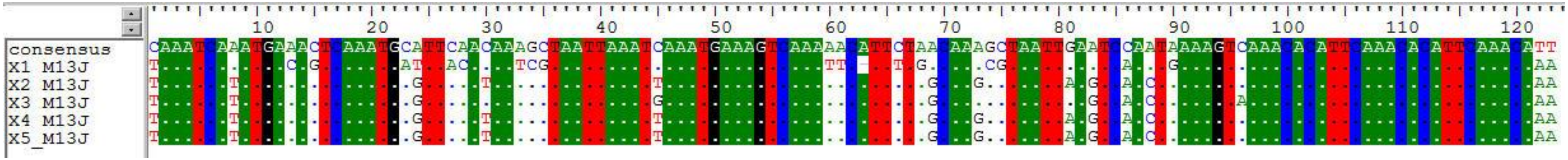

N

clones	Cvul-145-129_consensus	X1_M13J	X2_M13J	X3_M13J	X4_M13J	X5_M13J

Cvul-145-129_consensus		82.9	89.4	91.0	89.4	89.4
X1_M13J	82.9		79.6	81.3	79.6	79.6
X2_M13J	89.4	79.6		95.9	100.0	100.0
X3_M13J	91.0	81.3	95.9		95.9	95.9
X4_M13J	89.4	79.6	100.0	95.9		100.0
X5_M13J	89.4	79.6	100.0	95.9	100.0	
