# Supplementary material for: Natural History of a Satellite DNA Family: From the Ancestral Genome Component to Species-Specific Sequences, Concerted and Non-Concerted Evolution
Source: Int J Mol Sci. 2019 Mar 9;20(5):1201. doi: 10.3390/ijms20051201 (PMC6429384; doi:10.3390/ijms20051201)

Supplementary data 5

Chromosomal distribution CficCl-61-40 satDNA family sequences. CficCl-61-40 is labelled red; *C. acuminatum*-specific HOR unit CacuCl-1-117 of 117 bp is labelled green.

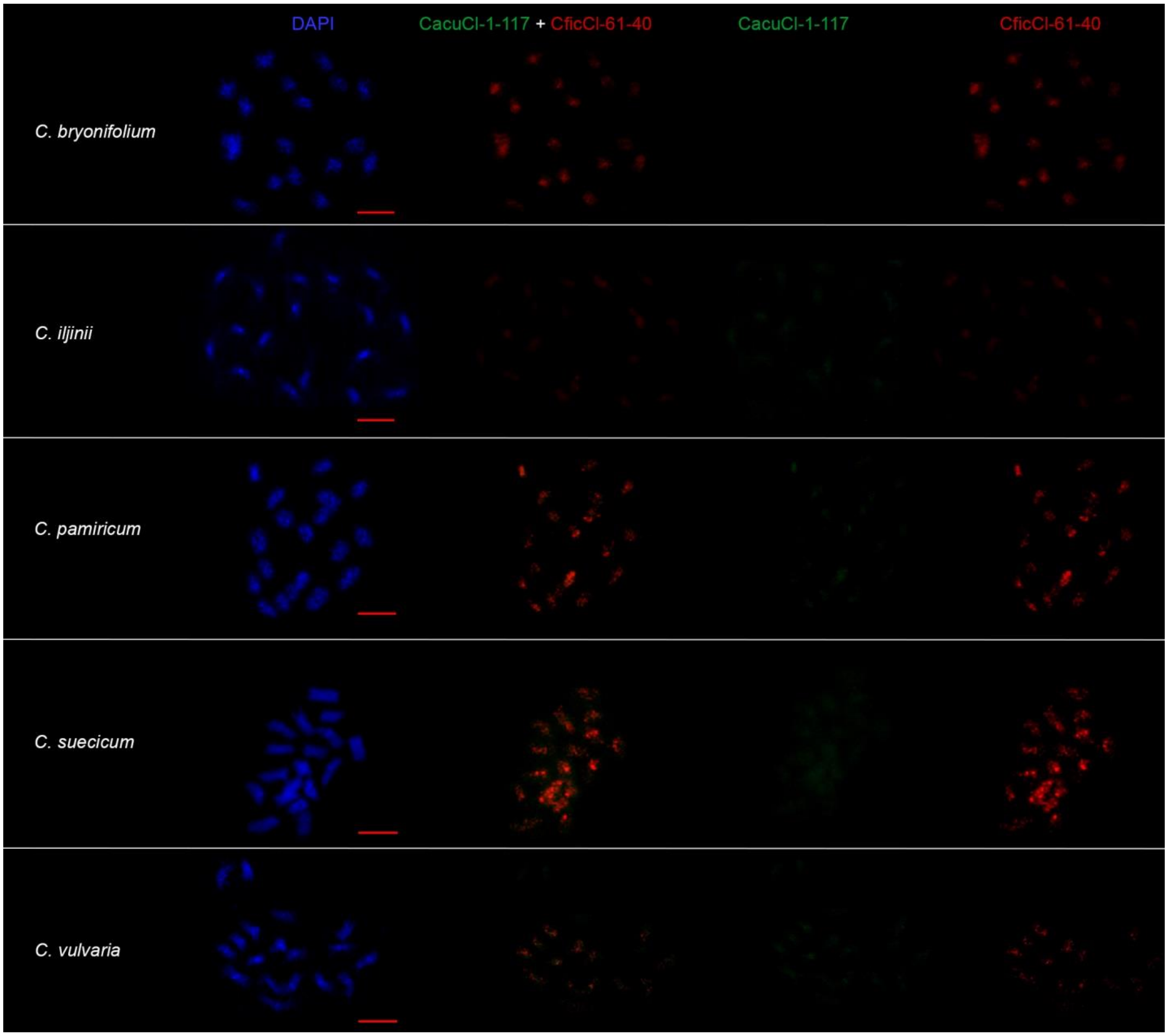

Supplement: Supplementary file 1 [file ijms-20-01201-s001.zip › suppl_Data-5.pdf]
